# Supplementary material for: Seasonal variations in tuberculosis diagnosis among HIV-positive individuals in Southern Africa: analysis of cohort studies at antiretroviral treatment programmes
Source: BMJ Open. 2018 Jan 11;8(1):e017405. doi: 10.1136/bmjopen-2017-017405 (PMC5780693; doi:10.1136/bmjopen-2017-017405)
Supplement: Supplementary file 1 [file bmjopen-2017-017405supp001.pdf]

Supplementary File

Supplementary Figure 1: Quarterly counts of patients newly started on antiretroviral therapy, and quarterly counts of pulmonary tuberculosis diagnoses between 2004 and 2014.

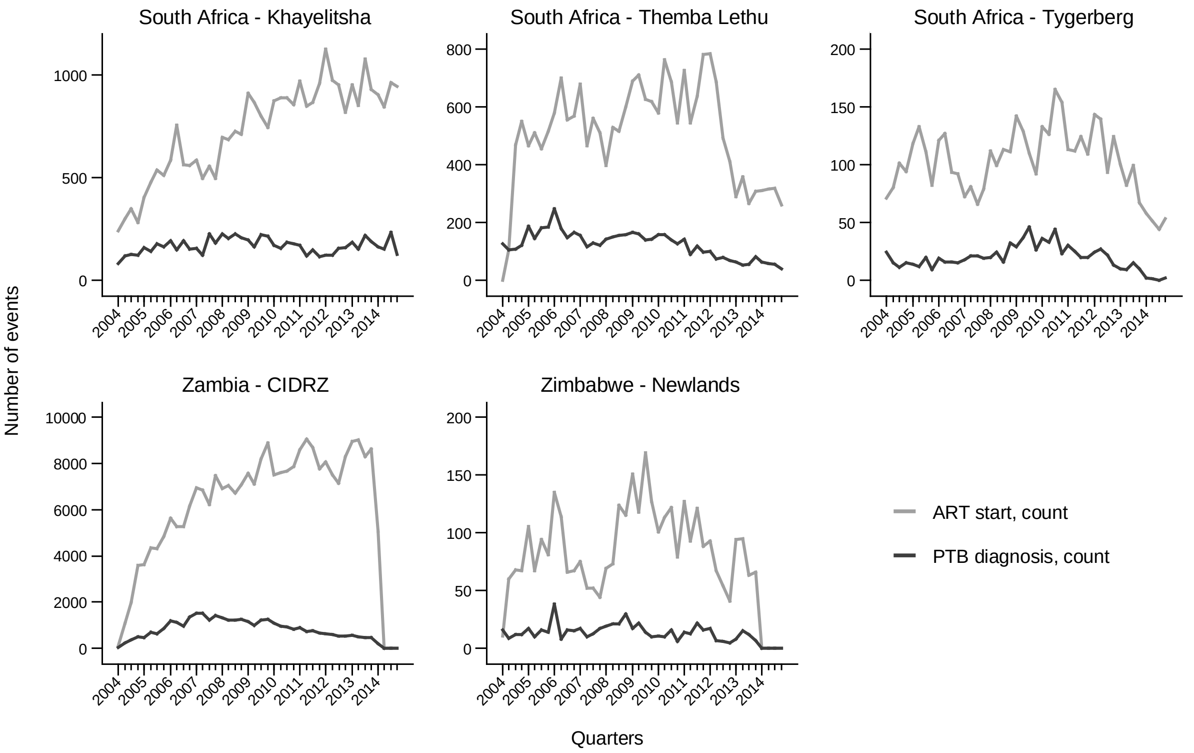

Abbreviations: ART, antiretroviral therapy; PTB, pulmonary tuberculosis

Supplementary Figure 2: Aggregated averages in monthly counts of patients newly started on antiretroviral therapy and monthly counts of pulmonary tuberculosis diagnosis (study period 2004-2014), by gender (**panel A**) and by age groups (**panel B**). Horizontal lines indicate the overall monthly mean of ART enrolment events or PTB diagnosis events.

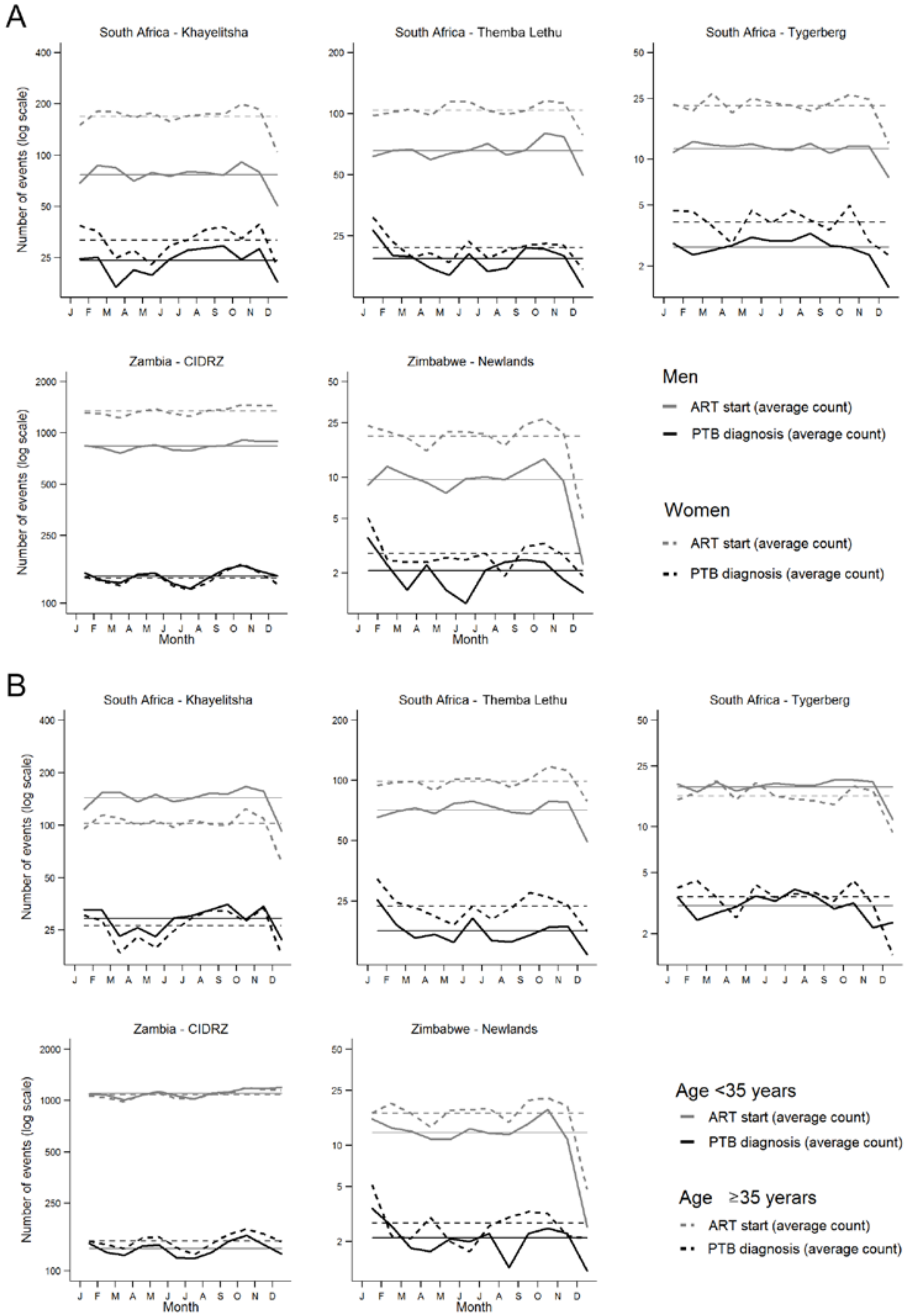

Abbreviations: ART, antiretroviral therapy; PTB, pulmonary tuberculosis.

Supplementary Figure 3: Mean deviations of monthly counts in antiretroviral therapy initiations and pulmonary tuberculosis diagnosis from the corresponding yearly averages.

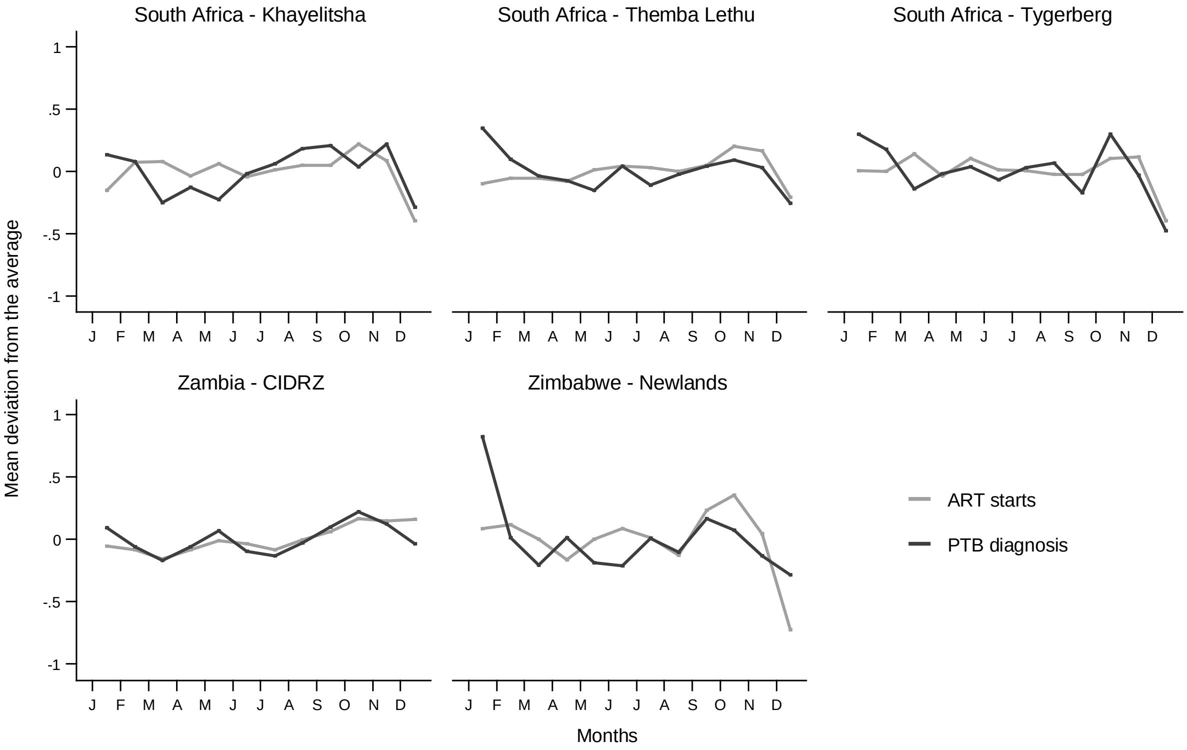

Abbreviations: ART, antiretroviral therapy; PTB, pulmonary tuberculosis
