# Supplementary material for: Seasonal variations in tuberculosis diagnosis among HIV-positive individuals in Southern Africa: analysis of cohort studies at antiretroviral treatment programmes
Source: BMJ Open. 2018 Jan 11;8(1):e017405. doi: 10.1136/bmjopen-2017-017405 (PMC5780693; doi:10.1136/bmjopen-2017-017405)
Supplement: Author's manuscript [file bmjopen-2017-017405.draft_revisions.pdf]

BMJ Open is committed to open peer review. As part of this commitment we make the peer review history of every article we publish publicly available.

When an article is published we post the peer reviewers' comments and the authors' responses online. We also post the versions of the paper that were used during peer review. These are the versions that the peer review comments apply to.

The versions of the paper that follow are the versions that were submitted during the peer review process. They are not the versions of record or the final published versions. They should not be cited or distributed as the published version of this manuscript.

BMJ Open is an open access journal and the full, final, typeset and author-corrected version of record of the manuscript is available on our site with no access controls, subscription charges or pay-per-view fees (http://bmjopen.bmj.com).

If you have any questions on BMJ Open's open peer review process please email editorial.bmjopen@bmj.com

BMJ Open

Seasonal variations in tuberculosis diagnosis among HIV-coinfected individuals in Southern Africa: an observational cohort study at antiretroviral treatment programs

Journal:	*BMJ Open*
Manuscript ID	bmjopen-2017-017405
Article Type:	Research
Date Submitted by the Author:	25-Apr-2017

Complete List of Authors:	Ballif, Marie; University of Bern, Insitute of Social and Preventive Medicine<br>Zürcher, Kathrin; University of Bern, Insitute of Social and Preventive Medicine<br>Reid, Stewart; University of Alabama at Birmingham; Centre for Infectious Disease Research in Zambia<br>Boulle, Andrew; University of Cape Town; Médecins Sans Frontières<br>Fox, Matt; Boston University, Epidemiology and Global Health; University of the Witwatersrand<br>Prozesky, Hans; Division of Infectious Diseases, Department of Medicine, University of Stellenbosch & Tygerberg Academic Hospital<br>Chimbetete, Cleophas; Newlands Clinic<br>Zwahlen , Marcel; University of Bern, Insitute of Social and Preventive Medicine<br>Egger, Matthias; University of Bern, Institute of Social and Preventive Medicine; School of Public Health and Family Medicine, University of Cape Town<br>Fenner, Lukas; University of Bern, Institute of Social and Preventive Medicine

**Primary Subject Heading**:	HIV/AIDS
Secondary Subject Heading:	Infectious diseases, Epidemiology
Keywords:	Seasonality, pulmonary tuberculosis, HIV & AIDS < INFECTIOUS DISEASES, South Africa, Zambia, Zimbabwe

SCHOLARONE™ Manuscripts

TITLE PAGE

Seasonal variations in tuberculosis diagnosis among HIV-coinfected individuals in Southern Africa: an observational cohort study at antiretroviral treatment programs

Marie Ballif [1 §], Kathrin Zürcher [1], Stewart E. Reid [2 3], Andrew Boulle [4 5], Matthew P. Fox [6 7], Hans W. Prozesky [8], Cleophas Chimbetete [9], Marcel Zwahlen [1], Matthias Egger [1 4], Lukas Fenner [1 10 11]; for the International Epidemiology Database to Evaluate AIDS in Southern Africa (IeDEA-SA)

1 Institute of Social and Preventive Medicine, University of Bern, Bern, Switzerland

2 University of Alabama at Birmingham, Birmingham, USA

3 Centre for Infectious Disease Research in Zambia (CIDRZ), Lusaka, Zambia

4 Centre for Infectious Disease Epidemiology and Research (CIDER), School of Public Health and Family Medicine, University of Cape Town, Cape Town, South Africa

5 Médecins Sans Frontières, Khayelitsha, Cape Town, South Africa

6 Health Economics and Epidemiology Research Office, Department of Internal Medicine, School of Clinical Medicine, Faculty of Health Sciences, University of the Witwatersrand, Johannesburg, South Africa

7 Departments of Epidemiology and Global Health, Boston University, Boston, USA

8 Division of Infectious Diseases, Department of Medicine, University of Stellenbosch and Tygerberg Hospital, Cape Town, South Africa

9 Newlands Clinic, Harare, Zimbabwe

10 Swiss Tropical and Public Health Institute, Basel, Switzerland

11 University of Basel, Basel, Switzerland

For peer review only

§ **Corresponding author:**

Dr Marie Ballif

Institute of Social and Preventive Medicine, University of Bern

Finkenhubelweg 11, 3012 Bern, Switzerland

Email: marie.ballif@ispm.unibe.ch

Phone: +41 31 631 35 18 Fax: +41 31 631 35 20

Keywords:

Seasonality, pulmonary tuberculosis, antiretroviral therapy, HIV, South Africa, Zambia, Zimbabwe

Word count:

Main text 1968 (max. 4000), abstract 299 (max. 300), references: 37

Inserts:

2 figures and 2 tables, Supplementary File (3 figures)

For peer review only - http://bmjopen.bmj.com/site/about/guidelines.xhtml

Abstract

Objectives Seasonal variations in tuberculosis diagnoses have been attributed to seasonal climatic changes and indoor crowding during colder winter months. We investigated trends in pulmonary tuberculosis (PTB) diagnosis at antiretroviral therapy (ART) programs in Southern Africa.

Setting Five ART programs participating in the International Epidemiology Database to Evaluate AIDS in South Africa, Zambia, and Zimbabwe.

Participants We analyzed data of 331,634 HIV-positive adults (>15 years), who initiated ART between January 2004 and December 2014.

Primary outcome measure We calculated aggregated averages in monthly counts of PTB diagnoses and ART initiations. To account for time trends, we compared deviations of monthly event counts to yearly averages, and calculated correlation coefficients. We used multivariable linear regressions to assess associations between deviations of monthly ART initiation and of PTB diagnosis counts from yearly averages, adjusted for monthly temperatures. As controls, we used Kaposi sarcoma and extrapulmonary tuberculosis diagnoses.

Results All ART programs showed monthly variations in PTB diagnoses that paralleled fluctuations in ART initiations, with recurrent patterns across 2004-2014. The strongest drops in PTB diagnoses occurred in December, followed by April-May in Zimbabwe and South Africa. This corresponded to holiday seasons, when clinical activities are reduced. We observed little variation in ART initiations and PTB diagnoses throughout the year at the Zambian program. Correlation coefficients supported parallel trends in ART initiations and PTB diagnoses (correlation coefficient: 0.28, 95% confidence interval: 0.21-0.35, p-value <0.001). Monthly temperatures did not substantially change regression coefficients between ART initiations and PTB diagnoses. Trends in Kaposi sarcoma and extrapulmonary tuberculosis diagnoses similarly followed changes in ART initiations throughout the year.

Conclusions Monthly variations in PTB diagnosis at ART programs in Southern Africa likely occurred regardless of seasonal variations in temperatures and reflected fluctuations in clinical

For peer review only

For peer review only - http://bmjopen.bmj.com/site/about/guidelines.xhtml

activities and changes in health-seeking behaviour throughout the year, rather than climatic factors.

Strengths and limitations of this study

- We analyzed a large dataset from HIV-positive patients routinely collected over 10 years at ART programs in low and middle income countries from the Southern African region;
- Included ART programs represent a wide range of countries with different climatic seasons according to their geographical latitude;
- We compared trends in PTB diagnosis to trends in Kaposi sarcoma and extrapulmonary tuberculosis diagnoses for which no seasonal effect is expected;
- Our study is limited by the possible under-ascertainment of tuberculosis diagnoses at ART programs;
- Trends in tuberculosis diagnosis and other opportunistic infections in HIV-positive may differ from trends in HIV-negative populations.

For peer review only

For peer review only - http://bmjopen.bmj.com/site/about/guidelines.xhtml

Introduction

Countries in different regions have reported seasonal fluctuations in diagnoses of tuberculosis (TB).[1–4] In temperate climate zones, peaks in TB diagnosis are generally observed in the spring, and troughs in the fall, moving in parallel with seasonal climatic changes. The causes of these variations are multiple, with both social and environmental factors likely playing a role.[5] Peaks in TB diagnosis after winter have been associated with increased time indoors (which increases risk of transmission), poorer access to health care in remote areas, and lower vitamin D levels due to reduced exposure to sunlight.[1,3,5–7]

The burden of HIV and TB in Southern Africa is among the highest worldwide.[8–10] However, little is known about the seasonal variation of TB diagnoses in the context of antiretroviral therapy (ART) programs. We hypothesized that seasonal climate fluctuations in TB diagnoses would likely be stronger in countries located farther away from the equator and that experience more pronounced climatic changes. We investigated seasonal trends in pulmonary TB (PTB) diagnosis at five antiretroviral treatment (ART) programs located at different latitudes, with different climates, in South Africa, Zambia, and Zimbabwe.

For peer review only

Methods

We included all HIV-positive persons age 16 and older who enrolled between January 1, 2004 and December 31, 2014 in five ART programs that systematically collect information on opportunistic infections (OIs) and collaborate with the International Epidemiology Databases to Evaluate AIDS research consortium in Southern Africa (IeDEA-SA):[11] Khayelitsha ART program, Themba Lethu clinic, and Tygerberg Academic Hospital in South Africa; Centre for Infectious Disease Research in Zambia (CIDRZ); and Newlands Clinic in Zimbabwe. IeDEA-SA is a regional collaboration of ART programs that is part of the larger international network.[11] All study sites have approval from a local institutional review board or ethics committee to collect data and participate in IeDEA-SA projects. The patient selection is shown in the supplemental digital content (SDC) Figure 1.

PTB was defined according to the World Health Organization guidelines and case definitions in use at participating sites.[12,13] We restricted the main analysis to PTB, because the diagnosis of PTB has been associated with seasonal climatic variations.[1,2,5] For comparison, we analysed monthly counts of patients diagnosed with extrapulmonary TB (EPTB) and the AIDS-defining cancer Kaposi sarcoma (KS), not expected to be seasonal. One record per patient was analysed. If a patient had several episodes of the same disease (PTB, EPTB, or KS), we only considered the first event of each. If a patient was diagnosed with multiple diseases of interest, priority was given to the record with PTB diagnosis, followed by EPTB and KS. We obtained mean monthly air temperatures (°C) based on 1961–1990 averages at each location.[14–17]

For each site, we calculated aggregated averages as the average number of events per calendar month for 2004-2014. We accounted for calendar time trends over the study period by comparing deviations of monthly event counts to yearly averages. Site-specific aggregated averages and monthly deviations were calculated for ART enrolments and for PTB, EPTB, and KS diagnoses, overall or stratified by gender and age. We assessed the association between monthly deviations in the number of ART enrolments and the number of PTB diagnoses by calculating pairwise Pearson's correlation coefficients.[18] We used Fisher's transformation to calculate 95% confidence intervals (CI) for the correlation coefficient. Finally, we used

For peer review only - http://bmjopen.bmj.com/site/about/guidelines.xhtml

multivariable linear regression models to assess associations between the monthly deviations of PTB, EPTB or KS diagnosis counts and the monthly deviations of ART initiation counts to account for monthly deviations of temperatures from yearly averages.

All analyses were performed in Stata version 14.1 (Stata Corporation, College Station, TX, USA).

For peer review only

For peer review only - http://bmjopen.bmj.com/site/about/guidelines.xhtml

Results

We analysed data of 331,634 HIV-positive adults (SDC Figure 1). Of these 49,367 (14.9%) were diagnosed with PTB. Median follow-up time was 2.0 years (interquartile range [IQR], 0.7-4.2), patients' median age was 34 years (IQR, 29-41), and 207,074 (62.4%) were women (Table 1). Median CD4 cell count at ART initiation of these patients was 116 cells/mm^3 (IQR: 52-200); this compares to a median cell count of 166 (87-254) in patients who were never diagnosed with PTB.

The mean number of PTB diagnoses per month was 55.7 (range 40.2-67.8) in Khayelitsha, 41.2 (31.1-57.6) in Themba Lethu, 6.5 (3.8-7.7) in Tygerberg, 285.6 (243.2-338.2) in CIDRZ, and 4.8 (3.3-8.7) in Newlands (Figure 1). At each site, monthly variations in numbers of PTB diagnoses were parallel to trends in ART enrolments. Aggregated averages over the study period showed that monthly fluctuations in PTB diagnoses were present at all sites (Figure 1). The strongest fluctuations were seen in Zimbabwe (Newlands), with PTB diagnosis peaks in January and troughs in December (+87% and -33% compared to the monthly average, respectively). Similar to Zimbabwe, all three South African sites showed marked drops in ART initiations and PTB diagnoses in December, in addition to a milder drop in April-May. In contrast, Zambia (CIDRZ) showed little variation in ART enrolments and PTB diagnoses throughout the year. We consistently observed a similar seasonal pattern in patients newly starting ART and PTB diagnoses every year (SDC Figure 2), in men and women, as well as in younger and older patients (Figure 2).

The deviations of monthly event counts from the yearly averages confirmed the congruent fluctuations of ART initiation and PTB diagnosis (SDC Figure 3). This was further supported by the correlation coefficients between the deviations of monthly ART initiations from yearly averages and the deviations of monthly PTB diagnoses from yearly averages (Table 1): coefficient was 0.27 in Khayelitsha (95% confidence interval [CI] 0.11-0.42, p=0.002), 0.22 in Themba Lethu (95% CI 0.05-0.38, p=0.010), 0.19 in Tygerberg (95% CI 0.02-0.35, p=0.028), 0.82 in CIDRZ (95% CI 0.75-0.87, p-value<0.001), and 0.20 in Newlands (95% CI 0.02-0.37, p=0.029). Regressions coefficients between deviations of monthly PTB diagnosis counts and monthly ART

For peer review only - http://bmjopen.bmj.com/site/about/guidelines.xhtml

initiation counts from yearly averages did not substantially change when adjusted for monthly temperature (Table 2).

To determine whether the diagnosis of other OIs followed trends similar to that of PTB and ART initiations, we studied the monthly diagnoses of EPTB and KS, which we did not expect to be influenced by climatic factors. The seasonal pattern of EPTB diagnoses paralleled that of ART initiation at most sites despite low numbers of EPTB diagnoses (1.8 to 26.5 mean monthly EPTB diagnoses). The deviations of monthly ART initiations from the yearly averages correlated with those of EPTB diagnoses in Khayelitsha, CIDRZ, and Newlands (correlation coefficient range: 0.20-0.54, p=0.019, Table 1). Although KS was a rare diagnosis averaging 1.1 cases per month overall, we observed that the fluctuations in KS diagnosis counts similarly followed changes in ART enrolment at all sites (Table 1). As for PTB diagnoses, regressions models adjusted for monthly temperature did not support any influence of temperatures on the observed fluctuations in EPT or KS diagnoses (Table 2).

For peer review only

For peer review only - http://bmjopen.bmj.com/site/about/guidelines.xhtml

Discussion

We observed monthly variations in PTB diagnosis at ART programs in South Africa, Zambia and Zimbabwe. These fluctuations followed trends in ART enrolment rather than seasonal changes in temperatures, and hence mirrored fluctuations in clinical activity. In South Africa and Zimbabwe, a marked drop of ART enrolments and PTB diagnoses was seen in December, followed by a rebound in January-February. A milder drop was also observed in South African sites around April-May.

In Southern Africa, seasons are marked by cooler winters and warmer summers in Cape Town (Khayelitsha, Tygerberg), and become less pronounced as latitude decreases, in Johannesburg (Themba Lethu), Zimbabwe and Zambia. Therefore, we expected to see stronger fluctuations in PTB diagnosis in the Western Cape (South Africa) than in Zambia and Zimbabwe, with peaks following winter. Instead, we observed variations in PTB that seemed independent from climatic changes. This might be explained by mild winters in Southern Africa, contrasting with countries with harsh winters. Mongolia typically has cold winters and strong seasonal patterns in TB notifications, likely due to increased airborne transmission from infectious PTB individuals resulting from indoor crowding and poorer ventilation during winter.[1,2] Nevertheless, seasonal variations in TB notifications in a population-based study of HIV-positive and HIV-negative individuals were documented in Cape Town, South Africa.[6]

We observed that variations in PTB diagnoses followed fluctuations in ART initiations and hence likely reflected activities at the clinic, rather than any seasonal, climatic effects. This was supported by an association between changes in ART initiation and PTB diagnosis counts (unadjusted and adjusted for monthly climatic temperature). Furthermore, important drops in ART initiations and PTB diagnoses were observed every year in December in South Africa and Zimbabwe, when clinical activities are reduced due to the holiday season. December drops were followed by peaks in January and February, when clinical activity resumed. Similar declines in PTB diagnoses at the end of the year have been reported in Zimbabwe and Uganda by Mabaera et al.[2] In CIDRZ, the largest of the cohorts we studied, monthly fluctuations in ART initiations and PTB diagnoses were the least marked as compared to the South African and Zimbabwean sites. This may be due to the shorter December holiday period in the Zambian health sector, and

For peer review only - http://bmjopen.bmj.com/site/about/guidelines.xhtml

likewise among for HIV-positive individuals seeking care at ART programs. Similarly, Easter vacations likely explain the milder drop observed in South Africa in April-May. Age and sex did not seem to influence the seasonal variations.

EPTB and KS diagnoses also followed variations in ART initiations in our study. We did not expect the development of EPTB to be strongly marked by seasonal changes. Indeed, EPTB is less likely resulting from recent transmission than PTB, the transmission mode of which is airborne.[1,5] However, seasonal changes in EPTB case detection have been reported.[3,4,19] The underlying reasons for EPTB seasonality may considerably differ from those involved in PTB, since EPTB cases are generally strongly dependent on the patient's immunocompetence.[3,19] Similarly, KS was not expected to be influenced by seasonal factors, since the transmission of the viral agent is not known to be climate-dependent. The seasonal fluctuations of EPTB and KS diagnoses hence reflect variations in clinical activities, such as observed for PTB.

Several explanations of why PTB diagnoses may vary seasonally have been offered.[1,3,5–7] Beyond indoor crowding[1,7] and reduced clinical activities at specific times during the year,[2] health-seeking behaviour is also influencing access to care. Health-seeking behaviour might reflect environmental barriers (long distances to the clinic, waiting time),[20,21] psychological barriers (stigma),[22] and/or clinical barriers (poor patient's health status and disabilities).[23] Access to clinics can be problematic when temperatures are low and weather conditions are bad. Under these circumstances, PTB diagnosis might be delayed to spring. Professional activities or beliefs may also prevent early care seeking. Economical aspects play an important role, where care is to be fully or partly paid by patients.[12] These aspects might differently affect individuals, depending on gender or age.[24–27] However, in our study populations, trends in ART initiations and PTB diagnoses were independent from gender or age. Finally, the role of vitamin D deficiency has been debated, and studies of the relationship between vitamin D deficiency during winter and PTB are inconsistent.[3,28–31]

Our study is limited by the possible under-ascertainment of TB diagnoses at ART programs, and by the fact that we could only include IeDEA-SA sites that routinely collect information on OIs. Furthermore, trends in TB diagnosis and other OIs at ART programs may differ from trends in HIV-negative populations. Social and environmental factors, including

For peer review only - http://bmjopen.bmj.com/site/about/guidelines.xhtml

access to care, disease awareness, and health-seeking behaviour may differently affect the timely diagnosis of PTB in HIV-negative and HIV-positive populations.[1,5,7,32,33]

Conclusions

In conclusion, our study suggests that yearly trends in PTB diagnosis at ART programs in Southern Africa are mainly driven by seasonal variations in clinical activities, access to care and health-seeking behaviour, rather than climatic seasons. Our results underline the importance of offering regular care for HIV-infected individuals, including screenings for OIs such as TB at ART initiation and during follow-up visits, as part of integrated TB/HIV clinical activities at ART programs.[34–37]

For peer review only

For peer review only - http://bmjopen.bmj.com/site/about/guidelines.xhtml

Funding

This work was supported by the National Institute of Allergy and Infectious Diseases (NIAID), the National Institute of Child Health and Human Development (NICHD), and the National Cancer Institute (NCI) under award number U01AI069924. MPF was supported by USAID 674-A-12-00029 from the United States Agency for International Development. The opinions expressed herein are those of the authors and do not necessarily reflect the views of the funder. The funder had no role in study design, data collection and analysis, decision to publish, or preparation of the manuscript.

Competing interests

The authors declare no conflicts of interest directly related to this project. MZ discloses the following unrelated funding sources: MZ has received grant support from the Swiss National Science Foundation and from the Swiss Cancer league which paid for PhD and postdoc positions. Furthermore, MZ has consulted for BIOTRONIK AG, Switzerland, and has projects which received unrestricted research support from following firms: AstraZeneca AG, Switzerland, Aptalis Pharma Inc., Dr. Falk Pharma GmbH, Germany, Glaxo Smith Kline AG, Nestlé S. A., Switzerland, Receptos Inc., Regeneron Inc. For the remaining authors none were declared.

Authors' contributions

MB analysed the data and completed the final draft of the manuscript. LF conceptualized the project. MZ established the methodology. KZ, SER, AB, MPF, HWP, CC and ME provided input into the study design, analyses, and drafting of the paper. All authors reviewed and approved the final version of the manuscript.

Acknowledgements

We thank all participating sites and patients whose data were used in this study. We also would like to thank collaborators who contributed to recording and entering data, as well as the data centres of the IeDEA-SA collaboration for preparing the data. We thank Christopher Ritter for editorial services.

For peer review only

IeDEA-Southern Africa Steering Committee

Matthias Egger (co-PI), University of Bern, Switzerland; **Mary-Ann Davies** (co-PI), University of Cape Town, South Africa**; Frank Tanser**, Africa Centre for Health and Population Studies, University of Kwazulu-Natal, South Africa; **Michael Vinikoor**, Centre for Infectious Disease Research in Zambia; **Eusebio Macete**, Centro de Investigação em Saúde de Manhiça, Mozambique; **Robin Wood**, Desmond Tutu HIV Centre (Gugulethu and Masiphumelele clinics), South Africa**; Kathryn Stinson**, Khayelitsha ART Programme and Médecins Sans Frontières, South Africa; **Geoffrey Fatti**, Kheth'Impilo Programme, South Africa; **Sam Phiri**, Lighthouse Trust Clinic, Malawi; **Cleophas Chimbetete**, Newlands Clinic, Zimbabwe; **Kennedy Malisita**, Queen Elizabeth Hospital, Malawi; **Brian Eley**, Red Cross War Memorial Children's Hospital and Department of Paediatrics and Child Health, University of Cape Town, South Africa; **Jochen Ehmer**, Solidarmed, Switzerland; **Christiane Fritz**, SolidarMed SMART Programme, Lesotho; **Michael Hobbins**, SolidarMed SMART Programme, Mozambique; **Kamelia Kamenova**, SolidarMed SMART Programme, Zimbabwe; **Matthew Fox**, Themba Lethu Clinic, South Africa; **Hans Prozesky**, Tygerberg Academic Hospital, South Africa; **Karl Technau**, Empilweni Clinic, Rahima Moosa Mother and Child Hospital, South Africa; **Shobna Sawry**, Harriet Shezi Children's Clinic, Chris Hani Baragwanath Academic Hospital, South Africa.

For peer review only

References

1 Naranbat N, Nymadawa P, Schopfer K, *et al.* Seasonality of tuberculosis in an Eastern-Asian country with an extreme continental climate. *Eur Respir J* 2009;**34**:921–5. doi:10.1183/09031936.00035309

2 Mabaera B, Naranbat N, Katamba A, *et al.* Seasonal variation among tuberculosis suspects in four countries. *Int Health* 2009;**1**:53–60. doi:10.1016/j.inhe.2009.06.006

3 Willis MD, Winston CA, Heilig CM, *et al.* Seasonality of tuberculosis in the United States, 1993-2008. *Clin Infect Dis Off Publ Infect Dis Soc Am* 2012;**54**:1553–60. doi:10.1093/cid/cis235

4 Thorpe LE, Frieden TR, Laserson KF, *et al.* Seasonality of tuberculosis in India: is it real and what does it tell us? *Lancet* 2004;**364**:1613–4. doi:10.1016/S0140-6736(04)17316-9

5 Fares A. Seasonality of tuberculosis. *J Glob Infect Dis* 2011;**3**:46–55. doi:10.4103/0974-777X.77296

6 Martineau AR, Nhamoyebonde S, Oni T, *et al.* Reciprocal seasonal variation in vitamin D status and tuberculosis notifications in Cape Town, South Africa. *Proc Natl Acad Sci U S A* 2011;**108**:19013–7. doi:10.1073/pnas.1111825108

7 Wingfield T, Schumacher SG, Sandhu G, *et al.* The seasonality of tuberculosis, sunlight, vitamin d, and household crowding. *J Infect Dis* 2014;**210**:774–83. doi:10.1093/infdis/jiu121

8 Corbett EL, Watt CJ, Walker N, *et al.* The growing burden of tuberculosis: global trends and interactions with the HIV epidemic. *Arch Intern Med* 2003;**163**:1009–21. doi:10.1001/archinte.163.9.1009

9 Pawlowski A, Jansson M, Skold M, *et al.* Tuberculosis and HIV co-infection. *PLoS Pathog* 2012;**8**:e1002464. doi:10.1371/journal.ppat.1002464

10 World Health Organization. Global tuberculosis report 2015. WHO 2015. http://www.who.int/tb/publications/global_report/en/

11 Egger M, Ekouevi DK, Williams C, *et al.* Cohort Profile: the international epidemiological databases to evaluate AIDS (IeDEA) in sub-Saharan Africa. *Int J Epidemiol* 2012;**41**:1256–64. doi:10.1093/ije/dyr080

12 Fenner L, Ballif M, Graber C, *et al.* Tuberculosis in antiretroviral treatment programs in lower income countries: availability and use of diagnostics and screening. *PloS One* 2013;**8**:e77697. doi:10.1371/journal.pone.0077697

13 World Health Organization. Definitions and reporting framework for tuberculosis – 2013 revision (updated December 2014). 2013.http://apps.who.int/iris/bitstream/10665/79199/1/9789241505345_eng.pdf

For peer review only - http://bmjopen.bmj.com/site/about/guidelines.xhtml

14 Cape Town. Wikipedia. 2016.https://en.wikipedia.org/w/index.php?title=Cape_Town&oldid=748785698 (accessed 10 Nov 2016).

15 Johannesburg. Wikipedia. 2016.https://en.wikipedia.org/w/index.php?title=Johannesburg&oldid=748810210 (accessed 10 Nov 2016).

16 Harare. Wikipedia. 2016.https://en.wikipedia.org/w/index.php?title=Harare&oldid=746466670 (accessed 10 Nov 2016).

17 Lusaka. Wikipedia. 2016.https://en.wikipedia.org/w/index.php?title=Lusaka&oldid=748771985 (accessed 10 Nov 2016).

18 Altman DG. *Practical Statistics for Medical Research - Relationship between two continuous variables*. Chapman and Hall/CRC. 1990.

19 Top R, Boshuizen H, Dekkers A, *et al.* Similar seasonal peak in clustered and unique extra-pulmonary tuberculosis notifications: winter crowding hypothesis ruled out? *Int J Tuberc Lung Dis Off J Int Union Tuberc Lung Dis* 2013;**17**:1466–71. doi:10.5588/ijtld.13.0226

20 Schwitters A, Lederer P, Zilversmit L, *et al.* Barriers to Health Care in Rural Mozambique: A Rapid Ethnographic Assessment of Planned Mobile Health Clinics for ART. *Glob Health Sci Pract* 2015;**3**:109–16. doi:10.9745/GHSP-D-14-00145

21 Johnson DC, Feldacker C, Tweya H, *et al.* Factors associated with timely initiation of antiretroviral therapy in two HIV clinics in Lilongwe, Malawi. *Int J STD AIDS* 2013;**24**:42–9. doi:10.1177/0956462412472312

22 Sandelowski M, Barroso J, Voils CI. Gender, Race/Ethnicity, and Social Class in Research Reports on Stigma in HIV-Positive Women. *Health Care Women Int* 2009;**30**:273–88. doi:10.1080/07399330802694880

23 Tun W, Okal J, Schenk K, *et al.* Limited accessibility to HIV services for persons with disabilities living with HIV in Ghana, Uganda and Zambia. *J Int AIDS Soc* 2016;**19**. doi:10.7448/IAS.19.5.20829

24 Getahun H, Gunneberg C, Granich R, *et al.* HIV infection-associated tuberculosis: the epidemiology and the response. *Clin Infect Dis* 2010;**50 Suppl 3**:S201-7. doi:10.1086/651492

25 Braitstein P, Boulle A, Nash D, *et al.* Gender and the use of antiretroviral treatment in resource-constrained settings: findings from a multicenter collaboration. *J Womens Health 2002* 2008;**17**:47–55. doi:10.1089/jwh.2007.0353

For peer review only - http://bmjopen.bmj.com/site/about/guidelines.xhtml

26 Holmes CB, Hausler H, Nunn P. A review of sex differences in the epidemiology of tuberculosis. *Int J Tuberc Lung Dis* 1998;**2**:96–104.

27 Fenner L, Forster M, Boulle A, *et al.* Tuberculosis in HIV programmes in lower-income countries: practices and risk factors. *Int J Tuberc Lung Dis* 2011;**15**:620–7. doi:10.5588/ijtld.10.0249

28 Cegielski P, Vernon A. Tuberculosis and vitamin D: what's the rest of the story? *Lancet Infect Dis* 2015;**15**:489–90. doi:10.1016/S1473-3099(15)70163-5

29 Daley P, Jagannathan V, John KR, *et al.* Adjunctive vitamin D for treatment of active tuberculosis in India: a randomised, double-blind, placebo-controlled trial. *Lancet Infect Dis* 2015;**15**:528–34. doi:10.1016/S1473-3099(15)70053-8

30 Coussens AK, Wilkinson RJ, Hanifa Y, *et al.* Vitamin D accelerates resolution of inflammatory responses during tuberculosis treatment. *Proc Natl Acad Sci U S A* 2012;**109**:15449–54. doi:10.1073/pnas.1200072109

31 Ralph AP, Waramori G, Pontororing GJ, *et al.* L-arginine and Vitamin D Adjunctive Therapies in Pulmonary Tuberculosis: A Randomised, Double-Blind, Placebo-Controlled Trial. *PLOS ONE* 2013;**8**:e70032. doi:10.1371/journal.pone.0070032

32 Baker M, Das D, Venugopal K, *et al.* Tuberculosis associated with household crowding in a developed country. *J Epidemiol Community Health* 2008;**62**:715–21. doi:10.1136/jech.2007.063610

33 Lönnroth K, Jaramillo E, Williams BG, *et al.* Drivers of tuberculosis epidemics: the role of risk factors and social determinants. *Soc Sci Med 1982* 2009;**68**:2240–6. doi:10.1016/j.socscimed.2009.03.041

34 Lessells RJ, Swaminathan S, Godfrey-Faussett P. HIV treatment cascade in tuberculosis patients. *Curr Opin HIV AIDS* 2015;**10**:439–46. doi:10.1097/COH.0000000000000197

35 Tweya H, Feldacker C, Gadabu OJ, *et al.* Developing a point-of-care electronic medical record system for TB/HIV co-infected patients: experiences from Lighthouse Trust, Lilongwe, Malawi. *BMC Res Notes* 2016;**9**:146. doi:10.1186/s13104-016-1943-4

36 Charles MK, Lindegren ML, Wester CW, *et al.* Implementation of Tuberculosis Intensive Case Finding, Isoniazid Preventive Therapy, and Infection Control ("Three I's") and HIV-Tuberculosis Service Integration in Lower Income Countries. *PloS One* 2016;**11**:e0153243. doi:10.1371/journal.pone.0153243

37 World Health Organization. WHO policy on collaborative TB/HIV activities: guidelines for national programmes and other stakeholders. Published Online First: 2012.http://www.who.int/tb/publications/2012/tb_hiv_policy_9789241503006/en/index.html

For peer review only

For peer review only - http://bmjopen.bmj.com/site/about/guidelines.xhtml

For peer review only

For peer review only - http://bmjopen.bmj.com/site/about/guidelines.xhtml

Figure and tables

Table 1: Characteristics of patients diagnosed with pulmonary tuberculosis between January 2004 and December 2014 compared to others.

**Characteristics**	**Patients diagnosed with PTB**	**Other patients**	**All**
**Age at ART start (median, IQR), years**	35 (30-41)	34 (29-41)	34 (29-41)
Missing, n (%)	*0*	*0*	*0*
**Gender, n (%)**			
Female	25,227 (51.1)	181,847 (64.4)	207,074 (62.4)
Male	24,140 (48.9)	100,420 (35.6)	124,560 (37.6)
Missing, n (%)	*0*	*0*	*0*
**CD4 cell count at ART start (median, IQR), cells/µl**	116 (52-200)	166 (87-254)	158 (80-246)
Missing, n (%)	*24,237 (48.8)*	*150,058 (53.2)*	*174,295 (52.6)*
**WHO clinical stage, n (%)**			
Stage 1 and 2	4,916 (9.9)	157,322 (55.7)	162,238 (48.9)
Stage 3 and 4	41,744 (84.6)	106,836 (37.9)	148,580 (44.8)
Unknown	2,707 (5.5)	18,053 (6.4)	20,816 (6.3)
Missing, n (%)	*0*	*56 (0.02)*	*56 (0.02)*
**Treatment program, n (%)**			
South Africa – Khayelitsha	7,357 (14.9)	25,021 (8.9)	32,378 (9.8)
South Africa – Themba Lethu	5,438 (11.0)	16,967 (6.0)	22,405 (6.8)
South Africa – Tygerberg	861 (1.7)	3,657 (1.3)	4,518 (1.4)
Zambia – CIDRZ	35,132 (71.2)	233,680 (82.8)	268,812 (81.1)
Zimbabwe – Newlands	579 (1.2)	2,942 (1.0)	3,521 (1.1)
Missing, n (%)	*0*	*0*	*0*
**Total, n (%)**	**49,367 (14.9)**	**282,267 (85.1)**	**331,634 (100)**

PTB, pulmonary tuberculosis; ART, antiretroviral therapy; IQR, interquartile range; WHO, World Health Organization.

For peer review only

Figure 1: Aggregated averages in monthly counts of patients newly started on antiretroviral therapy and monthly counts of pulmonary tuberculosis diagnosis (aggregate averages 2004-2014, logarithmic scale). Dashed horizontal lines indicate overall monthly means.

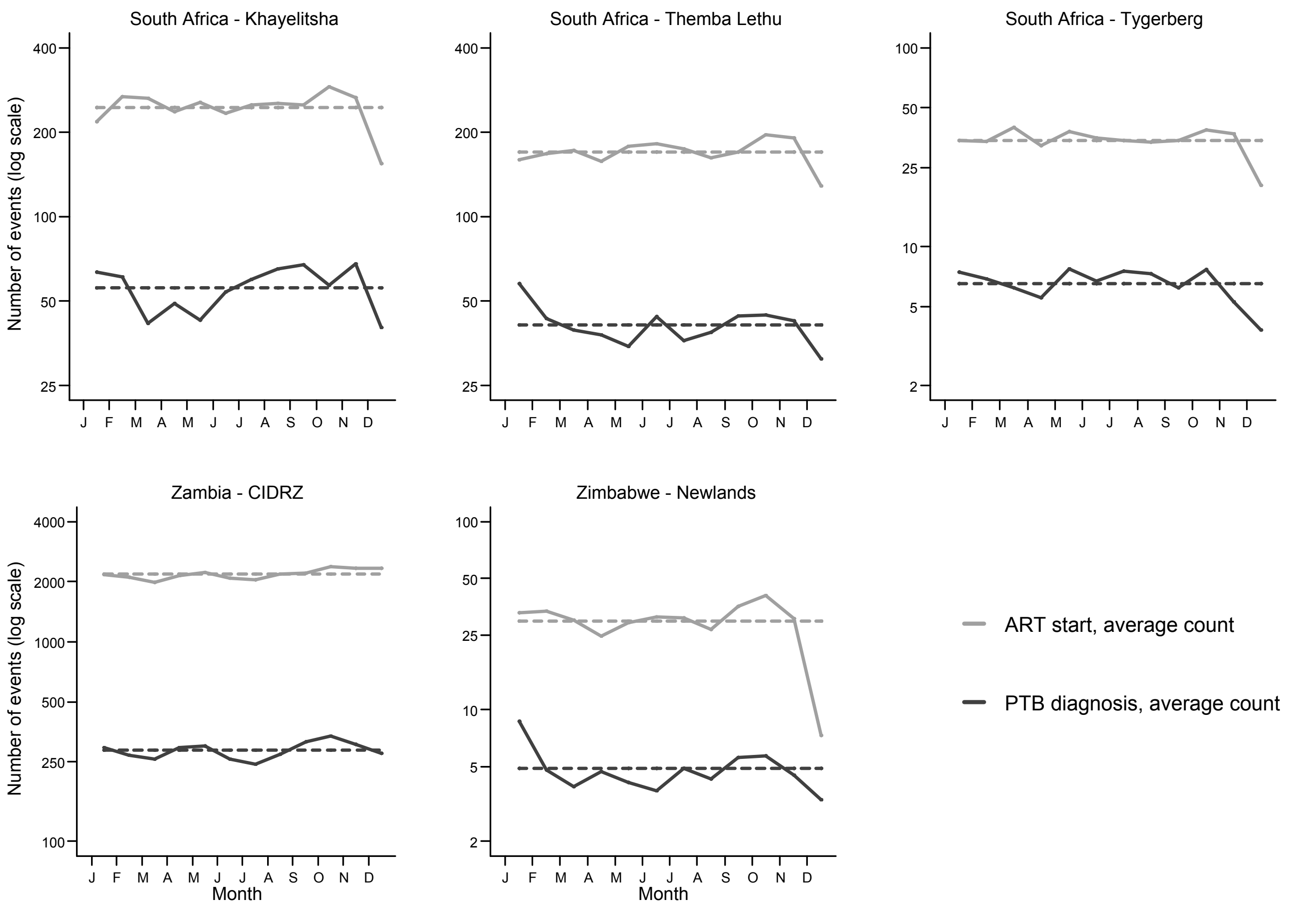

Abbreviations: ART, antiretroviral therapy; PTB, pulmonary tuberculosis

For peer review only - http://bmjopen.bmj.com/site/about/guidelines.xhtml

Figure 2: Aggregated averages in monthly counts of patients newly started on antiretroviral therapy and monthly counts of pulmonary tuberculosis diagnosis (study period 2004-2014), by gender (**panel A**) and by age groups (**panel B**). Horizontal lines indicate the overall monthly mean of ART enrolment events or PTB diagnosis events.

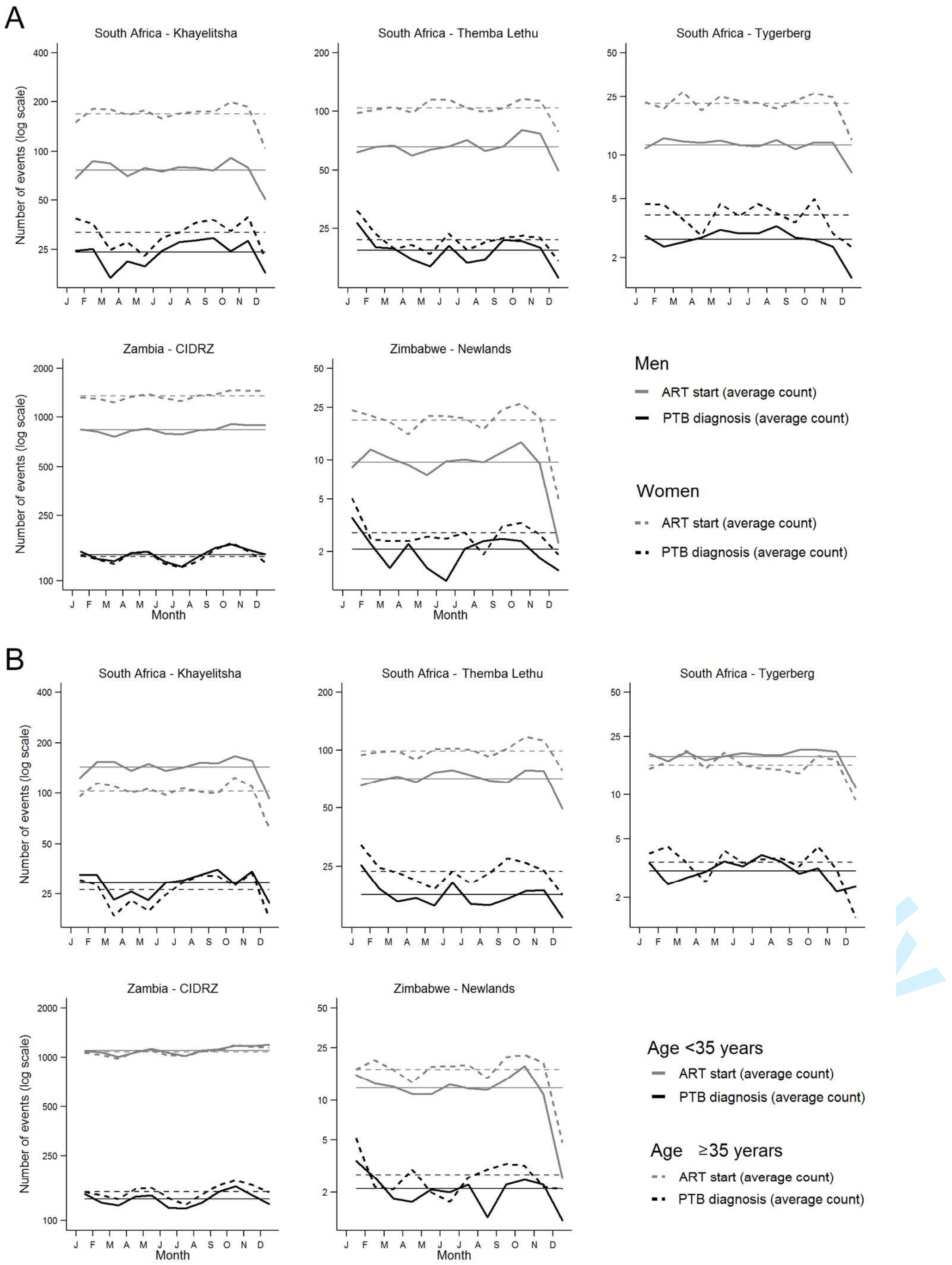

Table 1: Correlation coefficients between deviations of monthly counts of antiretroviral therapy enrolments from the yearly average and deviations of monthly counts of pulmonary tuberculosis, extrapulmonary tuberculosis, and Kaposi sarcoma diagnosis from the yearly averages. Median monthly event counts (2000-2014) and Pearson's pairwise correlation coefficients are shown.

ART program	ART enrollments	PTB diagnoses			EPTB diagnoses			KS diagnoses		
	Median (IQR)	Median (IQR)	Correlation (95% CI)	p-value	Median (IQR)	Correlation (95% CI)	p-value	Median (IQR)	Correlation (95% CI)	p-value
South Africa – Khayelitsha	252 (236-265)	58 (46-64)	0.27 (0.11-0.42)	0.002	26 (24-30)	0.20 (0.03-0.36)	0.019	2 (1-2)	0.08 (-0.11-0.25)	0.42
South Africa – Themba Lethu	171 (161-180)	41 (37-44)	0.22 (0.05-0.38)	0.010	16 (14-17)	0.15 (-0.03-0.31)	0.097	1 (1-1)	0.02 (-0.16-0.19)	0.86
South Africa – Tygerberg	34 (34-37)	7 (6-8)	0.19 (0.02-0.35)	0.028	3 (3-3)	0.09 (-0.09-0.25)	0.33	0.3 (0.2-0.5)	0.11 (-0.06-0.28)	0.22
Zambia – CIDRZ	2163 (2095-2233)	275 (259-301)	0.82 (0.75-0.87)	<0.001	9 (9-10)	0.54 (0.40-0.65)	<0.001	29 (28-31)	0.68 (0.57-0.77)	<0.001
Zimbabwe – Newlands	31 (29-34)	5 (4-6)	0.20 (0.02-0.37)	0.029	2 (2-2)	0.23 (0.06-0.40)	0.011	0.2 (0.1-0.4)	0.22 (0.05-0.39)	0.014
**All**	**170 (34-264)**	**40 (6-63)**	**0.28 (0.21-0.35)**	**<0.001**	**9 (3-17)**	**0.25 (0.18-0.32)**	**<0.001**	**1 (0-2)**	**0.14 (0.07-0.22)**	**<0.001**

Abbreviations: ART, antiretroviral therapy; PTB, pulmonary tuberculosis; EPTB, extrapulmonary tuberculosis; KS, Kaposi sarcoma; IQR, interquartile range; 95% CI, 95% confidence interval

For peer review only - http://bmjopen.bmj.com/site/about/guidelines.xhtml

Table 2: Regression coefficients between deviations of monthly counts of pulmonary tuberculosis, extrapulmonary tuberculosis, or Kaposi Sarcoma diagnoses and deviations of monthly counts of antiretroviral therapy enrolments from the yearly averages (unadjusted); and adjusted for the deviations of monthly temperatures to yearly average temperatures (adjusted). Monthly air temperatures are based on 1961-1990 averages.

ART program	PTB diagnoses		EPTB diagnoses		KS diagnoses	
	Coefficient (95% CI)	p-value	Coefficient (95% CI)	p-value	Coefficient (95% CI)	p-value
**South Africa – Khayelitsha**						
Unadjusted	0.33 (0.13-0.53)	0.002	0.28 (0.05-0.51)	0.019	0.30 (-0.43-1.03)	0.416
Adjusted	0.30 (0.09-0.51)	0.005	0.27 (0.03-0.51)	0.027	0.44 (-0.31-1.12)	0.248
**South Africa – Themba Lethu**						
Unadjusted	0.16 (0.04-0.28)	0.010	0.21 (-0.04-0.45)	0.097	0.07 (-0.74-0.88)	0.860
Adjusted	0.17 (0.05-0.29)	0.005	0.21 (-0.03-0.46)	0.089	0.10 (-0.71-0.92)	0.805
**South Africa – Tygerberg**						
Unadjusted	0.41 (0.04-0.78)	0.028	0.20 (-0.20-0.60)	0.328	0.82 (-0.49-2.14)	0.218
Adjusted	0.41 (0.45-0.78)	0.028	0.21 (-0.20-0.62)	0.307	0.92 (-0.39-2.23)	0.169
**Zambia – CIDRZ**						
Unadjusted	0.84 (0.74-0.95)	<0.001	1.39 (1.00-1.78)	<0.001	0.91 (0.73-1.08)	<0.001
Adjusted	0.83 (0.72-0.94)	<0.001	1.40 (1.00-1.80)	<0.001	0.90 (0.72-1.08)	<0.001
**Zimbabwe – Newlands**						
Unadjusted	0.29 (0.03-0.54)	0.029	0.47 (0.11-0.83)	0.011	1.32 (0.27-2.38)	0.014
Adjusted	0.29 (0.037-0.55)	0.025	0.47 (0.12-0.84)	0.010	1.31 (0.26-2.36)	0.015
**All**						
Unadjusted	0.37 (0.27-0.47)	<0.001	0.50 (0.35-0.65)	<0.001	0.75 (0.35-1.16)	<0.001
Adjusted	0.38 (0.28-0.48)	<0.001	0.51 (0.36-0.66)	<0.001	0.77 (0.36-1.18)	<0.001

PTB, pulmonary tuberculosis; EPTB, extrapulmonary tuberculosis; KS, Kaposi sarcoma; 95% CI, 95% confidence interval.

For peer review only

For peer review only - http://bmjopen.bmj.com/site/about/guidelines.xhtml

Supplemental Digital Content

SDC Figure 1: Selection of patients for the analysis.

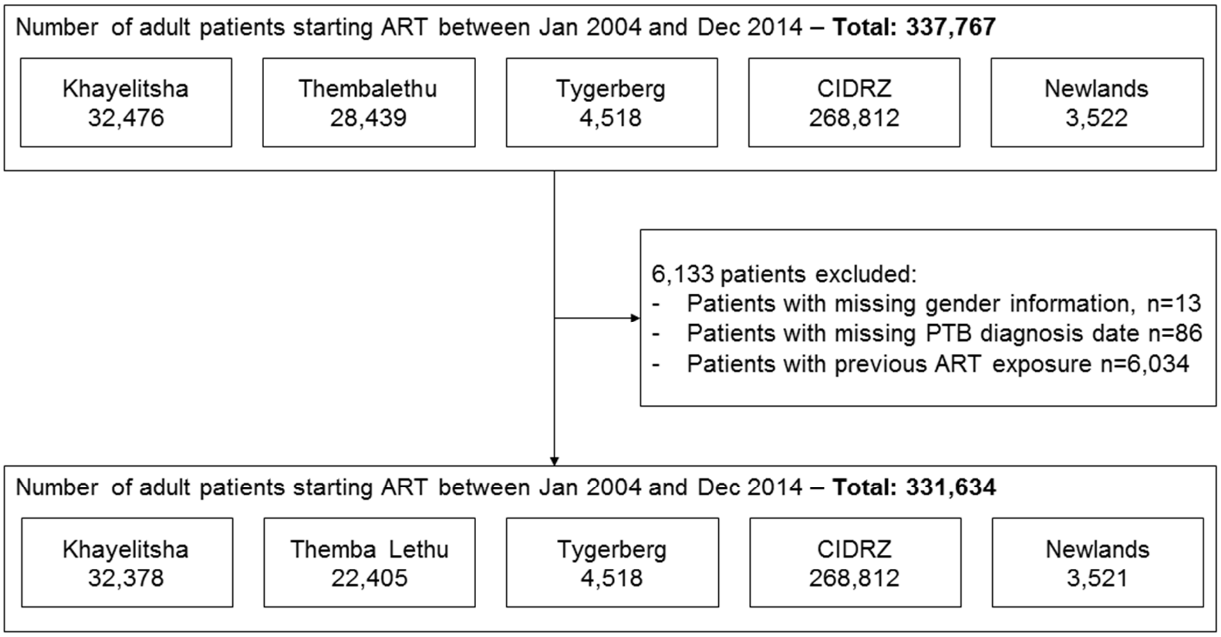

ART, antiretroviral therapy; PTB, pulmonary tuberculosis

For peer review only - http://bmjopen.bmj.com/site/about/guidelines.xhtml

SDC Figure 2: Quarterly counts of patients newly started on antiretroviral therapy, and quarterly counts of pulmonary tuberculosis diagnoses between 2004 and 2014.

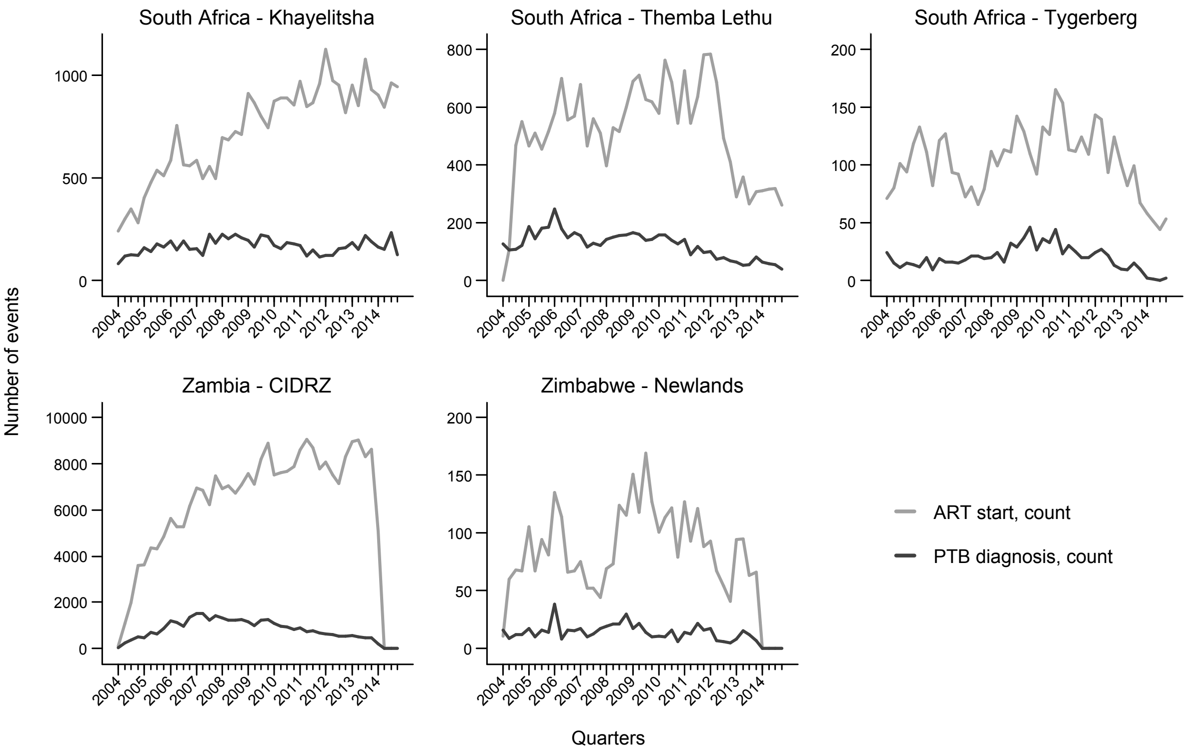

ART, antiretroviral therapy; PTB, pulmonary tuberculosis

w only

For peer review only - http://bmjopen.bmj.com/site/about/guidelines.xhtml

SDC Figure 3: Mean deviations of monthly counts in antiretroviral therapy initiations and pulmonary tuberculosis diagnosis from the corresponding yearly averages.

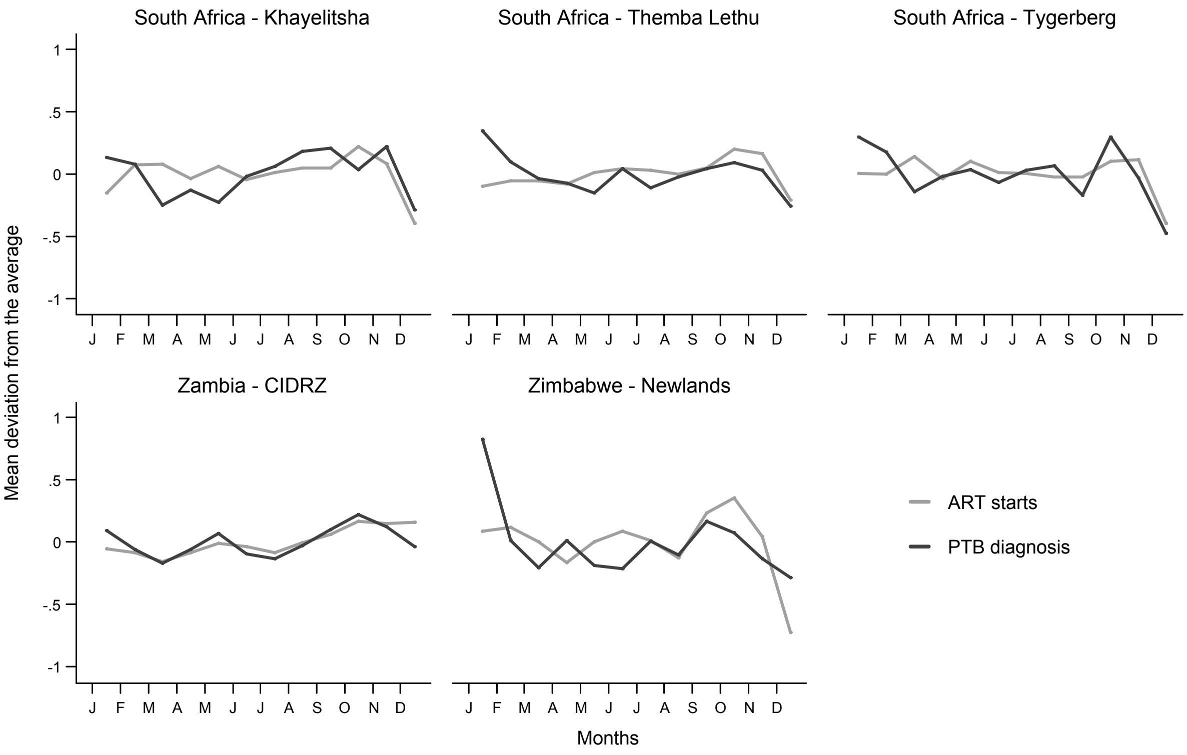

ART, antiretroviral therapy; PTB, pulmonary tuberculosis

w only

For peer review only - http://bmjopen.bmj.com/site/about/guidelines.xhtml

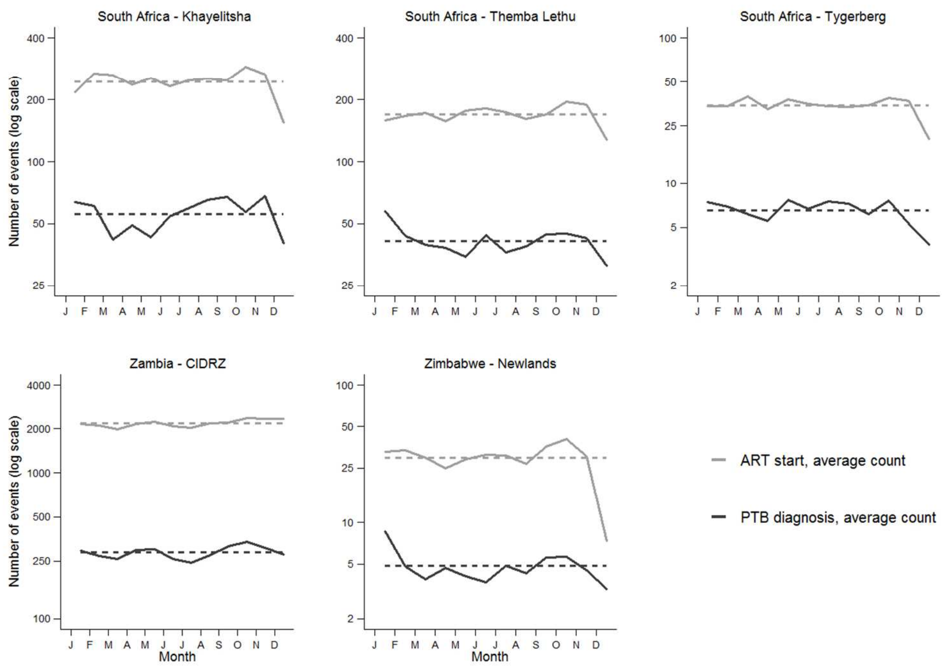

Figure 1: Aggregated averages in monthly counts of patients newly started on antiretroviral therapy and monthly counts of pulmonary tuberculosis diagnosis (aggregate averages 2004-2014, logarithmic scale). Dashed horizontal lines indicate overall monthly means.

473x344mm (72 x 72 DPI)

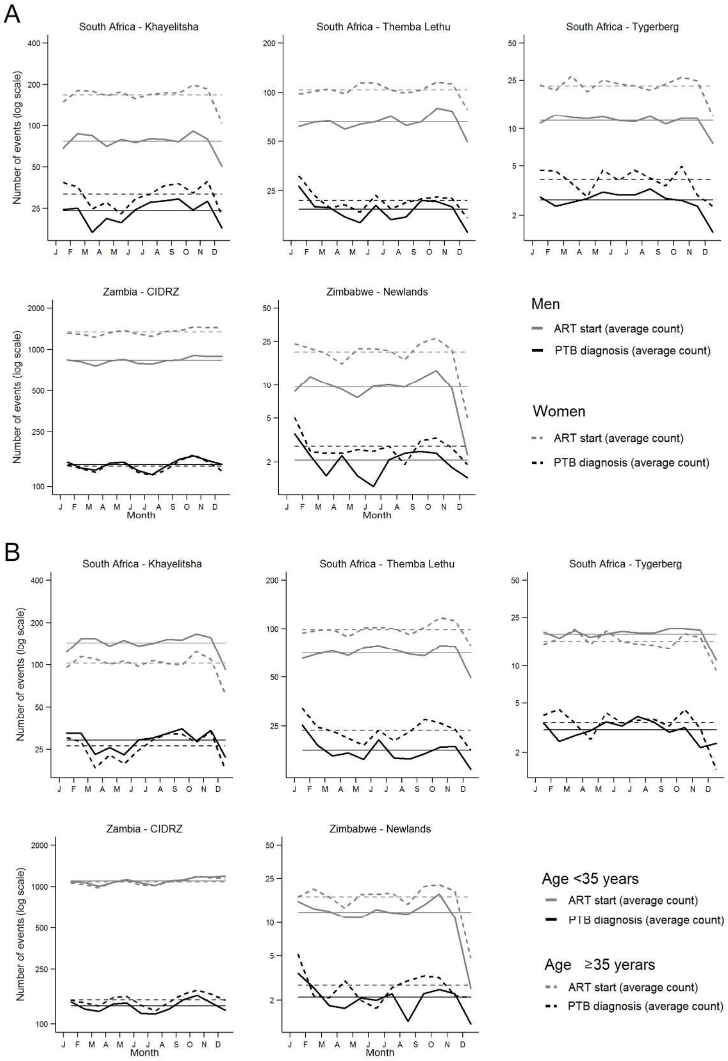

Figure 2: Aggregated averages in monthly counts of patients newly started on antiretroviral therapy and monthly counts of pulmonary tuberculosis diagnosis (study period 2004-2014), by gender (panel A) and by age groups (panel B). Horizontal lines indicate the overall monthly mean of ART enrolment events or PTB diagnosis events.

199x314mm (300 x 300 DPI)

Supplemental Digital Content

Seasonal variations in tuberculosis diagnosis at antiretroviral treatment programs in Southern Africa

SDC Figure 1: Selection of patients for the analysis.

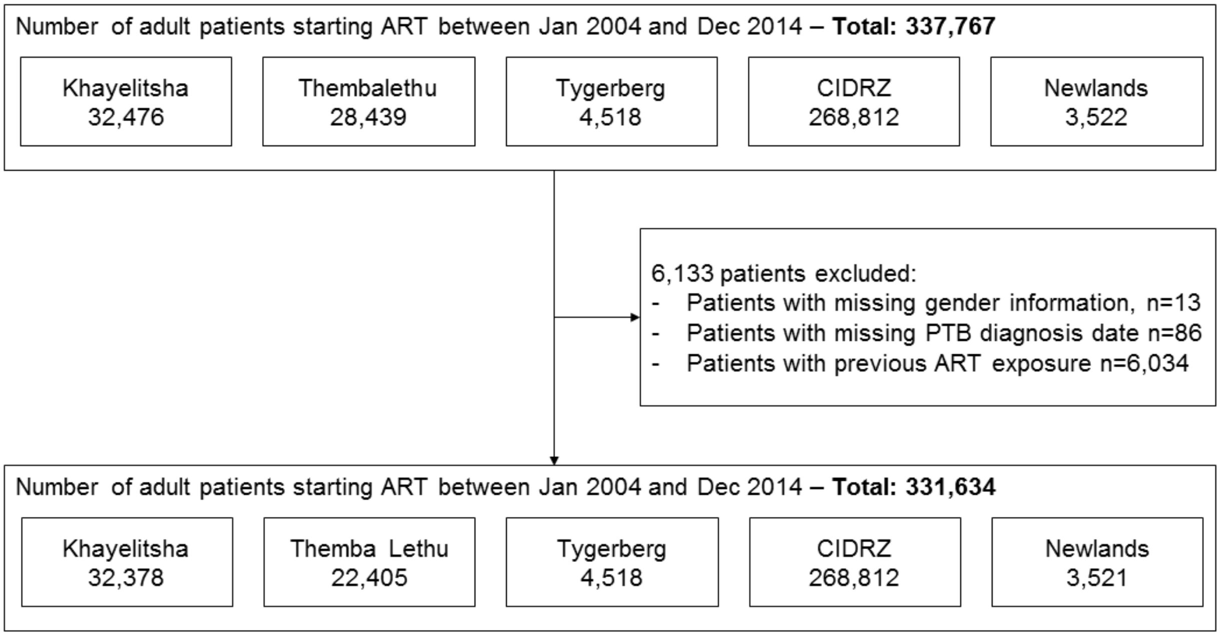

ART, antiretroviral therapy; PTB, pulmonary tuberculosis

For peer review only - http://bmjopen.bmj.com/site/about/guidelines.xhtml

SDC Figure 2: Quarterly counts of patients newly started on antiretroviral therapy, and quarterly counts of pulmonary tuberculosis diagnoses between 2004 and 2014.

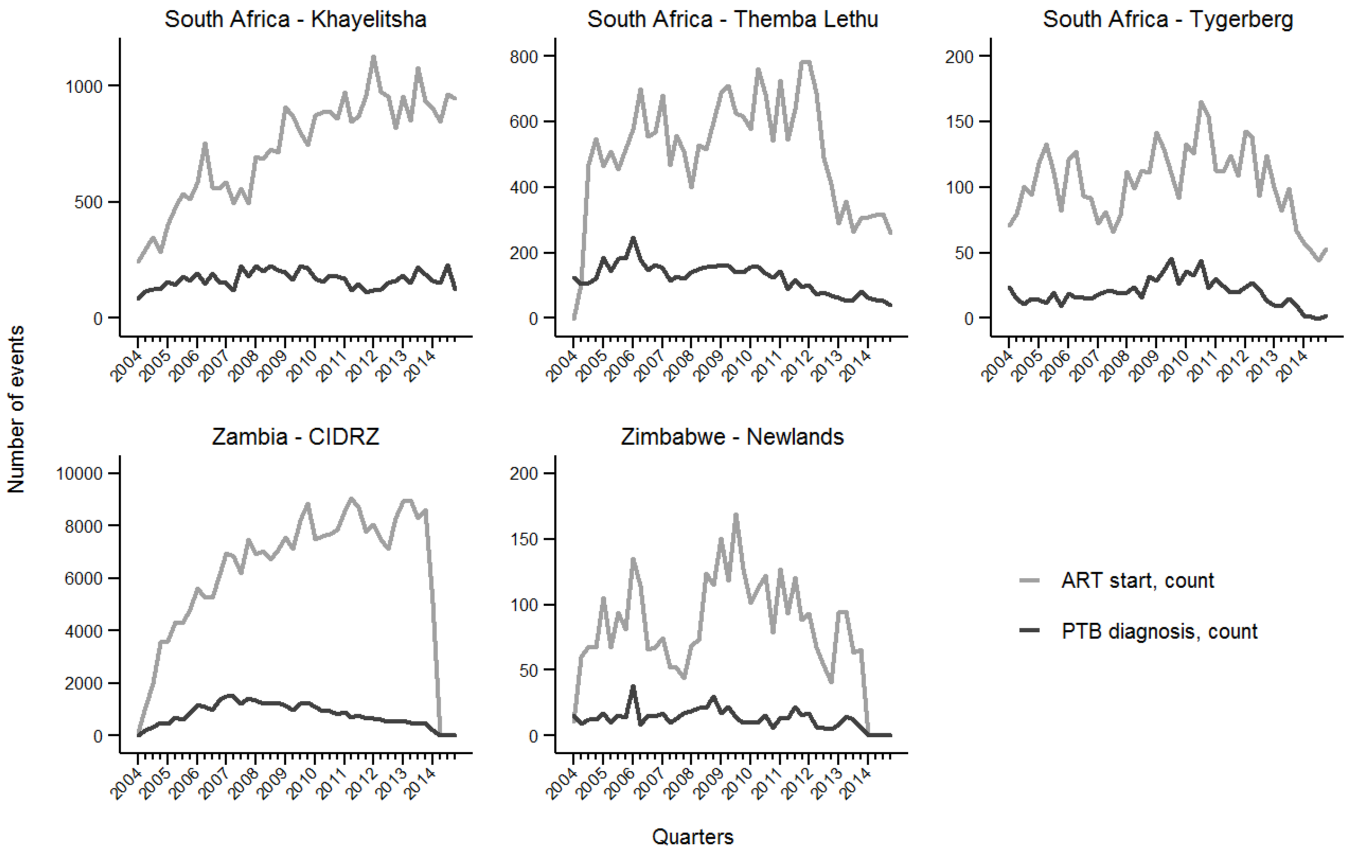

ART, antiretroviral therapy; PTB, pulmonary tuberculosis

only

For peer review only - http://bmjopen.bmj.com/site/about/guidelines.xhtml

SDC Figure 3: Mean deviations of monthly counts in antiretroviral therapy initiations and pulmonary tuberculosis diagnosis from the corresponding yearly averages.

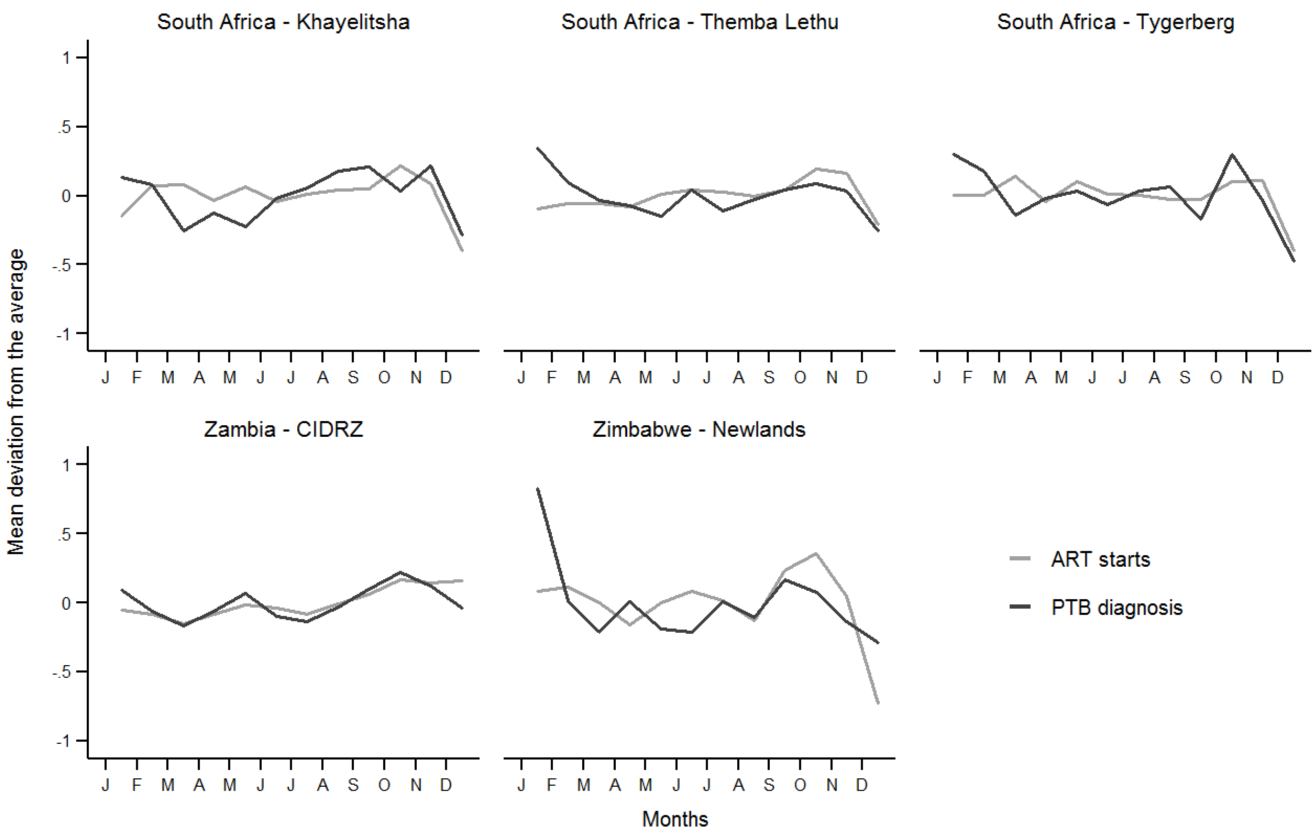

ART, antiretroviral therapy; PTB, pulmonary tuberculosis

For peer review only - http://bmjopen.bmj.com/site/about/guidelines.xhtml

STROBE Statement—Checklist of items that should be included in reports of ***cohort studies***

Manuscript: Seasonal variations in tuberculosis diagnosis among HIV-coinfected individuals in Southern Africa: an observational cohort study at antiretroviral treatment programs

	Item No	Recommendation	Manuscript page number
**Title and abstract**	1	(*a*) Indicate the study's design with a commonly used term in the title or the abstract	1
		(*b*) Provide in the abstract an informative and balanced summary of what was done and what was found	3
**Introduction**			
Background/rationale	2	Explain the scientific background and rationale for the investigation being reported	5
Objectives	3	State specific objectives, including any prespecified hypotheses	5
**Methods**			
Study design	4	Present key elements of study design early in the paper	6-7
Setting	5	Describe the setting, locations, and relevant dates, including periods of recruitment, exposure, follow-up, and data collection	6
Participants	6	(*a*) Give the eligibility criteria, and the sources and methods of selection of participants. Describe methods of follow-up	6
		(*b*) For matched studies, give matching criteria and number of exposed and unexposed	NA
Variables	7	Clearly define all outcomes, exposures, predictors, potential confounders, and effect modifiers. Give diagnostic criteria, if applicable	6
Data sources/ measurement	8*	For each variable of interest, give sources of data and details of methods of assessment (measurement). Describe comparability of assessment methods if there is more than one group	6
Bias	9	Describe any efforts to address potential sources of bias	6
Study size	10	Explain how the study size was arrived at	NA
Quantitative variables	11	Explain how quantitative variables were handled in the analyses. If applicable, describe which groupings were chosen and why	
Statistical methods	12	(*a*) Describe all statistical methods, including those used to control for confounding	6-7
		(*b*) Describe any methods used to examine subgroups and interactions	6-7
		(*c*) Explain how missing data were addressed	6-7
		(*d*) If applicable, explain how loss to follow-up was addressed	NA
		(*e*) Describe any sensitivity analyses	NA
**Results**			
Participants	13*	(a) Report numbers of individuals at each stage of study—eg numbers potentially eligible, examined for eligibility, confirmed eligible, included in the study, completing follow-up, and analysed	8 + SCD Fig. 1
		(b) Give reasons for non-participation at each stage	SCD Fig. 1
		(c) Consider use of a flow diagram	SCD Fig. 1
Descriptive data	14*	(a) Give characteristics of study participants (eg demographic, clinical, social) and information on exposures and potential	8 + Fig. 1

For peer review only - http://bmjopen.bmj.com/site/about/guidelines.xhtml

		confounders	
		(b) Indicate number of participants with missing data for each variable of interest	**Table 1 + SCD Fig. 1**
		(c) Summarise follow-up time (eg, average and total amount)	**8**
Outcome data	15*	Report numbers of outcome events or summary measures over time	**8-9**
Main results	16	(*a*) Give unadjusted estimates and, if applicable, confounder-adjusted estimates and their precision (eg, 95% confidence interval). Make clear which confounders were adjusted for and why they were included	**8-9**
		(*b*) Report category boundaries when continuous variables were categorized	**NA**
		(*c*) If relevant, consider translating estimates of relative risk into absolute risk for a meaningful time period	**NA**
Other analyses	17	Report other analyses done—eg analyses of subgroups and interactions, and sensitivity analyses	**8-9**
**Discussion**			
Key results	18	Summarise key results with reference to study objectives	**10**
Limitations	19	Discuss limitations of the study, taking into account sources of potential bias or imprecision. Discuss both direction and magnitude of any potential bias	**11-12**
Interpretation	20	Give a cautious overall interpretation of results considering objectives, limitations, multiplicity of analyses, results from similar studies, and other relevant evidence	**10-12**
Generalisability	21	Discuss the generalisability (external validity) of the study results	**11-12**
**Other information**			
Funding	22	Give the source of funding and the role of the funders for the present study and, if applicable, for the original study on which the present article is based	**13**

*Give information separately for exposed and unexposed groups.

Note: An Explanation and Elaboration article discusses each checklist item and gives methodological background and published examples of transparent reporting. The STROBE checklist is best used in conjunction with this article (freely available on the Web sites of PLoS Medicine at http://www.plosmedicine.org/, Annals of Internal Medicine at http://www.annals.org/, and Epidemiology at http://www.epidem.com/). Information on the STROBE Initiative is available at http://www.strobe-statement.org.

For peer review only

For peer review only - http://bmjopen.bmj.com/site/about/guidelines.xhtml

BMJ Open

Seasonal variations in tuberculosis diagnosis among HIV-positive individuals in Southern Africa: analysis of cohort studies at antiretroviral treatment programs

Journal:	*BMJ Open*
Manuscript ID	bmjopen-2017-017405.R1
Article Type:	Research
Date Submitted by the Author:	23-Aug-2017
Complete List of Authors:	Ballif, Marie; University of Bern, Insitute of Social and Preventive Medicine<br>Zürcher, Kathrin; University of Bern, Insitute of Social and Preventive Medicine<br>Reid, Stewart; University of Alabama at Birmingham; Centre for Infectious Disease Research in Zambia<br>Boulle, Andrew; University of Cape Town; Médecins Sans Frontières<br>Fox, Matt; Boston University, Epidemiology and Global Health; University of the Witwatersrand<br>Prozesky, Hans; Division of Infectious Diseases, Department of Medicine, University of Stellenbosch & Tygerberg Academic Hospital<br>Chimbetete, Cleophas; Newlands Clinic<br>Zwahlen , Marcel; University of Bern, Insitute of Social and Preventive Medicine<br>Egger, Matthias; University of Bern, Institute of Social and Preventive Medicine; School of Public Health and Family Medicine, University of Cape Town<br>Fenner, Lukas; University of Bern, Institute of Social and Preventive Medicine
**Primary Subject Heading**:	HIV/AIDS
Secondary Subject Heading:	Infectious diseases, Epidemiology
Keywords:	Seasonality, pulmonary tuberculosis, HIV & AIDS < INFECTIOUS DISEASES, South Africa, Zambia, Zimbabwe

SCHOLARONE™ Manuscripts

TITLE PAGE

Seasonal variations in tuberculosis diagnosis among HIV-positive individuals in Southern Africa: analysis of cohort studies at antiretroviral treatment programs

Marie Ballif [1 §], Kathrin Zürcher [1], Stewart E. Reid [2 3], Andrew Boulle [4 5], Matthew P. Fox [6 7], Hans W. Prozesky [8], Cleophas Chimbetete [9], Marcel Zwahlen [1], Matthias Egger [1 4], Lukas Fenner [1]; for the International Epidemiology Database to Evaluate AIDS in Southern Africa (IeDEA-SA)

1 Institute of Social and Preventive Medicine, University of Bern, Bern, Switzerland

2 University of Alabama at Birmingham, Birmingham, USA

3 Centre for Infectious Disease Research in Zambia (CIDRZ), Lusaka, Zambia

4 Centre for Infectious Disease Epidemiology and Research (CIDER), School of Public Health and Family Medicine, University of Cape Town, Cape Town, South Africa

5 Médecins Sans Frontières, Khayelitsha, Cape Town, South Africa

6 Health Economics and Epidemiology Research Office, Department of Internal Medicine, School of Clinical Medicine, Faculty of Health Sciences, University of the Witwatersrand, Johannesburg, South Africa

7 Departments of Epidemiology and Global Health, Boston University, Boston, USA

8 Division of Infectious Diseases, Department of Medicine, University of Stellenbosch and Tygerberg Hospital, Cape Town, South Africa

9 Newlands Clinic, Harare, Zimbabwe

For peer review only

For peer review only - http://bmjopen.bmj.com/site/about/guidelines.xhtml

§ **Corresponding author:**

Dr Marie Ballif

Institute of Social and Preventive Medicine, University of Bern

Finkenhubelweg 11, 3012 Bern, Switzerland

Email: marie.ballif@ispm.unibe.ch

Phone: +41 31 631 35 18 Fax: +41 31 631 35 20

Keywords:

Seasonality, pulmonary tuberculosis, antiretroviral therapy, HIV, South Africa, Zambia, Zimbabwe

Word count:

Main text 2350 (max. 4000), abstract 300 (max. 300), references: 41

Inserts:

4 tables and 3 figures, Supplementary File (3 figures)

For peer review only

Abstract

Objectives Seasonal variations in tuberculosis diagnoses have been attributed to seasonal climatic changes and indoor crowding during colder winter months. We investigated trends in pulmonary tuberculosis (PTB) diagnosis at antiretroviral therapy (ART) programs in Southern Africa.

Setting Five ART programs participating in the International Epidemiology Database to Evaluate AIDS in South Africa, Zambia, and Zimbabwe.

Participants We analyzed data of 331,634 HIV-positive adults (>15 years), who initiated ART between January 2004 and December 2014.

Primary outcome measure We calculated aggregated averages in monthly counts of PTB diagnoses and ART initiations. To account for time trends, we compared deviations of monthly event counts to yearly averages, and calculated correlation coefficients. We used multivariable regressions to assess associations between deviations of monthly ART initiation and PTB diagnosis counts from yearly averages, adjusted for monthly air temperatures and geographical latitude. As controls, we used Kaposi sarcoma and extrapulmonary tuberculosis diagnoses.

Results All programs showed monthly variations in PTB diagnoses that paralleled fluctuations in ART initiations, with recurrent patterns across 2004-2014. The strongest drops in PTB diagnoses occurred in December, followed by April-May in Zimbabwe and South Africa. This corresponded to holiday seasons, when clinical activities are reduced. We observed little monthly variation in ART initiations and PTB diagnoses in Zambia. Correlation coefficients supported parallel trends in ART initiations and PTB diagnoses (correlation coefficient: 0.28, 95% confidence interval: 0.21-0.35, p-value <0.001). Monthly temperatures and latitude did not substantially change regression coefficients between ART initiations and PTB diagnoses. Trends in Kaposi sarcoma and extrapulmonary tuberculosis diagnoses similarly followed changes in ART initiations throughout the year.

Conclusions Monthly variations in PTB diagnosis at ART programs in Southern Africa likely occurred regardless of seasonal variations in temperatures or latitude, and reflected

For peer review only - http://bmjopen.bmj.com/site/about/guidelines.xhtml

For peer review only

fluctuations in clinical activities and changes in health-seeking behaviour throughout the year, rather than climatic factors.

Strengths and limitations of this study

- We analyzed a large dataset from HIV-positive patients routinely collected over 10 years at ART programs in low- and middle-income countries from the Southern African region;
- Included ART programs represent a wide range of countries with different climatic seasons according to their geographical latitude;
- We compared trends in PTB diagnosis to trends in Kaposi sarcoma and extrapulmonary tuberculosis diagnoses, for which no effect due to seasonal climatic changes was expected;
- Our study is limited by the lack of social and environmental information on participants (socioeconomic factors, housing conditions);
- Trends in tuberculosis diagnosis and other opportunistic infections in HIV-positive individuals may differ from trends in HIV-negative populations.

For peer review only - http://bmjopen.bmj.com/site/about/guidelines.xhtml

Introduction

Countries in different regions of the world have reported seasonal fluctuations in tuberculosis (TB) cases [1–6]. In temperate climate zones, peaks in TB diagnosis are generally observed in the spring, following the colder times of the year. The reasons for these variations are multiple, with both social and environmental factors likely playing a role [7]. Peaks in TB diagnosis following winter time have been associated with several factors: more time spent indoor in poorly ventilated rooms, which increases the risk of TB transmission; poorer access to health care when rough weather conditions make transport difficult; lower vitamin D levels due to reduced sunlight exposure, which impairs adequate immune response against infections [1,3,5,8–10].

In 2015, an estimated 1.2 million of people living with HIV developed TB, and one third of them died from TB, making TB the leading cause of death in this population [11–13]. However, little is known about the seasonal variation of TB diagnoses among HIV-positive individuals in care at antiretroviral therapy (ART) programs in sub-Saharan Africa, where the burden of HIV/TB coinfection is the highest [13]. We hypothesized that fluctuations in TB diagnoses throughout the year would likely be stronger in countries located farther away from the equator and that experience more pronounced climatic changes, as opposed to countries located closer to the equator. We investigated seasonal trends in pulmonary TB (PTB) diagnosis at five ART programs participating in the International Epidemiology Databases to Evaluate AIDS collaboration in Southern Africa (IeDEA-SA). These ART programs are located at different geographical latitudes in South Africa, Zambia, and Zimbabwe and present differently marked climatic seasons.

For peer review only - http://bmjopen.bmj.com/site/about/guidelines.xhtml

Methods

We included all HIV-positive persons age 16 and older who enrolled between January 1, 2004 and December 31, 2014 in five ART programs that participate in the IeDEA-SA collaboration (www.iedea-sa.org) and systematically collect information on opportunistic infections (OIs) [14]: Khayelitsha ART program, Themba Lethu clinic, and Tygerberg Academic Hospital in South Africa; Centre for Infectious Disease Research in Zambia (CIDRZ); and Newlands Clinic in Zimbabwe. IeDEA-SA sites provide data collected as part of routine HIV care, including TB diagnosis, at enrolment in the ART program and at each follow-up visit. Site characteristics are shown in Table 1 [15], the patient selection in Figure 1.

All study sites have approval from a local institutional review board or ethics committee to collect data and participate in IeDEA-SA projects. In addition, this project was approved by the Cantonal Ethics Committee Bern (swissethics, Bern, Switzerland) and the University of Cape Town Human Research Ethics Committee (Cape Town, South Africa).

PTB was defined according to the case definition in use at the participating IeDEA-SA sites (Table 1) [15]. We restricted the main analysis to PTB, because the diagnosis of PTB has been associated with seasonal climatic variations [1,2,7]. For comparison, we analysed monthly counts of patients diagnosed with extrapulmonary TB (EPTB) and the AIDS-defining cancer Kaposi sarcoma (KS), which are not expected to be seasonal. One record per patient was analysed. If a patient had several episodes of the same disease (PTB, EPTB, or KS), we only considered the first event of each. If a patient was diagnosed with multiple diseases of interest, priority was given to the record with PTB diagnosis, followed by EPTB and KS. We obtained mean monthly air temperatures (°C) based on 1961–1990 averages at each location [16–19].

For each site, we calculated aggregated averages as the average number of events per calendar month for 2004-2014. We accounted for calendar time trends over the study period by comparing deviations of monthly event counts to yearly averages. Site-specific aggregated averages and monthly deviations were calculated for ART enrolments and for PTB, EPTB, and KS diagnoses, overall or stratified by gender and age. We assessed the association between monthly deviations in the number of ART enrolments and the number of PTB diagnoses by

For peer review only - http://bmjopen.bmj.com/site/about/guidelines.xhtml

calculating pairwise Pearson's correlation coefficients [20]. We used Fisher’s transformation to calculate 95% confidence intervals (CI) for the correlation coefficient. Finally, we used linear regression models to assess associations between the monthly deviations of PTB, EPTB or KS diagnosis counts and the monthly deviations of ART initiation counts, unadjusted and adjusted for monthly deviations of air temperatures from yearly averages and for the geographical latitude of the ART programs’ locations.

All analyses were performed in Stata version 14.1 (Stata Corporation, College Station, TX, USA).

For peer review only

For peer review only - http://bmjopen.bmj.com/site/about/guidelines.xhtml

Results

We analysed data of 331,634 HIV-positive adults (Figure 1). Of these 49,367 (14.9%) were diagnosed with PTB. Median follow-up time was 2.0 years (interquartile range [IQR], 0.7-4.2), patients' median age was 34 years (IQR, 29-41), and 207,074 (62.4%) were women (Table 2). Median CD4 cell count at ART initiation of these patients was 116 cells/mm^3 (IQR: 52-200); this compares to a median cell count of 166 (87-254) in patients who were never diagnosed with PTB.

The mean number of PTB diagnoses per month was 55.7 (range 40.2-67.8) in Khayelitsha, 41.2 (31.1-57.6) in Themba Lethu, 6.5 (3.8-7.7) in Tygerberg, 285.6 (243.2-338.2) in CIDRZ, and 4.8 (3.3-8.7) in Newlands (Figure 2). At each site, monthly variations in numbers of PTB diagnoses were parallel to trends in ART enrolments. Aggregated averages over the study period showed that monthly fluctuations in PTB diagnoses were present at all sites (Figure 2). The strongest fluctuations were seen in Zimbabwe (Newlands), with PTB diagnosis peaks in January and troughs in December (+87% and -33% compared to the monthly average, respectively). Similar to Zimbabwe, all three South African sites showed marked drops in ART initiations and PTB diagnoses in December, in addition to a milder drop in April-May. In contrast, Zambia (CIDRZ) showed little variation in ART enrolments and PTB diagnoses throughout the year. We consistently observed a similar seasonal pattern in patients newly starting ART and PTB diagnoses every year (Supplementary Figure 1), in men and women, as well as in younger and older patients (Supplementary Figure 2).

The deviations of monthly event counts from the yearly averages confirmed the congruent fluctuations of ART initiation and PTB diagnosis (Supplementary Figure 3). This was further supported by the correlation coefficients between the deviations of monthly ART initiations from yearly averages and the deviations of monthly PTB diagnoses from yearly averages (Table 3): coefficient was 0.27 in Khayelitsha (95% confidence interval [CI] 0.11-0.42, p=0.002), 0.22 in Themba Lethu (95% CI 0.05-0.38, p=0.010), 0.19 in Tygerberg (95% CI 0.02-0.35, p=0.028), 0.82 in CIDRZ (95% CI 0.75-0.87, p-value<0.001), and 0.20 in Newlands (95% CI 0.02-0.37, p=0.029). Regression coefficients between deviations of monthly PTB diagnosis counts and monthly ART initiation counts from yearly averages did not substantially change

For peer review only - http://bmjopen.bmj.com/site/about/guidelines.xhtml

when adjusted for monthly temperatures and latitude (Table 4). This was further supported by Figure 3, which showed no evidence for a relationship between the adjusted regression coefficients and the latitude of the ART program's location (r=0.43, p=0.47).

To determine whether the diagnosis of other OIs followed trends similar to that of PTB and ART initiations, we studied the monthly diagnoses of EPTB and KS, which we did not expect to be influenced by climatic factors. The seasonal pattern of EPTB diagnoses paralleled that of ART initiation at most sites despite low numbers of EPTB diagnoses (1.8 to 26.5 mean monthly EPTB diagnoses). The deviations of monthly ART initiations from the yearly averages correlated with those of EPTB diagnoses in Khayelitsha, CIDRZ, and Newlands (correlation coefficient range: 0.20-0.54, Table 3). Although KS was a rare diagnosis averaging 1.1 cases per month overall, we observed that the fluctuations in KS diagnosis counts similarly followed changes in ART enrolment at all sites (Table 3). As for PTB diagnoses, the adjusted regression models did not support any influence of temperature or latitude on the observed fluctuations in EPTB or KS diagnoses (Table 4).

For peer review only

Discussion

We observed monthly variations in PTB diagnosis at ART programs in South Africa, Zambia and Zimbabwe. These fluctuations occurred regardless of latitudes and followed trends in ART enrolment rather than seasonal variations in temperatures, hence mirroring fluctuations in clinical activity. In South Africa and Zimbabwe, a marked drop of ART enrolments and PTB diagnoses was seen in December, followed by a rebound in January-February. A milder drop was also observed in South African sites around April-May.

In Southern Africa, seasons are marked by cooler winters and warmer summers in Cape Town (Khayelitsha, Tygerberg), and become less pronounced as latitude decreases, in Johannesburg (Themba Lethu), Zimbabwe and Zambia. Therefore, we expected to see stronger fluctuations in PTB diagnosis in the Western Cape (South Africa) than in Zambia and Zimbabwe, with peaks following winter. Instead, we observed variations in PTB that seemed independent from climatic changes. This might be explained by mild winters in Southern Africa, contrasting with countries with harsh winters. Mongolia typically has cold winters and strong seasonal patterns in TB notifications, likely due to increased airborne transmission from infectious PTB individuals resulting from indoor crowding and poorer ventilation during winter [1,2]. Nevertheless, seasonal variations in TB notifications in a population-based study of HIV-positive and HIV-negative individuals were documented in Cape Town, South Africa [8].

We observed that variations in PTB diagnoses followed fluctuations in ART initiations and hence likely reflected activities at the clinic, rather than any climatic effects. This was supported by an association between changes in ART initiation and PTB diagnosis counts (unadjusted and adjusted for monthly climatic temperatures and latitude). Furthermore, important drops in ART initiations and PTB diagnoses were observed every year in December in South Africa and Zimbabwe, when clinical activities are reduced due to the holiday season. December drops were followed by peaks in January and February, when clinical activity resumed. Similar declines in PTB diagnoses at the end of the year have been reported in Zimbabwe and Uganda by Mabaera et al. [2]. In CIDRZ, the largest of the cohorts, which we studied, monthly fluctuations in ART initiations and PTB diagnoses were the least marked, as compared to the South African and Zimbabwean sites. This can be explained by the shorter

For peer review only

December holiday period in the Zambian health sector, in particular in the urban and peri-urban region of Lusaka, where CIDRZ is based. There, the ART programs continue to run over the festive season in December and are only closing on official public holidays. Furthermore, HIV-positive individuals seeking care at CIDRZ are mainly permanent residents in Lusaka city (as opposed to migrants from rural areas), and hence less likely to leave the city to visit their family in other parts of the country during holidays, which would contribute to delayed TB diagnoses. Similarly, Easter vacations likely explain the milder drop observed in April-May in South Africa, and to a lesser extend in Zimbabwe, but not in Zambia. Age and gender did not seem to influence the seasonal variations.

EPTB and KS diagnoses also followed variations in ART initiations in our study. We did not expect the development of EPTB to be strongly marked by climatic changes. Indeed, EPTB is less likely resulting from recent transmission than PTB, the transmission mode of which is airborne, and hence more likely to be influenced by indoor crowding and reduced ventilation during winter [1,7]. Seasonal changes in EPTB case detection have been reported [3,4,21]. However, the underlying reasons for EPTB seasonality may considerably differ from those involved in PTB. In addition, EPTB is often underdiagnosed due to lack of adequate laboratory diagnostic tools in underresourced settings [3,21]. Similarly to EPTB, KS was not expected to be influenced by climatic factors. The observed seasonal fluctuations of EPTB and KS diagnoses hence reflect variations in clinical activities, such as observed for PTB.

Several explanations of why PTB diagnoses may vary seasonally have been offered [1,3,5,7–9]. Beyond indoor crowding [1,5,9] and reduced clinical activities at specific times during the year [2], health-seeking behaviour is also influencing access to care. Health-seeking behaviour might reflect environmental barriers (long distances to the clinic, waiting time) [22,23], psychological barriers (stigma) [24], and/or clinical barriers (poor patient's health status and disabilities) [25]. Access to clinics can be problematic when temperatures are low and weather conditions are bad. Under these circumstances, PTB diagnosis might be delayed to spring. Professional activities or beliefs may also prevent early healthcare seeking. Economical aspects play an important role, where care is to be fully or partly paid by patients [15]. These aspects might differently affect individuals, depending on gender or age [26–29]. However, in

For peer review only - http://bmjopen.bmj.com/site/about/guidelines.xhtml

our study populations, trends in ART initiations and PTB diagnoses were independent from gender or age. Finally, the role of vitamin D deficiency has been debated, and studies of the relationship between vitamin D deficiency during winter and PTB are inconsistent [3,30–32].

Our study is limited by the possible under-ascertainment of TB diagnoses at ART programs, and by the fact that we could only include IeDEA-SA sites that routinely collect information on OIs. However, the included ART programs cover a wide range of geographical latitudes, with Khayelitsha and Tygerberg at the southern tip and CIDRZ at the most equatorial end of the Southern African region. The findings of our study is restricted to HIV-positive populations, since trends in TB diagnosis and other OIs at ART programs may differ from trends in HIV-negative populations. Due the high early TB mortality, HIV-positive individuals may not reach clinical care for a TB diagnosis [33]. In addition, although ART reduces the risk of OIs significantly people living with HIV remain at a higher risk of developing TB [34,35]. Finally, social and environmental factors, including housing conditions, distance to health care facilities, access to care, disease awareness, and health-seeking behaviour may differently affect the timely diagnosis of TB in HIV-positive compared to HIV-negative populations [1,7,9,36,37]. Unfortunately, this information is not routinely collected at ART programs in the IeDEA-SA collaboration.

Conclusions

In conclusion, our study suggests that yearly trends in PTB diagnosis at ART programs in Southern Africa are mainly driven by seasonal variations in clinical activities, access to care and health-seeking behaviour, rather than climatic seasons. Our results underline the importance of offering regular care for HIV-infected individuals, including screenings for OIs such as TB at ART initiation and during follow-up visits, as part of integrated TB/HIV clinical activities at ART programs [38–41].

For peer review only - http://bmjopen.bmj.com/site/about/guidelines.xhtml

Funding

This work was supported by the National Institute of Allergy and Infectious Diseases (NIAID), the National Institute of Child Health and Human Development (NICHD), and the National Cancer Institute (NCI) under award number U01AI069924. MPF was supported by USAID 674-A-12-00029 from the United States Agency for International Development. The opinions expressed herein are those of the authors and do not necessarily reflect the views of the funder. The funder had no role in study design, data collection and analysis, decision to publish, or preparation of the manuscript.

Competing interests

The authors declare no conflicts of interest directly related to this project. MZ discloses the following unrelated funding sources: MZ has received grant support from the Swiss National Science Foundation and from the Swiss Cancer league which paid for PhD and postdoc positions. Furthermore, MZ has consulted for BIOTRONIK AG, Switzerland, and has projects which received unrestricted research support from following firms: AstraZeneca AG, Switzerland, Aptalis Pharma Inc., Dr. Falk Pharma GmbH, Germany, Glaxo Smith Kline AG, Nestlé S. A., Switzerland, Receptos Inc., Regeneron Inc. For the remaining authors none were declared.

Data sharing Statement

The individual contributing cohorts own the data. Consortium members may request data for analysis according to standard procedures. The consortium can also accept concept proposals from qualified researchers external to the collaboration. For more information, see www.iedea.org/working-groups/executive-committee.

Authors' contributions

MB analysed the data and completed the final draft of the manuscript. LF conceptualized the project. MZ established the methodology. KZ, SER, AB, MPF, HWP, CC and ME provided

For peer review only

For peer review only - http://bmjopen.bmj.com/site/about/guidelines.xhtml

input into the study design, analyses, and drafting of the paper. All authors reviewed and approved the final version of the manuscript.

Acknowledgements

We thank all participating sites and patients whose data were used in this study. We also would like to thank collaborators who contributed to recording and entering data, as well as the data centres of the IeDEA-SA collaboration for preparing the data. We thank Christopher Ritter for editorial services.

IeDEA-Southern Africa Steering Committee

Matthias Egger (co-PI), University of Bern, Switzerland; **Mary-Ann Davies** (co-PI), University of Cape Town, South Africa**; Frank Tanser**, Africa Centre for Health and Population Studies, University of Kwazulu-Natal, South Africa; **Michael Vinikoor**, Centre for Infectious Disease Research in Zambia; **Eusebio Macete**, Centro de Investigação em Saúde de Manhiça, Mozambique; **Robin Wood**, Desmond Tutu HIV Centre (Gugulethu and Masiphumelele clinics), South Africa**; Kathryn Stinson**, Khayelitsha ART Programme and Médecins Sans Frontières, South Africa; **Geoffrey Fatti**, Kheth'Impilo Programme, South Africa; **Sam Phiri**, Lighthouse Trust Clinic, Malawi; **Cleophas Chimbetete**, Newlands Clinic, Zimbabwe; **Kennedy Malisita**, Queen Elizabeth Hospital, Malawi; **Brian Eley**, Red Cross War Memorial Children's Hospital and Department of Paediatrics and Child Health, University of Cape Town, South Africa; **Jochen Ehmer**, Solidarmed, Switzerland; **Christiane Fritz**, SolidarMed SMART Programme, Lesotho; **Michael Hobbins**, SolidarMed SMART Programme, Mozambique; **Kamelia Kamenova**, SolidarMed SMART Programme, Zimbabwe; **Matthew Fox**, Themba Lethu Clinic, South Africa; **Hans Prozesky**, Tygerberg Academic Hospital, South Africa; **Karl Technau**, Empilweni Clinic, Rahima Moosa Mother and Child Hospital, South Africa; **Shobna Sawry**, Harriet Shezi Children's Clinic, Chris Hani Baragwanath Academic Hospital, South Africa.

For peer review only

References

1 Naranbat N, Nymadawa P, Schopfer K, *et al.* Seasonality of tuberculosis in an Eastern-Asian country with an extreme continental climate. *Eur Respir J* 2009;**34**:921–5. doi:10.1183/09031936.00035309

2 Mabaera B, Naranbat N, Katamba A, *et al.* Seasonal variation among tuberculosis suspects in four countries. *Int Health* 2009;**1**:53–60. doi:10.1016/j.inhe.2009.06.006

3 Willis MD, Winston CA, Heilig CM, *et al.* Seasonality of tuberculosis in the United States, 1993-2008. *Clin Infect Dis* 2012;**54**:1553–60. doi:10.1093/cid/cis235

4 Thorpe LE, Frieden TR, Laserson KF, *et al.* Seasonality of tuberculosis in India: is it real and what does it tell us? *Lancet* 2004;**364**:1613–4. doi:10.1016/S0140-6736(04)17316-9

5 Wubuli A, Li Y, Xue F, *et al.* Seasonality of active tuberculosis notification from 2005 to 2014 in Xinjiang, China. *PLOS ONE* 2017;**12**:e0180226. doi:10.1371/journal.pone.0180226

6 Margalit I, Block C, Mor Z. Seasonality of tuberculosis in Israel, 2001-2011. *Int J Tuberc Lung Dis* 2016;**20**:1588–93. doi:10.5588/ijtld.16.0306

7 Fares A. Seasonality of tuberculosis. *J Glob Infect Dis* 2011;**3**:46–55. doi:10.4103/0974-777X.77296

8 Martineau AR, Nhamoyebonde S, Oni T, *et al.* Reciprocal seasonal variation in vitamin D status and tuberculosis notifications in Cape Town, South Africa. *Proc Natl Acad Sci U S A* 2011;**108**:19013–7. doi:10.1073/pnas.1111825108

9 Wingfield T, Schumacher SG, Sandhu G, *et al.* The seasonality of tuberculosis, sunlight, vitamin d, and household crowding. *J Infect Dis* 2014;**210**:774–83. doi:10.1093/infdis/jiu121

10 Balcells ME, García P, Tiznado C, *et al.* Association of vitamin D deficiency, season of the year, and latent tuberculosis infection among household contacts. *PLOS ONE* 2017;**12**:e0175400. doi:10.1371/journal.pone.0175400

11 Corbett EL, Watt CJ, Walker N, *et al.* The growing burden of tuberculosis: global trends and interactions with the HIV epidemic. *Arch Intern Med* 2003;**163**:1009–21. doi:10.1001/archinte.163.9.1009

12 Pawlowski A, Jansson M, Skold M, *et al.* Tuberculosis and HIV co-infection. *PLoS Pathog* 2012;**8**:e1002464. doi:10.1371/journal.ppat.1002464

13 World Health Organization. Global tuberculosis report 2015. WHO 2015. http://www.who.int/tb/publications/global_report/en/

For peer review only - http://bmjopen.bmj.com/site/about/guidelines.xhtml

14 Egger M, Ekouevi DK, Williams C, *et al.* Cohort Profile: the international epidemiological databases to evaluate AIDS (IeDEA) in sub-Saharan Africa. *Int J Epidemiol* 2012;**41**:1256–64. doi:10.1093/ije/dyr080

15 Fenner L, Ballif M, Graber C, *et al.* Tuberculosis in antiretroviral treatment programs in lower income countries: availability and use of diagnostics and screening. *PloS One* 2013;**8**:e77697. doi:10.1371/journal.pone.0077697

16 Cape Town. Wikipedia. 2016.https://en.wikipedia.org/w/index.php?title=Cape_Town&oldid=748785698 (accessed 10 Nov 2016).

17 Johannesburg. Wikipedia. 2016.https://en.wikipedia.org/w/index.php?title=Johannesburg&oldid=748810210 (accessed 10 Nov 2016).

18 Harare. Wikipedia. 2016.https://en.wikipedia.org/w/index.php?title=Harare&oldid=746466670 (accessed 10 Nov 2016).

19 Lusaka. Wikipedia. 2016.https://en.wikipedia.org/w/index.php?title=Lusaka&oldid=748771985 (accessed 10 Nov 2016).

20 Altman DG. Practical Statistics for Medical Research - Relationship between two continuous variables. Chapman and Hall/CRC. 1990.

21 Top R, Boshuizen H, Dekkers A, *et al.* Similar seasonal peak in clustered and unique extra-pulmonary tuberculosis notifications: winter crowding hypothesis ruled out? *Int J Tuberc Lung Dis* 2013;**17**:1466–71. doi:10.5588/ijtld.13.0226

22 Schwitters A, Lederer P, Zilversmit L, *et al.* Barriers to Health Care in Rural Mozambique: A Rapid Ethnographic Assessment of Planned Mobile Health Clinics for ART. *Glob Health Sci Pract* 2015;**3**:109–16. doi:10.9745/GHSP-D-14-00145

23 Johnson DC, Feldacker C, Tweya H, *et al.* Factors associated with timely initiation of antiretroviral therapy in two HIV clinics in Lilongwe, Malawi. *Int J STD AIDS* 2013;**24**:42–9. doi:10.1177/0956462412472312

24 Sandelowski M, Barroso J, Voils CI. Gender, Race/Ethnicity, and Social Class in Research Reports on Stigma in HIV-Positive Women. *Health Care Women Int* 2009;**30**:273–88. doi:10.1080/07399330802694880

25 Tun W, Okal J, Schenk K, *et al.* Limited accessibility to HIV services for persons with disabilities living with HIV in Ghana, Uganda and Zambia. *J Int AIDS Soc* 2016;**19**. doi:10.7448/IAS.19.5.20829

For peer review only

For peer review only - http://bmjopen.bmj.com/site/about/guidelines.xhtml

26 Getahun H, Gunneberg C, Granich R, *et al.* HIV infection-associated tuberculosis: the epidemiology and the response. *Clin Infect Dis* 2010;**50 Suppl 3**:S201-7. doi:10.1086/651492

27 Braitstein P, Boulle A, Nash D, *et al.* Gender and the use of antiretroviral treatment in resource-constrained settings: findings from a multicenter collaboration. *J Womens Health 2002* 2008;**17**:47–55. doi:10.1089/jwh.2007.0353

28 Holmes CB, Hausler H, Nunn P. A review of sex differences in the epidemiology of tuberculosis. *Int J Tuberc Lung Dis* 1998;**2**:96–104.

29 Fenner L, Forster M, Boulle A, *et al.* Tuberculosis in HIV programmes in lower-income countries: practices and risk factors. *Int J Tuberc Lung Dis* 2011;**15**:620–7. doi:10.5588/ijtld.10.0249

30 Daley P, Jagannathan V, John KR, *et al.* Adjunctive vitamin D for treatment of active tuberculosis in India: a randomised, double-blind, placebo-controlled trial. *Lancet Infect Dis* 2015;**15**:528–34. doi:10.1016/S1473-3099(15)70053-8

31 Coussens AK, Wilkinson RJ, Hanifa Y, *et al.* Vitamin D accelerates resolution of inflammatory responses during tuberculosis treatment. *Proc Natl Acad Sci U S A* 2012;**109**:15449–54. doi:10.1073/pnas.1200072109

32 Ralph AP, Waramori G, Pontororing GJ, *et al.* L-arginine and Vitamin D Adjunctive Therapies in Pulmonary Tuberculosis: A Randomised, Double-Blind, Placebo-Controlled Trial. *PLOS ONE* 2013;**8**:e70032. doi:10.1371/journal.pone.0070032

33 Lawn SD, Harries AD, Meintjes G, *et al.* Reducing deaths from tuberculosis in antiretroviral treatment programmes in sub-Saharan Africa. *AIDS Lond Engl* 2012;**26**:2121–33. doi:10.1097/QAD.0b013e3283565dd1

34 Lawn SD, Bekker L-G, Wood R. How effectively does HAART restore immune responses to Mycobacterium tuberculosis? Implications for tuberculosis control. *AIDS Lond Engl* 2005;**19**:1113–24.

35 Lawn SD, Badri M, Wood R. Tuberculosis among HIV-infected patients receiving HAART: long term incidence and risk factors in a South African cohort. *AIDS Lond Engl* 2005;**19**:2109–16.

36 Baker M, Das D, Venugopal K, *et al.* Tuberculosis associated with household crowding in a developed country. *J Epidemiol Community Health* 2008;**62**:715–21. doi:10.1136/jech.2007.063610

37 Lönnroth K, Jaramillo E, Williams BG, *et al.* Drivers of tuberculosis epidemics: the role of risk factors and social determinants. *Soc Sci Med 1982* 2009;**68**:2240–6. doi:10.1016/j.socscimed.2009.03.041

38 Lessells RJ, Swaminathan S, Godfrey-Faussett P. HIV treatment cascade in tuberculosis patients. *Curr Opin HIV AIDS* 2015;**10**:439–46. doi:10.1097/COH.0000000000000197

For peer review only

39 Tweya H, Feldacker C, Gadabu OJ, *et al.* Developing a point-of-care electronic medical record system for TB/HIV co-infected patients: experiences from Lighthouse Trust, Lilongwe, Malawi. *BMC Res Notes* 2016;**9**:146. doi:10.1186/s13104-016-1943-4

40 Charles MK, Lindegren ML, Wester CW, *et al.* Implementation of Tuberculosis Intensive Case Finding, Isoniazid Preventive Therapy, and Infection Control ("Three I's") and HIV-Tuberculosis Service Integration in Lower Income Countries. *PloS One* 2016;**11**:e0153243. doi:10.1371/journal.pone.0153243

41 World Health Organization. WHO policy on collaborative TB/HIV activities: guidelines for national programmes and other stakeholders. Published Online First: 2012.http://www.who.int/tb/publications/2012/tb_hiv_policy_9789241503006/en/index.html

For peer review only

For peer review only - http://bmjopen.bmj.com/site/about/guidelines.xhtml

Figure and tables

Table 1: Characteristics of participating ART programs.

Characteristics	South Africa – Khayelitsha	South Africa – Themba Lethu	South Africa – Tygerberg	Zambia – CIDRZ	Zimbabwe – Newlands
**Geographical latitude (°)**	-34.05	-33.91	-26.12	-15.41	-17.81
**Setting**	Urban	Urban	Urban	Combination of urban and peri-urban health centres	Urban
**Level of care**	Primary	Secondary	Tertiary	Primary	Primary
**TB case definition**	Majority microbiologically confirmed, others based on clinical presentation	Microbiologically confirmed	Majority microbiologically confirmed, others based on clinical presentation	Majority microbiologically confirmed, others based on clinical presentation	Majority microbiologically confirmed, others based on clinical presentation
**Cost of TB diagnosis to patients**	Available at no cost	Available at no cost	Partial payment	Available at no cost or partial payment (depending on facility)	Available at no cost
**Co-trimoxazole for prevention**	All patients	According to CD4 cell count	According to CD4 cell count	According to CD4 cell count or to all patients (depending on facility)	According to CD4 cell count
**Integration of HIV/TB clinical services**	Same facility / same day appointments	Same facility / same day appointments	Cross referral	Cross referral	Same facility / same day appointments
**TB screening at ART enrolment**	All patients, symptomatic screening and diagnostic tests	All patients, symptomatic screening only	All patients, symptomatic screening only	All patients, symptomatic screening and diagnostic tests at selected sites	All patients, symptomatic screening only

Data sources: Ref. 15.

Abbreviations: ART, antiretroviral therapy; TB, tuberculosis.

For peer review only - http://bmjopen.bmj.com/site/about/guidelines.xhtml

449 **Table 2:** Characteristics of patients diagnosed with pulmonary tuberculosis between January
450 2004 and December 2014 compared to others.

Characteristics	Patients diagnosed with PTB	Other patients	All
**Age at ART start (median, IQR), years**	35 (30-41)	34 (29-41)	34 (29-41)
Missing, n (%)	*0*	*0*	*0*
**Gender, n (%)**			
Female	25,227 (51.1)	181,847 (64.4)	207,074 (62.4)
Male	24,140 (48.9)	100,420 (35.6)	124,560 (37.6)
Missing, n (%)	*0*	*0*	*0*
**CD4 cell count at ART start (median, IQR), cells/µl**	116 (52-200)	166 (87-254)	158 (80-246)
Missing, n (%)	*24,237 (48.8)*	*150,058 (53.2)*	*174,295 (52.6)*
**WHO clinical stage, n (%)**			
Stage 1 and 2	4,916 (9.9)	157,322 (55.7)	162,238 (48.9)
Stage 3 and 4	41,744 (84.6)	106,836 (37.9)	148,580 (44.8)
Unknown	2,707 (5.5)	18,053 (6.4)	20,816 (6.3)
Missing, n (%)	*0*	*56 (0.02)*	*56 (0.02)*
**Treatment program, n (%)**			
South Africa – Khayelitsha	7,357 (14.9)	25,021 (8.9)	32,378 (9.8)
South Africa – Themba Lethu	5,438 (11.0)	16,967 (6.0)	22,405 (6.8)
South Africa – Tygerberg	861 (1.7)	3,657 (1.3)	4,518 (1.4)
Zambia – CIDRZ	35,132 (71.2)	233,680 (82.8)	268,812 (81.1)
Zimbabwe – Newlands	579 (1.2)	2,942 (1.0)	3,521 (1.1)
Missing, n (%)	*0*	*0*	*0*
**Total, n (%)**	**49,367 (14.9)**	**282,267 (85.1)**	**331,634 (100)**

451

452 Abbreviations: ART, antiretroviral therapy; IQR, interquartile range; PTB, pulmonary
453 tuberculosis; WHO, World Health Organization.

454

For peer review only

For peer review only - http://bmjopen.bmj.com/site/about/guidelines.xhtml

Table 3: Correlation coefficients between deviations of monthly counts of antiretroviral therapy enrolments from the yearly average and deviations of monthly counts of pulmonary tuberculosis, extrapulmonary tuberculosis, and Kaposi sarcoma diagnosis from the yearly averages. Median monthly event counts (2000-2014) and Pearson's pairwise correlation coefficients are shown.

ART program	ART enrolments	PTB diagnoses			EPTB diagnoses			KS diagnoses		
	Median (IQR)	Median (IQR)	Correlation (95% CI)	p-value	Median (IQR)	Correlation (95% CI)	p-value	Median (IQR)	Correlation (95% CI)	p-value
South Africa – Khayelitsha	252 (236-265)	58 (46-64)	0.27 (0.11-0.42)	0.002	26 (24-30)	0.20 (0.03-0.36)	0.019	2 (1-2)	0.08 (-0.11-0.25)	0.42
South Africa – Themba Lethu	171 (161-180)	41 (37-44)	0.22 (0.05-0.38)	0.010	16 (14-17)	0.15 (-0.03-0.31)	0.097	1 (1-1)	0.02 (-0.16-0.19)	0.86
South Africa – Tygerberg	34 (34-37)	7 (6-8)	0.19 (0.02-0.35)	0.028	3 (3-3)	0.09 (-0.09-0.25)	0.33	0.3 (0.2-0.5)	0.11 (-0.06-0.28)	0.22
Zambia – CIDRZ	2163 (2095-2233)	275 (259-301)	0.82 (0.75-0.87)	<0.001	9 (9-10)	0.54 (0.40-0.65)	<0.001	29 (28-31)	0.68 (0.57-0.77)	<0.001
Zimbabwe – Newlands	31 (29-34)	5 (4-6)	0.20 (0.02-0.37)	0.029	2 (2-2)	0.23 (0.06-0.40)	0.011	0.2 (0.1-0.4)	0.22 (0.05-0.39)	0.014
**All**	**170 (34-264)**	**40 (6-63)**	**0.28 (0.21-0.35)**	**<0.001**	**9 (3-17)**	**0.25 (0.18-0.32)**	**<0.001**	**1 (0-2)**	**0.14 (0.07-0.22)**	**<0.001**

Abbreviations: ART, antiretroviral therapy; 95% CI, 95% confidence interval; EPTB, extrapulmonary tuberculosis; IQR, interquartile range; KS, Kaposi sarcoma; PTB, pulmonary tuberculosis.

For peer review only - http://bmjopen.bmj.com/site/about/guidelines.xhtml

Table 4: Regression coefficients between deviations of monthly counts of pulmonary tuberculosis, extrapulmonary tuberculosis, or Kaposi sarcoma diagnoses and deviations of monthly counts of antiretroviral therapy enrolments from the yearly averages (unadjusted); adjusted for the deviations of monthly air temperatures to yearly average air temperatures at the ART program ("adjusted for temperature"). The overall regression model was also adjusted for the latitude of the ART program's location ("adjusted for temperature and latitude"). Monthly air temperatures are based on 1961-1990 averages.

ART program	PTB diagnoses		EPTB diagnoses		KS diagnoses	
	Coefficient (95% CI)	p-value	Coefficient (95% CI)	p-value	Coefficient (95% CI)	p-value
**South Africa – Khayelitsha**						
Unadjusted	0.33 (0.13-0.53)	0.002	0.28 (0.05-0.51)	0.019	0.30 (-0.43-1.03)	0.416
Adjusted for temperature	0.30 (0.09-0.51)	0.005	0.27 (0.03-0.51)	0.027	0.44 (-0.31-1.12)	0.248
**South Africa – Themba Lethu**						
Unadjusted	0.16 (0.04-0.28)	0.010	0.21 (-0.04-0.45)	0.097	0.07 (-0.74-0.88)	0.860
Adjusted for temperature	0.17 (0.05-0.29)	0.005	0.21 (-0.03-0.46)	0.089	0.10 (-0.71-0.92)	0.805
**South Africa – Tygerberg**						
Unadjusted	0.41 (0.04-0.78)	0.028	0.20 (-0.20-0.60)	0.328	0.82 (-0.49-2.14)	0.218
Adjusted for temperature	0.41 (0.05-0.78)	0.028	0.21 (-0.20-0.62)	0.307	0.92 (-0.39-2.23)	0.169
**Zambia – CIDRZ**						
Unadjusted	0.84 (0.74-0.95)	<0.001	1.39 (1.00-1.78)	<0.001	0.91 (0.73-1.08)	<0.001
Adjusted for temperature	0.83 (0.72-0.94)	<0.001	1.40 (1.00-1.80)	<0.001	0.90 (0.72-1.08)	<0.001
**Zimbabwe – Newlands**						
Unadjusted	0.29 (0.03-0.54)	0.029	0.47 (0.11-0.83)	0.011	1.32 (0.27-2.38)	0.014
Adjusted for temperature	0.29 (0.037-0.55)	0.025	0.47 (0.12-0.84)	0.010	1.31 (0.26-2.36)	0.015
**All**						
Unadjusted	0.37 (0.27-0.47)	<0.001	0.50 (0.35-0.65)	<0.001	0.75 (0.35-1.16)	<0.001
Adjusted for temperature	0.38 (0.28-0.48)	<0.001	0.51 (0.36-0.66)	<0.001	0.77 (0.36-1.18)	<0.001
Adjusted for temperature and latitude	0.38 (0.28-0.48)	<0.001	0.51 (0.36-0.66)	<0.001	0.77 (0.36-1.18)	<0.001

For peer review only

Abbreviations: EPTB, extrapulmonary tuberculosis; 95% CI, 95% confidence interval; KS, Kaposi sarcoma; PTB, pulmonary tuberculosis.

Figure Legends

Figure 1: Selection of patients for the analysis.

Figure 2: Aggregated averages in monthly counts of patients newly started on antiretroviral therapy and monthly counts of pulmonary tuberculosis diagnosis (aggregate averages 2004-2014, logarithmic scale). Dashed horizontal lines indicate overall monthly means.

Figure 3: Relationship between pulmonary tuberculosis (PTB) diagnosis and geographical latitude: regression coefficients between deviations of monthly counts of PTB diagnoses and deviations of monthly counts of antiretroviral therapy enrolments from the yearly averages (adjusted for air temperature) according to the geographical latitude of the ART program's location.

For peer review only

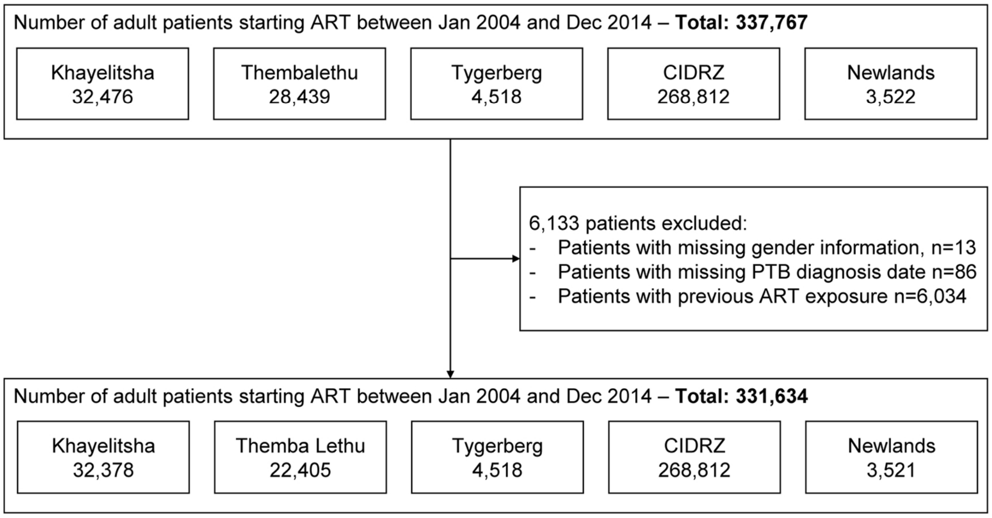

Figure 1: Selection of patients for the analysis.
Abbreviations: ART, antiretroviral therapy; PTB, pulmonary tuberculosis

146x76mm (300 x 300 DPI)

review only

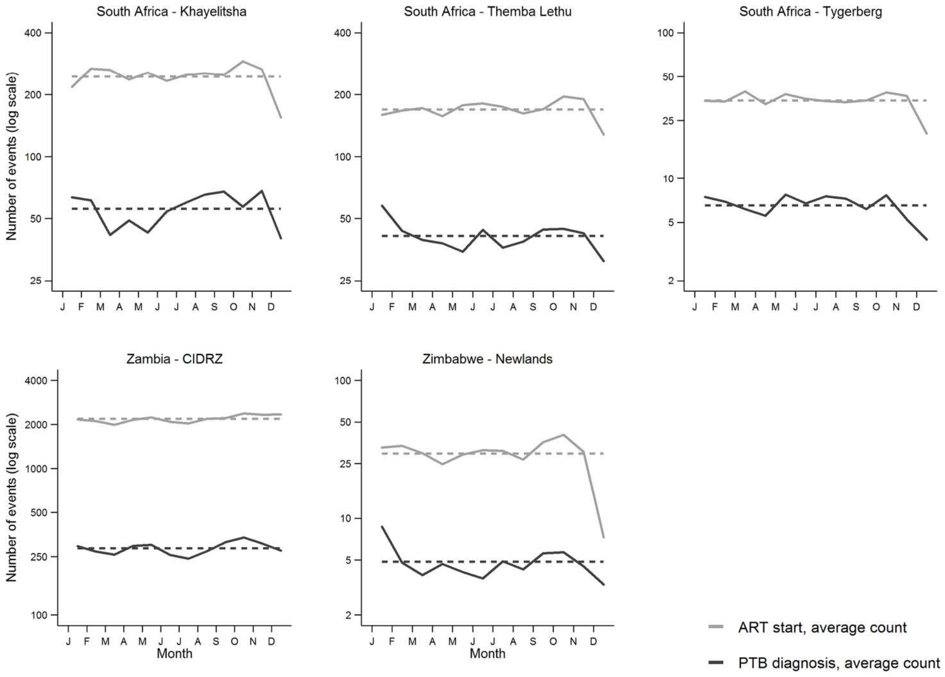

Figure 2: Aggregated averages in monthly counts of patients newly started on antiretroviral therapy and monthly counts of pulmonary tuberculosis diagnosis (aggregate averages 2004-2014, logarithmic scale). Dashed horizontal lines indicate overall monthly means.
Abbreviations: ART, antiretroviral therapy; PTB, pulmonary tuberculosis

182x132mm (300 x 300 DPI)

w only

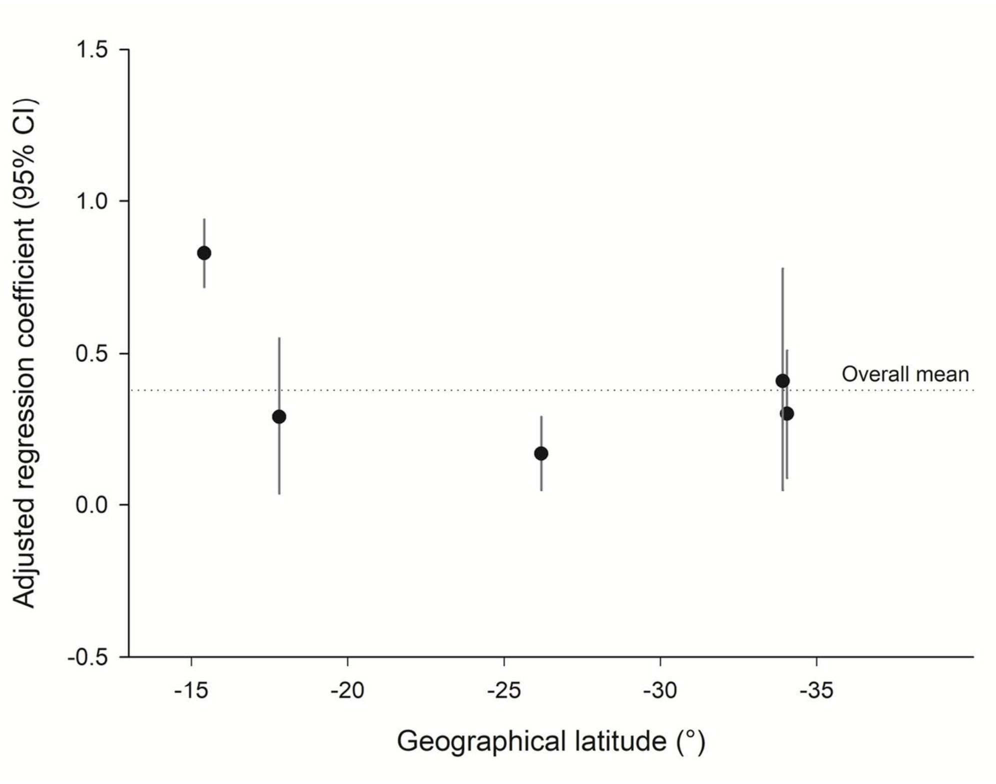

Figure 3: Relationship between pulmonary tuberculosis (PTB) diagnosis and geographical latitude: regression coefficients between deviations of monthly counts of PTB diagnoses and deviations of monthly counts of antiretroviral therapy enrolments from the yearly averages (adjusted for air temperature) according to the geographical latitude of the ART program's location.

115x89mm (300 x 300 DPI)

Supplementary File

Supplementary Figure 1: Quarterly counts of patients newly started on antiretroviral therapy, and quarterly counts of pulmonary tuberculosis diagnoses between 2004 and 2014.

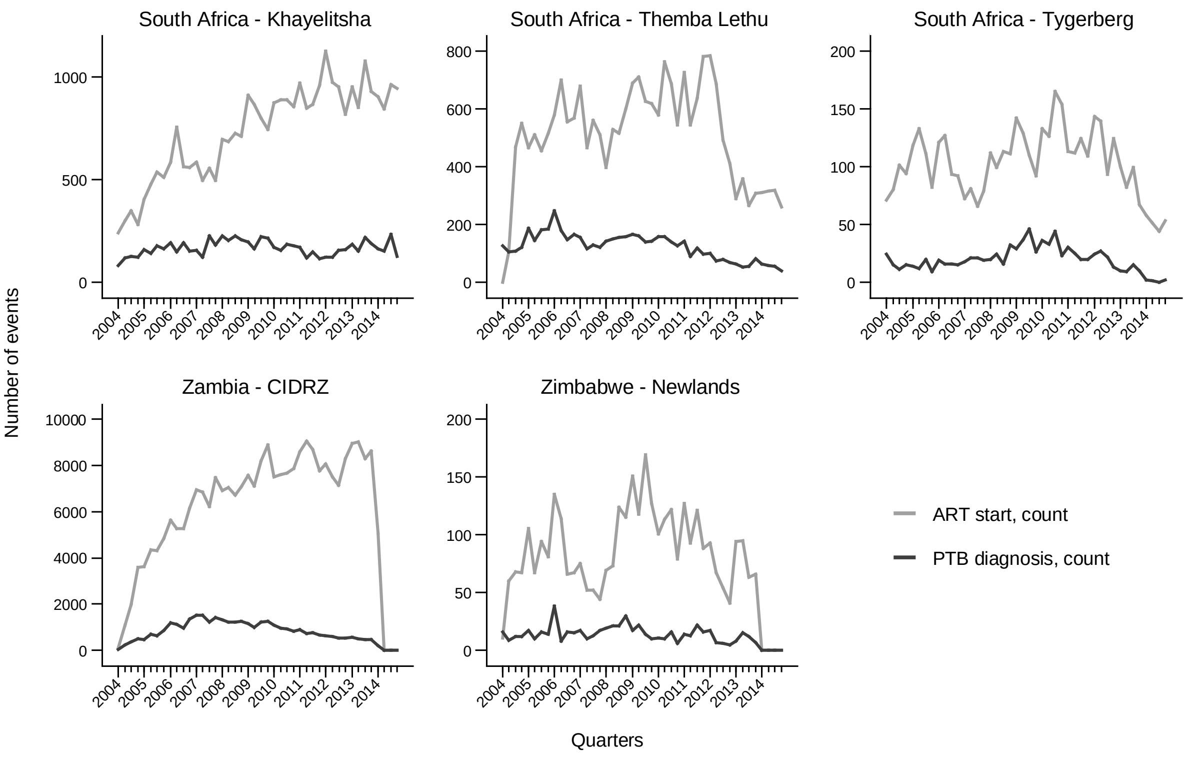

Abbreviations: ART, antiretroviral therapy; PTB, pulmonary tuberculosis

Supplementary Figure 2: Aggregated averages in monthly counts of patients newly started on antiretroviral therapy and monthly counts of pulmonary tuberculosis diagnosis (study period 2004-2014), by gender (**panel A**) and by age groups (**panel B**). Horizontal lines indicate the overall monthly mean of ART enrolment events or PTB diagnosis events.

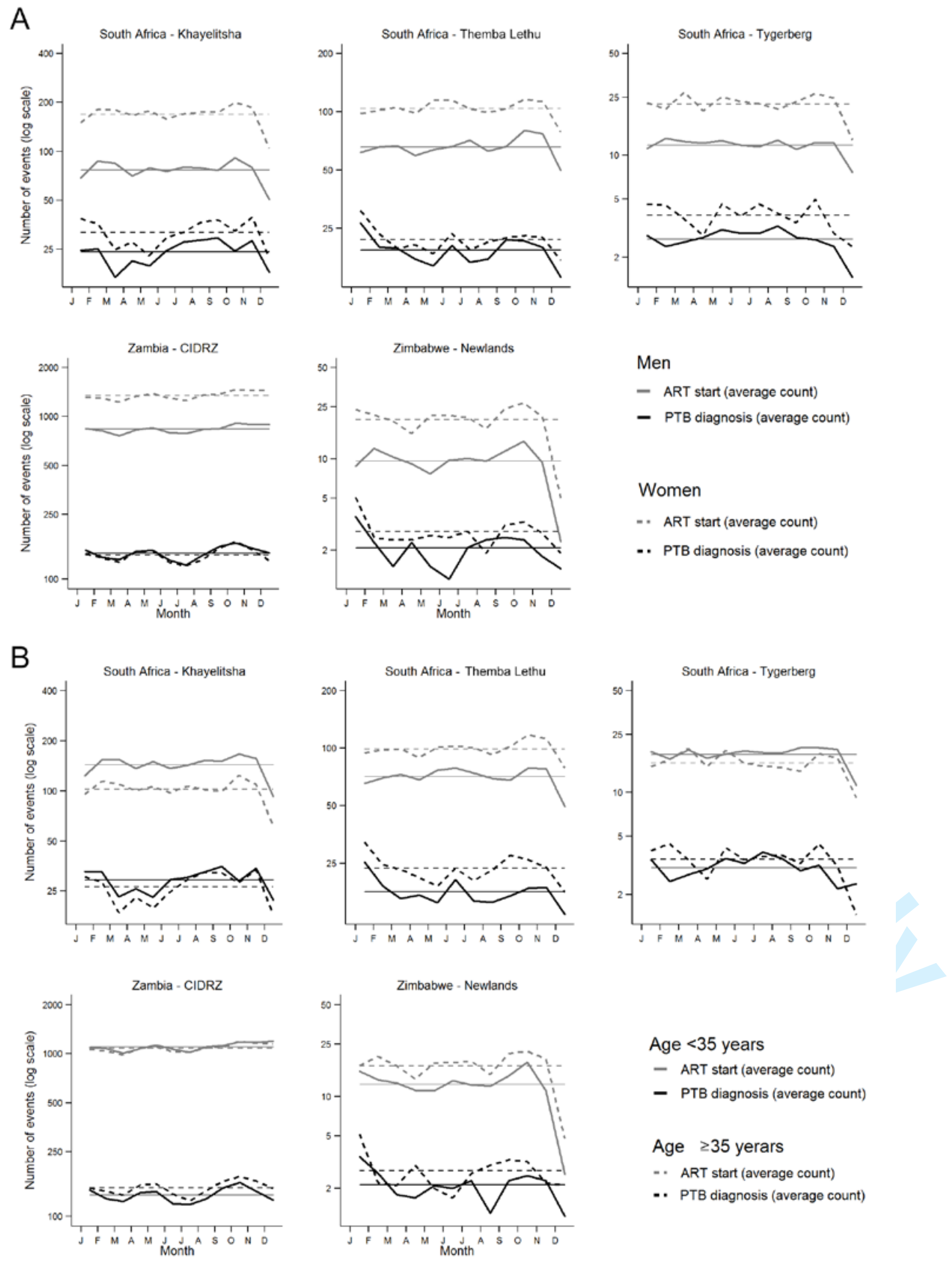

Abbreviations: ART, antiretroviral therapy; PTB, pulmonary tuberculosis.

Supplementary Figure 3: Mean deviations of monthly counts in antiretroviral therapy initiations and pulmonary tuberculosis diagnosis from the corresponding yearly averages.

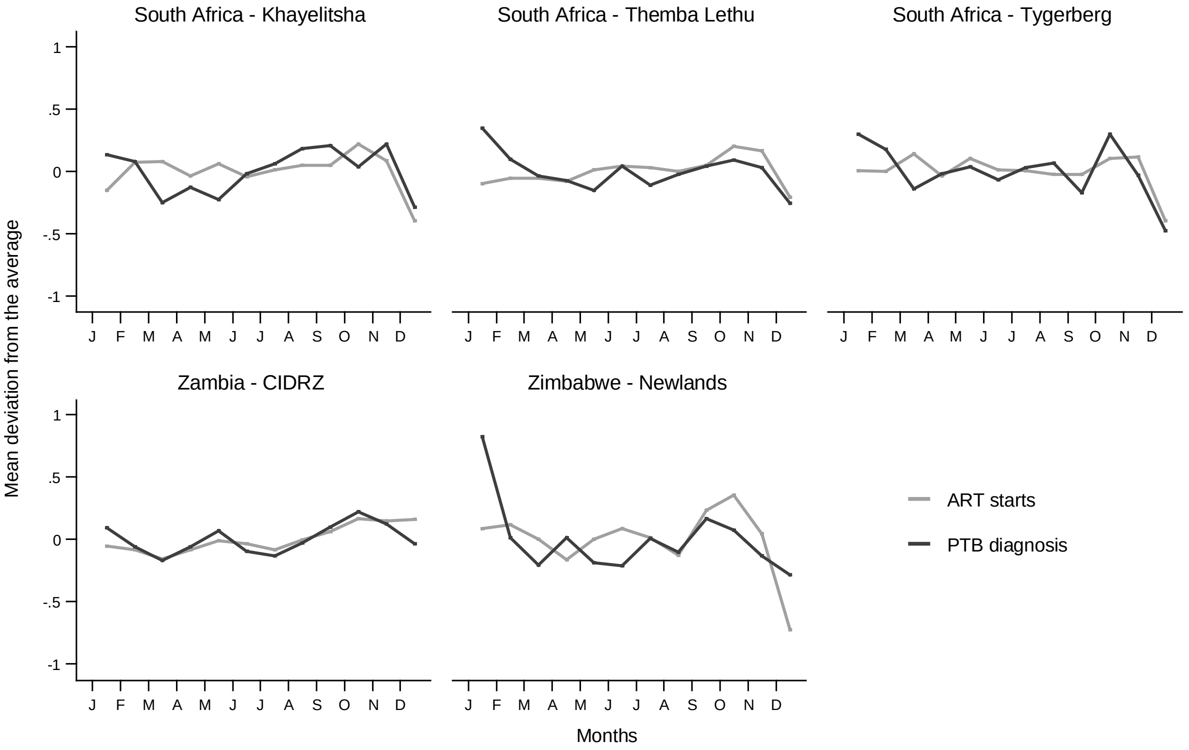

Abbreviations: ART, antiretroviral therapy; PTB, pulmonary tuberculosis

For peer review only

STROBE Statement—Checklist of items that should be included in reports of ***cohort studies***

Manuscript: Seasonal variations in tuberculosis diagnosis among HIV-coinfected individuals in Southern Africa: an observational cohort study at antiretroviral treatment programs

	Item No	Recommendation	Manuscript page number
**Title and abstract**	1	(*a*) Indicate the study's design with a commonly used term in the title or the abstract	**1, 3**
		(*b*) Provide in the abstract an informative and balanced summary of what was done and what was found	**3**
**Introduction**			
Background/rationale	2	Explain the scientific background and rationale for the investigation being reported	**5**
Objectives	3	State specific objectives, including any prespecified hypotheses	**5**
**Methods**			
Study design	4	Present key elements of study design early in the paper	**6-7**
Setting	5	Describe the setting, locations, and relevant dates, including periods of recruitment, exposure, follow-up, and data collection	**6**
Participants	6	(*a*) Give the eligibility criteria, and the sources and methods of selection of participants. Describe methods of follow-up	**6**
		(*b*) For matched studies, give matching criteria and number of exposed and unexposed	**NA**
Variables	7	Clearly define all outcomes, exposures, predictors, potential confounders, and effect modifiers. Give diagnostic criteria, if applicable	**6-7**
Data sources/ measurement	8*	For each variable of interest, give sources of data and details of methods of assessment (measurement). Describe comparability of assessment methods if there is more than one group	**6-7**
Bias	9	Describe any efforts to address potential sources of bias	**6-7**
Study size	10	Explain how the study size was arrived at	**NA**
Quantitative variables	11	Explain how quantitative variables were handled in the analyses. If applicable, describe which groupings were chosen and why	**6-7**
Statistical methods	12	(*a*) Describe all statistical methods, including those used to control for confounding	**6-7**
		(*b*) Describe any methods used to examine subgroups and interactions	**6-7**
		(*c*) Explain how missing data were addressed	**6-7**
		(*d*) If applicable, explain how loss to follow-up was addressed	**NA**
		(*e*) Describe any sensitivity analyses	**NA**
**Results**			
Participants	13*	(a) Report numbers of individuals at each stage of study—eg numbers potentially eligible, examined for eligibility, confirmed eligible, included in the study, completing follow-up, and analysed	**8 + Fig. 1**
		(b) Give reasons for non-participation at each stage	**Fig. 1**
		(c) Consider use of a flow diagram	**Fig. 1**
Descriptive data	14*	(a) Give characteristics of study participants (eg demographic, clinical, social) and information on exposures and potential	**8 + table 2**

For peer review only - http://bmjopen.bmj.com/site/about/guidelines.xhtml
For peer review only

		confounders	
		(b) Indicate number of participants with missing data for each variable of interest	**Fig. 1+ table 2**
		(c) Summarise follow-up time (eg, average and total amount)	**8**
Outcome data	15*	Report numbers of outcome events or summary measures over time	**8-9**
Main results	16	(*a*) Give unadjusted estimates and, if applicable, confounder-adjusted estimates and their precision (eg, 95% confidence interval). Make clear which confounders were adjusted for and why they were included	**8-9**
		(*b*) Report category boundaries when continuous variables were categorized	**NA**
		(*c*) If relevant, consider translating estimates of relative risk into absolute risk for a meaningful time period	**NA**
Other analyses	17	Report other analyses done—eg analyses of subgroups and interactions, and sensitivity analyses	**8-9**
**Discussion**			
Key results	18	Summarise key results with reference to study objectives	**10**
Limitations	19	Discuss limitations of the study, taking into account sources of potential bias or imprecision. Discuss both direction and magnitude of any potential bias	**12**
Interpretation	20	Give a cautious overall interpretation of results considering objectives, limitations, multiplicity of analyses, results from similar studies, and other relevant evidence	**10-12**
Generalisability	21	Discuss the generalisability (external validity) of the study results	**12**
**Other information**			
Funding	22	Give the source of funding and the role of the funders for the present study and, if applicable, for the original study on which the present article is based	**13**

*Give information separately for exposed and unexposed groups.

Note: An Explanation and Elaboration article discusses each checklist item and gives methodological background and published examples of transparent reporting. The STROBE checklist is best used in conjunction with this article (freely available on the Web sites of PLoS Medicine at http://www.plosmedicine.org/, Annals of Internal Medicine at http://www.annals.org/, and Epidemiology at http://www.epidem.com/). Information on the STROBE Initiative is available at http://www.strobe-statement.org.

For peer review only

For peer review only - http://bmjopen.bmj.com/site/about/guidelines.xhtml

BMJ Open

Seasonal variations in tuberculosis diagnosis among HIV-positive individuals in Southern Africa: analysis of cohort studies at antiretroviral treatment programs

Journal:	*BMJ Open*
Manuscript ID	bmjopen-2017-017405.R2
Article Type:	Research
Date Submitted by the Author:	31-Oct-2017
Complete List of Authors:	Ballif, Marie; University of Bern, Insitute of Social and Preventive Medicine<br>Zürcher, Kathrin; University of Bern, Insitute of Social and Preventive Medicine<br>Reid, Stewart; University of Alabama at Birmingham; Centre for Infectious Disease Research in Zambia<br>Boulle, Andrew; University of Cape Town; Médecins Sans Frontières<br>Fox, Matt; Boston University, Epidemiology and Global Health; University of the Witwatersrand<br>Prozesky, Hans; Division of Infectious Diseases, Department of Medicine, University of Stellenbosch & Tygerberg Academic Hospital<br>Chimbetete, Cleophas; Newlands Clinic<br>Zwahlen , Marcel; University of Bern, Insitute of Social and Preventive Medicine<br>Egger, Matthias; University of Bern, Institute of Social and Preventive Medicine; School of Public Health and Family Medicine, University of Cape Town<br>Fenner, Lukas; University of Bern, Institute of Social and Preventive Medicine
**Primary Subject Heading**:	HIV/AIDS
Secondary Subject Heading:	Infectious diseases, Epidemiology
Keywords:	Seasonality, pulmonary tuberculosis, HIV & AIDS < INFECTIOUS DISEASES, South Africa, Zambia, Zimbabwe

SCHOLARONE™
Manuscripts

TITLE PAGE

Seasonal variations in tuberculosis diagnosis among HIV-positive individuals in Southern Africa: analysis of cohort studies at antiretroviral treatment programs

Marie Ballif [1 §], Kathrin Zürcher [1], Stewart E. Reid [2 3], Andrew Boulle [4 5], Matthew P. Fox [6 7], Hans W. Prozesky [8], Cleophas Chimbetete [9], Marcel Zwahlen [1], Matthias Egger [1 4], Lukas Fenner [1]; for the International Epidemiology Database to Evaluate AIDS in Southern Africa (IeDEA-SA)

1 Institute of Social and Preventive Medicine, University of Bern, Bern, Switzerland

2 University of Alabama at Birmingham, Birmingham, USA

3 Centre for Infectious Disease Research in Zambia (CIDRZ), Lusaka, Zambia

4 Centre for Infectious Disease Epidemiology and Research (CIDER), School of Public Health and Family Medicine, University of Cape Town, Cape Town, South Africa

5 Médecins Sans Frontières, Khayelitsha, Cape Town, South Africa

6 Health Economics and Epidemiology Research Office, Department of Internal Medicine, School of Clinical Medicine, Faculty of Health Sciences, University of the Witwatersrand, Johannesburg, South Africa

7 Departments of Epidemiology and Global Health, Boston University, Boston, USA

8 Division of Infectious Diseases, Department of Medicine, University of Stellenbosch and Tygerberg Hospital, Cape Town, South Africa

9 Newlands Clinic, Harare, Zimbabwe

For peer review only

For peer review only - http://bmjopen.bmj.com/site/about/guidelines.xhtml

[§] **Corresponding author:**
Dr Marie Ballif
Institute of Social and Preventive Medicine, University of Bern
Finkenhubelweg 11, 3012 Bern, Switzerland
Email: marie.ballif@ispm.unibe.ch
Phone: +41 31 631 35 18 Fax: +41 31 631 35 20

Keywords:

Seasonality, pulmonary tuberculosis, antiretroviral therapy, HIV, South Africa, Zambia, Zimbabwe

Word count:

Main text 2350 (max. 4000), abstract 300 (max. 300), references: 41

Inserts:

4 tables and 3 figures, Supplementary File (3 figures)

For peer review only

Abstract

Objectives Seasonal variations in tuberculosis diagnoses have been attributed to seasonal climatic changes and indoor crowding during colder winter months. We investigated trends in pulmonary tuberculosis (PTB) diagnosis at antiretroviral therapy (ART) programs in Southern Africa.

Setting Five ART programs participating in the International Epidemiology Database to Evaluate AIDS in South Africa, Zambia, and Zimbabwe.

Participants We analyzed data of 331,634 HIV-positive adults (>15 years), who initiated ART between January 2004 and December 2014.

Primary outcome measure We calculated aggregated averages in monthly counts of PTB diagnoses and ART initiations. To account for time trends, we compared deviations of monthly event counts to yearly averages, and calculated correlation coefficients. We used multivariable regressions to assess associations between deviations of monthly ART initiation and PTB diagnosis counts from yearly averages, adjusted for monthly air temperatures and geographical latitude. As controls, we used Kaposi sarcoma and extrapulmonary tuberculosis diagnoses.

Results All programs showed monthly variations in PTB diagnoses that paralleled fluctuations in ART initiations, with recurrent patterns across 2004-2014. The strongest drops in PTB diagnoses occurred in December, followed by April-May in Zimbabwe and South Africa. This corresponded to holiday seasons, when clinical activities are reduced. We observed little monthly variation in ART initiations and PTB diagnoses in Zambia. Correlation coefficients supported parallel trends in ART initiations and PTB diagnoses (correlation coefficient: 0.28, 95% confidence interval: 0.21-0.35, p-value <0.001). Monthly temperatures and latitude did not substantially change regression coefficients between ART initiations and PTB diagnoses. Trends in Kaposi sarcoma and extrapulmonary tuberculosis diagnoses similarly followed changes in ART initiations throughout the year.

Conclusions Monthly variations in PTB diagnosis at ART programs in Southern Africa likely occurred regardless of seasonal variations in temperatures or latitude, and reflected

For peer review only

fluctuations in clinical activities and changes in health-seeking behaviour throughout the year, rather than climatic factors.

Strengths and limitations of this study

- We analyzed a large dataset from HIV-positive patients routinely collected over 10 years at ART programs in low- and middle-income countries from the Southern African region;
- Included ART programs represent a wide range of countries with different climatic seasons according to their geographical latitude;
- We compared trends in PTB diagnosis to trends in Kaposi sarcoma and extrapulmonary tuberculosis diagnoses, for which no effect due to seasonal climatic changes was expected;
- Our study is limited by the lack of social and environmental information on participants (socioeconomic factors, housing conditions);
- Trends in tuberculosis diagnosis and other opportunistic infections in HIV-positive individuals may differ from trends in HIV-negative populations.

For peer review only - http://bmjopen.bmj.com/site/about/guidelines.xhtml

Introduction

Countries in different regions of the world have reported seasonal fluctuations in tuberculosis (TB) cases [1–6]. In temperate climate zones, peaks in TB diagnosis are generally observed in the spring, following the colder times of the year. The reasons for these variations are multiple, with both social and environmental factors likely playing a role [7]. Peaks in TB diagnosis following winter time have been associated with several factors: more time spent indoor in poorly ventilated rooms, which increases the risk of TB transmission; poorer access to health care when rough weather conditions make transport difficult; lower vitamin D levels due to reduced sunlight exposure, which impairs adequate immune response against infections [1,3,5,8–10].

In 2015, an estimated 1.2 million of people living with HIV developed TB, and one third of them died from TB, making TB the leading cause of death in this population [11–13]. However, little is known about the seasonal variation of TB diagnoses among HIV-positive individuals in care at antiretroviral therapy (ART) programs in sub-Saharan Africa, where the burden of HIV/TB coinfection is the highest [13]. We hypothesized that fluctuations in TB diagnoses throughout the year would likely be stronger in countries located farther away from the equator and that experience more pronounced climatic changes, as opposed to countries located closer to the equator. We investigated seasonal trends in pulmonary TB (PTB) diagnosis at five ART programs participating in the International Epidemiology Databases to Evaluate AIDS collaboration in Southern Africa (IeDEA-SA). These ART programs are located at different geographical latitudes in South Africa, Zambia, and Zimbabwe and present differently marked climatic seasons.

For peer review only - http://bmjopen.bmj.com/site/about/guidelines.xhtml

Methods

We included all HIV-positive persons age 16 and older who enrolled between January 1, 2004 and December 31, 2014 in five ART programs that participate in the IeDEA-SA collaboration (www.iedea-sa.org) and systematically collect information on opportunistic infections (OIs) [14]: Khayelitsha ART program, Themba Lethu clinic, and Tygerberg Academic Hospital in South Africa; Centre for Infectious Disease Research in Zambia (CIDRZ); and Newlands Clinic in Zimbabwe. IeDEA-SA sites provide data collected as part of routine HIV care, including TB diagnosis, at enrolment in the ART program and at each follow-up visit. Site characteristics are shown in Table 1 [15], the patient selection in Figure 1.

All study sites have approval from a local institutional review board or ethics committee to collect data and participate in IeDEA-SA projects. In addition, this project was approved by the Cantonal Ethics Committee Bern (swissethics, Bern, Switzerland) and the University of Cape Town Human Research Ethics Committee (Cape Town, South Africa).

PTB was defined according to the case definition in use at the participating IeDEA-SA sites (Table 1) [15]. We restricted the main analysis to PTB, because the diagnosis of PTB has been associated with seasonal climatic variations [1,2,7]. For comparison, we analysed monthly counts of patients diagnosed with extrapulmonary TB (EPTB) and the AIDS-defining cancer Kaposi sarcoma (KS), which are not expected to be seasonal. One record per patient was analysed. If a patient had several episodes of the same disease (PTB, EPTB, or KS), we only considered the first event of each. If a patient was diagnosed with multiple diseases of interest, priority was given to the record with PTB diagnosis, followed by EPTB and KS. We obtained mean monthly air temperatures (°C) based on 1961–1990 averages at each location [16–19].

For each site, we calculated aggregated averages as the average number of events per calendar month for 2004-2014. We accounted for calendar time trends over the study period by comparing deviations of monthly event counts to yearly averages. Site-specific aggregated averages and monthly deviations were calculated for ART enrolments and for PTB, EPTB, and KS diagnoses, overall or stratified by gender and age. We assessed the association between monthly deviations in the number of ART enrolments and the number of PTB diagnoses by

For peer review only - http://bmjopen.bmj.com/site/about/guidelines.xhtml

calculating pairwise Pearson's correlation coefficients [20]. We used Fisher's transformation to calculate 95% confidence intervals (CI) for the correlation coefficient. Finally, we used linear regression models to assess associations between the monthly deviations of PTB, EPTB or KS diagnosis counts and the monthly deviations of ART initiation counts, unadjusted and adjusted for monthly deviations of air temperatures from yearly averages and for the geographical latitude of the ART programs' locations.

All analyses were performed in Stata version 14.1 (Stata Corporation, College Station, TX, USA).

For peer review only

For peer review only - http://bmjopen.bmj.com/site/about/guidelines.xhtml

Results

We analysed data of 331,634 HIV-positive adults (Figure 1). Of these 49,367 (14.9%) were diagnosed with PTB. Median follow-up time was 2.0 years (interquartile range [IQR], 0.7-4.2), patients' median age was 34 years (IQR, 29-41), and 207,074 (62.4%) were women (Table 2). Median CD4 cell count at ART initiation of these patients was 116 cells/mm^3 (IQR: 52-200); this compares to a median cell count of 166 (87-254) in patients who were never diagnosed with PTB.

The mean number of PTB diagnoses per month was 55.7 (range 40.2-67.8) in Khayelitsha, 41.2 (31.1-57.6) in Themba Lethu, 6.5 (3.8-7.7) in Tygerberg, 285.6 (243.2-338.2) in CIDRZ, and 4.8 (3.3-8.7) in Newlands (Figure 2). At each site, monthly variations in numbers of PTB diagnoses were parallel to trends in ART enrolments. Aggregated averages over the study period showed that monthly fluctuations in PTB diagnoses were present at all sites (Figure 2). The strongest fluctuations were seen in Zimbabwe (Newlands), with PTB diagnosis peaks in January and troughs in December (+87% and -33% compared to the monthly average, respectively). Similar to Zimbabwe, all three South African sites showed marked drops in ART initiations and PTB diagnoses in December, in addition to a milder drop in April-May. In contrast, Zambia (CIDRZ) showed little variation in ART enrolments and PTB diagnoses throughout the year. We consistently observed a similar seasonal pattern in patients newly starting ART and PTB diagnoses every year (Supplementary Figure 1), in men and women, as well as in younger and older patients (Supplementary Figure 2).

The deviations of monthly event counts from the yearly averages confirmed the congruent fluctuations of ART initiation and PTB diagnosis (Supplementary Figure 3). This was further supported by the correlation coefficients between the deviations of monthly ART initiations from yearly averages and the deviations of monthly PTB diagnoses from yearly averages (Table 3): coefficient was 0.27 in Khayelitsha (95% confidence interval [CI] 0.11-0.42, p=0.002), 0.22 in Themba Lethu (95% CI 0.05-0.38, p=0.010), 0.19 in Tygerberg (95% CI 0.02-0.35, p=0.028), 0.82 in CIDRZ (95% CI 0.75-0.87, p-value<0.001), and 0.20 in Newlands (95% CI 0.02-0.37, p=0.029). Regression coefficients between deviations of monthly PTB diagnosis counts and monthly ART initiation counts from yearly averages did not substantially change

For peer review only - http://bmjopen.bmj.com/site/about/guidelines.xhtml

when adjusted for monthly temperatures and latitude (Table 4). This was further supported by Figure 3, which showed no evidence for a relationship between the adjusted regression coefficients and the latitude of the ART program's location (r=0.43, p=0.47).

To determine whether the diagnosis of other OIs followed trends similar to that of PTB and ART initiations, we studied the monthly diagnoses of EPTB and KS, which we did not expect to be influenced by climatic factors. The seasonal pattern of EPTB diagnoses paralleled that of ART initiation at most sites despite low numbers of EPTB diagnoses (1.8 to 26.5 mean monthly EPTB diagnoses). The deviations of monthly ART initiations from the yearly averages correlated with those of EPTB diagnoses in Khayelitsha, CIDRZ, and Newlands (correlation coefficient range: 0.20-0.54, Table 3). Although KS was a rare diagnosis averaging 1.1 cases per month overall, we observed that the fluctuations in KS diagnosis counts similarly followed changes in ART enrolment at all sites (Table 3). As for PTB diagnoses, the adjusted regression models did not support any influence of temperature or latitude on the observed fluctuations in EPTB or KS diagnoses (Table 4).

For peer review only

For peer review only - http://bmjopen.bmj.com/site/about/guidelines.xhtml

Discussion

We observed monthly variations in PTB diagnosis at ART programs in South Africa, Zambia and Zimbabwe. These fluctuations occurred regardless of latitudes and followed trends in ART enrolment rather than seasonal variations in temperatures, hence mirroring fluctuations in clinical activity. In South Africa and Zimbabwe, a marked drop of ART enrolments and PTB diagnoses was seen in December, followed by a rebound in January-February. A milder drop was also observed in South African sites around April-May.

In Southern Africa, seasons are marked by cooler winters and warmer summers in Cape Town (Khayelitsha, Tygerberg), and become less pronounced as latitude decreases, in Johannesburg (Themba Lethu), Zimbabwe (Newlands) and Zambia (CIDRZ). Therefore, we expected to see stronger fluctuations in PTB diagnosis, with peaks following wintertime, in the Cape Town clinics situated at the Southern tip of South Africa, than in Johannesburg (Themba Lethu clinic), Zimbabwe (Newlands clinic) and Zambia (CIDRZ), where winter months are mild. Instead, we observed variations in PTB that seemed independent from climatic changes. This might be explained by mild winters in Southern Africa, contrasting with countries with harsh winters. Mongolia typically has cold winters and strong seasonal patterns in TB notifications, likely due to increased airborne transmission from infectious PTB individuals resulting from indoor crowding and poorer ventilation during winter [1,2]. Nevertheless, seasonal variations in TB notifications in a population-based study of HIV-positive and HIV-negative individuals were documented in Cape Town, South Africa [8].

We observed that variations in PTB diagnoses followed fluctuations in ART initiations regardless of temperatures and latitudes, and hence likely reflected activities at the clinic, rather than any climatic effects. This was supported by an association between changes in ART initiation and PTB diagnosis counts (unadjusted and adjusted for monthly climatic temperatures and latitude). Furthermore, important drops in ART initiations and PTB diagnoses were observed every year in December in South Africa and Zimbabwe, when clinical activities are reduced due to the holiday season. December drops were followed by peaks in January and February, when clinical activity resumed. Similar declines in PTB diagnoses at the end of the year have been reported in Zimbabwe and Uganda by Mabaera et al. [2]. In CIDRZ, the largest of the cohorts,

For peer review only - http://bmjopen.bmj.com/site/about/guidelines.xhtml

which we studied, monthly fluctuations in ART initiations and PTB diagnoses were the least marked, as compared to the South African and Zimbabwean sites. This can be explained by the shorter December holiday period in the Zambian health sector, in particular in the urban and peri-urban region of Lusaka, where CIDRZ is based. There, the ART programs continue to run over the festive season in December and are only closing on official public holidays. Furthermore, HIV-positive individuals seeking care at CIDRZ are mainly permanent residents in Lusaka city (as opposed to migrants from rural areas), and hence less likely to leave the city to visit their family in other parts of the country during holidays, which would contribute to delayed TB diagnoses. Similarly, Easter vacations likely explain the milder drop observed in April-May in South Africa, and to a lesser extend in Zimbabwe, but not in Zambia. Age and gender did not seem to influence the seasonal variations.

EPTB and KS diagnoses also followed variations in ART initiations in our study. We did not expect the development of EPTB to be strongly marked by climatic changes. Indeed, EPTB is less likely resulting from recent transmission than PTB, the transmission mode of which is airborne, and hence more likely to be influenced by indoor crowding and reduced ventilation during winter [1,7]. Seasonal changes in EPTB case detection have been reported [3,4,21]. However, the underlying reasons for EPTB seasonality may considerably differ from those involved in PTB. In addition, EPTB is often underdiagnosed due to lack of adequate laboratory diagnostic tools in underresourced settings [3,21]. Similarly to EPTB, KS was not expected to be influenced by climatic factors. The observed seasonal fluctuations of EPTB and KS diagnoses hence reflect variations in clinical activities, such as observed for PTB.

Several explanations of why PTB diagnoses may vary seasonally have been offered [1,3,5,7–9]. Beyond indoor crowding [1,5,9] and reduced clinical activities at specific times during the year [2], health-seeking behaviour is also influencing access to care. Health-seeking behaviour might reflect environmental barriers (long distances to the clinic, waiting time) [22,23], psychological barriers (stigma) [24], and/or clinical barriers (poor patient's health status and disabilities) [25]. Access to clinics can be problematic when temperatures are low and weather conditions are bad, which is more likely to be the case in places with harsh winters. Under these circumstances, PTB diagnosis might be delayed to spring. Professional activities or

For peer review only - http://bmjopen.bmj.com/site/about/guidelines.xhtml

For peer review only

beliefs may also prevent early healthcare seeking. Economical aspects play an important role, where care is to be fully or partly paid by patients [15]. These aspects might differently affect individuals, depending on gender or age [26–29]. However, in our study populations, trends in ART initiations and PTB diagnoses were independent from gender or age. Finally, the role of vitamin D deficiency has been debated, and studies of the relationship between vitamin D deficiency during winter and PTB are inconsistent [3,30–32].

Our study is limited by the possible under-ascertainment of TB diagnoses at ART programs, and by the fact that we could only include IeDEA-SA sites that routinely collect information on OIs. However, the included ART programs cover a wide range of geographical latitudes and different climatic seasons, with Khayelitsha and Tygerberg at the Southern tip and CIDRZ at the most equatorial end of the Southern African region. The findings of our study is restricted to HIV-positive populations, since trends in TB diagnosis and other OIs at ART programs may differ from trends in HIV-negative populations. Due the high early TB mortality, HIV-positive individuals may not reach clinical care for a TB diagnosis [33]. In addition, although ART reduces the risk of OIs significantly people living with HIV remain at a higher risk of developing TB [34,35]. Finally, social and environmental factors, including housing conditions, distance to health care facilities, access to care, disease awareness, and health-seeking behaviour may differently affect the timely diagnosis of TB in HIV-positive compared to HIV-negative populations [1,7,9,36,37]. Unfortunately, this information is not routinely collected at ART programs in the IeDEA-SA collaboration.

Conclusions

In conclusion, our study suggests that yearly trends in PTB diagnosis at ART programs in Southern Africa are mainly driven by seasonal variations in clinical activities, access to care and health-seeking behaviour, rather than climatic seasons. Our results underline the importance of offering regular care for HIV-infected individuals, including screenings for OIs such as TB at ART initiation and during follow-up visits, as part of integrated TB/HIV clinical activities at ART programs [38–41].

For peer review only - http://bmjopen.bmj.com/site/about/guidelines.xhtml

Funding

This work was supported by the National Institute of Allergy and Infectious Diseases (NIAID), the National Institute of Child Health and Human Development (NICHD), and the National Cancer Institute (NCI) under award number U01AI069924. MPF was supported by USAID 674-A-12-00029 from the United States Agency for International Development. The opinions expressed herein are those of the authors and do not necessarily reflect the views of the funder. The funder had no role in study design, data collection and analysis, decision to publish, or preparation of the manuscript.

For peer review only

Competing interests

The authors declare no conflicts of interest directly related to this project. MZ discloses the following unrelated funding sources: MZ has received grant support from the Swiss National Science Foundation and from the Swiss Cancer league which paid for PhD and postdoc positions. Furthermore, MZ has consulted for BIOTRONIK AG, Switzerland, and has projects which received unrestricted research support from following firms: AstraZeneca AG, Switzerland, Aptalis Pharma Inc., Dr. Falk Pharma GmbH, Germany, Glaxo Smith Kline AG, Nestlé S. A., Switzerland, Receptos Inc., Regeneron Inc. For the remaining authors none were declared.

Data sharing Statement

The individual contributing cohorts own the data. Consortium members may request data for analysis according to standard procedures. The consortium can also accept concept proposals from qualified researchers external to the collaboration. For more information, see www.iedea.org/working-groups/executive-committee.

Authors' contributions

MB analysed the data and completed the final draft of the manuscript. LF conceptualized the project. MZ established the methodology. KZ, SER, AB, MPF, HWP, CC and ME provided

For peer review only - http://bmjopen.bmj.com/site/about/guidelines.xhtml

input into the study design, analyses, and drafting of the paper. All authors reviewed and approved the final version of the manuscript.

Acknowledgements

We thank all participating sites and patients whose data were used in this study. We also would like to thank collaborators who contributed to recording and entering data, as well as the data centres of the IeDEA-SA collaboration for preparing the data. We thank Christopher Ritter for editorial services.

IeDEA-Southern Africa Steering Committee

Matthias Egger (co-PI), University of Bern, Switzerland; **Mary-Ann Davies** (co-PI), University of Cape Town, South Africa**; Frank Tanser**, Africa Centre for Health and Population Studies, University of Kwazulu-Natal, South Africa; **Michael Vinikoor**, Centre for Infectious Disease Research in Zambia; **Eusebio Macete**, Centro de Investigação em Saúde de Manhiça, Mozambique; **Robin Wood**, Desmond Tutu HIV Centre (Gugulethu and Masiphumelele clinics), South Africa**; Kathryn Stinson**, Khayelitsha ART Programme and Médecins Sans Frontières, South Africa; **Geoffrey Fatti**, Kheth'Impilo Programme, South Africa; **Sam Phiri**, Lighthouse Trust Clinic, Malawi; **Cleophas Chimbetete**, Newlands Clinic, Zimbabwe; **Kennedy Malisita**, Queen Elizabeth Hospital, Malawi; **Brian Eley**, Red Cross War Memorial Children's Hospital and Department of Paediatrics and Child Health, University of Cape Town, South Africa; **Jochen Ehmer**, Solidarmed, Switzerland; **Christiane Fritz**, SolidarMed SMART Programme, Lesotho; **Michael Hobbins**, SolidarMed SMART Programme, Mozambique; **Kamelia Kamenova**, SolidarMed SMART Programme, Zimbabwe; **Matthew Fox**, Themba Lethu Clinic, South Africa; **Hans Prozesky**, Tygerberg Academic Hospital, South Africa; **Karl Technau**, Empilweni Clinic, Rahima Moosa Mother and Child Hospital, South Africa; **Shobna Sawry**, Harriet Shezi Children's Clinic, Chris Hani Baragwanath Academic Hospital, South Africa.

For peer review only - http://bmjopen.bmj.com/site/about/guidelines.xhtml

References

1 Naranbat N, Nymadawa P, Schopfer K, *et al.* Seasonality of tuberculosis in an Eastern-Asian country with an extreme continental climate. *Eur Respir J* 2009;**34**:921–5. doi:10.1183/09031936.00035309

2 Mabaera B, Naranbat N, Katamba A, *et al.* Seasonal variation among tuberculosis suspects in four countries. *Int Health* 2009;**1**:53–60. doi:10.1016/j.inhe.2009.06.006

3 Willis MD, Winston CA, Heilig CM, *et al.* Seasonality of tuberculosis in the United States, 1993-2008. *Clin Infect Dis* 2012;**54**:1553–60. doi:10.1093/cid/cis235

4 Thorpe LE, Frieden TR, Laserson KF, *et al.* Seasonality of tuberculosis in India: is it real and what does it tell us? *Lancet* 2004;**364**:1613–4. doi:10.1016/S0140-6736(04)17316-9

5 Wubuli A, Li Y, Xue F, *et al.* Seasonality of active tuberculosis notification from 2005 to 2014 in Xinjiang, China. *PLOS ONE* 2017;**12**:e0180226. doi:10.1371/journal.pone.0180226

6 Margalit I, Block C, Mor Z. Seasonality of tuberculosis in Israel, 2001-2011. *Int J Tuberc Lung Dis* 2016;**20**:1588–93. doi:10.5588/ijtld.16.0306

7 Fares A. Seasonality of tuberculosis. *J Glob Infect Dis* 2011;**3**:46–55. doi:10.4103/0974-777X.77296

8 Martineau AR, Nhamoyebonde S, Oni T, *et al.* Reciprocal seasonal variation in vitamin D status and tuberculosis notifications in Cape Town, South Africa. *Proc Natl Acad Sci U S A* 2011;**108**:19013–7. doi:10.1073/pnas.1111825108

9 Wingfield T, Schumacher SG, Sandhu G, *et al.* The seasonality of tuberculosis, sunlight, vitamin d, and household crowding. *J Infect Dis* 2014;**210**:774–83. doi:10.1093/infdis/jiu121

10 Balcells ME, García P, Tiznado C, *et al.* Association of vitamin D deficiency, season of the year, and latent tuberculosis infection among household contacts. *PLOS ONE* 2017;**12**:e0175400. doi:10.1371/journal.pone.0175400

11 Corbett EL, Watt CJ, Walker N, *et al.* The growing burden of tuberculosis: global trends and interactions with the HIV epidemic. *Arch Intern Med* 2003;**163**:1009–21. doi:10.1001/archinte.163.9.1009

12 Pawlowski A, Jansson M, Skold M, *et al.* Tuberculosis and HIV co-infection. *PLoS Pathog* 2012;**8**:e1002464. doi:10.1371/journal.ppat.1002464

13 World Health Organization. Global tuberculosis report 2015. WHO 2015. http://www.who.int/tb/publications/global_report/en/

For peer review only - http://bmjopen.bmj.com/site/about/guidelines.xhtml

14 Egger M, Ekouevi DK, Williams C, *et al.* Cohort Profile: the international epidemiological databases to evaluate AIDS (IeDEA) in sub-Saharan Africa. *Int J Epidemiol* 2012;**41**:1256–64. doi:10.1093/ije/dyr080

15 Fenner L, Ballif M, Graber C, *et al.* Tuberculosis in antiretroviral treatment programs in lower income countries: availability and use of diagnostics and screening. *PloS One* 2013;**8**:e77697. doi:10.1371/journal.pone.0077697

16 Cape Town. Wikipedia. 2016.https://en.wikipedia.org/w/index.php?title=Cape_Town&oldid=748785698 (accessed 10 Nov 2016).

17 Johannesburg. Wikipedia. 2016.https://en.wikipedia.org/w/index.php?title=Johannesburg&oldid=748810210 (accessed 10 Nov 2016).

18 Harare. Wikipedia. 2016.https://en.wikipedia.org/w/index.php?title=Harare&oldid=746466670 (accessed 10 Nov 2016).

19 Lusaka. Wikipedia. 2016.https://en.wikipedia.org/w/index.php?title=Lusaka&oldid=748771985 (accessed 10 Nov 2016).

20 Altman DG. Practical Statistics for Medical Research - Relationship between two continuous variables. Chapman and Hall/CRC. 1990.

21 Top R, Boshuizen H, Dekkers A, *et al.* Similar seasonal peak in clustered and unique extra-pulmonary tuberculosis notifications: winter crowding hypothesis ruled out? *Int J Tuberc Lung Dis* 2013;**17**:1466–71. doi:10.5588/ijtld.13.0226

22 Schwitters A, Lederer P, Zilversmit L, *et al.* Barriers to Health Care in Rural Mozambique: A Rapid Ethnographic Assessment of Planned Mobile Health Clinics for ART. *Glob Health Sci Pract* 2015;**3**:109–16. doi:10.9745/GHSP-D-14-00145

23 Johnson DC, Feldacker C, Tweya H, *et al.* Factors associated with timely initiation of antiretroviral therapy in two HIV clinics in Lilongwe, Malawi. *Int J STD AIDS* 2013;**24**:42–9. doi:10.1177/0956462412472312

24 Sandelowski M, Barroso J, Voils CI. Gender, Race/Ethnicity, and Social Class in Research Reports on Stigma in HIV-Positive Women. *Health Care Women Int* 2009;**30**:273–88. doi:10.1080/07399330802694880

25 Tun W, Okal J, Schenk K, *et al.* Limited accessibility to HIV services for persons with disabilities living with HIV in Ghana, Uganda and Zambia. *J Int AIDS Soc* 2016;**19**. doi:10.7448/IAS.19.5.20829

For peer review only

For peer review only - http://bmjopen.bmj.com/site/about/guidelines.xhtml

26 Getahun H, Gunneberg C, Granich R, *et al.* HIV infection-associated tuberculosis: the epidemiology and the response. *Clin Infect Dis* 2010;**50 Suppl 3**:S201-7. doi:10.1086/651492

27 Braitstein P, Boulle A, Nash D, *et al.* Gender and the use of antiretroviral treatment in resource-constrained settings: findings from a multicenter collaboration. *J Womens Health 2002* 2008;**17**:47–55. doi:10.1089/jwh.2007.0353

28 Holmes CB, Hausler H, Nunn P. A review of sex differences in the epidemiology of tuberculosis. *Int J Tuberc Lung Dis* 1998;**2**:96–104.

29 Fenner L, Forster M, Boulle A, *et al.* Tuberculosis in HIV programmes in lower-income countries: practices and risk factors. *Int J Tuberc Lung Dis* 2011;**15**:620–7. doi:10.5588/ijtld.10.0249

30 Daley P, Jagannathan V, John KR, *et al.* Adjunctive vitamin D for treatment of active tuberculosis in India: a randomised, double-blind, placebo-controlled trial. *Lancet Infect Dis* 2015;**15**:528–34. doi:10.1016/S1473-3099(15)70053-8

31 Coussens AK, Wilkinson RJ, Hanifa Y, *et al.* Vitamin D accelerates resolution of inflammatory responses during tuberculosis treatment. *Proc Natl Acad Sci U S A* 2012;**109**:15449–54. doi:10.1073/pnas.1200072109

32 Ralph AP, Waramori G, Pontororing GJ, *et al.* L-arginine and Vitamin D Adjunctive Therapies in Pulmonary Tuberculosis: A Randomised, Double-Blind, Placebo-Controlled Trial. *PLOS ONE* 2013;**8**:e70032. doi:10.1371/journal.pone.0070032

33 Lawn SD, Harries AD, Meintjes G, *et al.* Reducing deaths from tuberculosis in antiretroviral treatment programmes in sub-Saharan Africa. *AIDS Lond Engl* 2012;**26**:2121–33. doi:10.1097/QAD.0b013e3283565dd1

34 Lawn SD, Bekker L-G, Wood R. How effectively does HAART restore immune responses to Mycobacterium tuberculosis? Implications for tuberculosis control. *AIDS Lond Engl* 2005;**19**:1113–24.

35 Lawn SD, Badri M, Wood R. Tuberculosis among HIV-infected patients receiving HAART: long term incidence and risk factors in a South African cohort. *AIDS Lond Engl* 2005;**19**:2109–16.

36 Baker M, Das D, Venugopal K, *et al.* Tuberculosis associated with household crowding in a developed country. *J Epidemiol Community Health* 2008;**62**:715–21. doi:10.1136/jech.2007.063610

37 Lönnroth K, Jaramillo E, Williams BG, *et al.* Drivers of tuberculosis epidemics: the role of risk factors and social determinants. *Soc Sci Med 1982* 2009;**68**:2240–6. doi:10.1016/j.socscimed.2009.03.041

38 Lessells RJ, Swaminathan S, Godfrey-Faussett P. HIV treatment cascade in tuberculosis patients. *Curr Opin HIV AIDS* 2015;**10**:439–46. doi:10.1097/COH.0000000000000197

For peer review only - http://bmjopen.bmj.com/site/about/guidelines.xhtml

For peer review only

39 Tweya H, Feldacker C, Gadabu OJ, *et al.* Developing a point-of-care electronic medical record system for TB/HIV co-infected patients: experiences from Lighthouse Trust, Lilongwe, Malawi. *BMC Res Notes* 2016;**9**:146. doi:10.1186/s13104-016-1943-4

40 Charles MK, Lindegren ML, Wester CW, *et al.* Implementation of Tuberculosis Intensive Case Finding, Isoniazid Preventive Therapy, and Infection Control ("Three I's") and HIV-Tuberculosis Service Integration in Lower Income Countries. *PloS One* 2016;**11**:e0153243. doi:10.1371/journal.pone.0153243

41 World Health Organization. WHO policy on collaborative TB/HIV activities: guidelines for national programmes and other stakeholders. Published Online First: 2012.http://www.who.int/tb/publications/2012/tb_hiv_policy_9789241503006/en/index.html

For peer review only

For peer review only - http://bmjopen.bmj.com/site/about/guidelines.xhtml

446 **Figure and tables**

447 **Table 1:** Characteristics of participating ART programs.

Characteristics	South Africa – Khayelitsha	South Africa – Themba Lethu	South Africa – Tygerberg	Zambia – CIDRZ	Zimbabwe – Newlands
**Geographical latitude (°)**	-34.05	-33.91	-26.12	-15.41	-17.81
**Setting**	Urban	Urban	Urban	Combination of urban and peri-urban health centres	Urban
**Level of care**	Primary	Secondary	Tertiary	Primary	Primary
**TB case definition**	Majority microbiologically confirmed, others based on clinical presentation	Microbiologically confirmed	Majority microbiologically confirmed, others based on clinical presentation	Majority microbiologically confirmed, others based on clinical presentation	Majority microbiologically confirmed, others based on clinical presentation
**Cost of TB diagnosis to patients**	Available at no cost	Available at no cost	Partial payment	Available at no cost or partial payment (depending on facility)	Available at no cost
**Co-trimoxazole for prevention**	All patients	According to CD4 cell count	According to CD4 cell count	According to CD4 cell count or to all patients (depending on facility)	According to CD4 cell count
**Integration of HIV/TB clinical services**	Same facility / same day appointments	Same facility / same day appointments	Cross referral	Cross referral	Same facility / same day appointments
**TB screening at ART enrolment**	All patients, symptomatic screening and diagnostic tests	All patients, symptomatic screening only	All patients, symptomatic screening only	All patients, symptomatic screening and diagnostic tests at selected sites	All patients, symptomatic screening only

448

449 Data sources: Ref. 15.

450 Abbreviations: ART, antiretroviral therapy; TB, tuberculosis.

451

For peer review only - http://bmjopen.bmj.com/site/about/guidelines.xhtml

Table 2: Characteristics of patients diagnosed with pulmonary tuberculosis between January 2004 and December 2014 compared to others.

Characteristics	Patients diagnosed with PTB	Other patients	All
**Age at ART start (median, IQR), years**	35 (30-41)	34 (29-41)	34 (29-41)
Missing, n (%)	*0*	*0*	*0*
**Gender, n (%)**			
Female	25,227 (51.1)	181,847 (64.4)	207,074 (62.4)
Male	24,140 (48.9)	100,420 (35.6)	124,560 (37.6)
Missing, n (%)	*0*	*0*	*0*
**CD4 cell count at ART start (median, IQR), cells/µl**	116 (52-200)	166 (87-254)	158 (80-246)
Missing, n (%)	*24,237 (48.8)*	*150,058 (53.2)*	*174,295 (52.6)*
**WHO clinical stage, n (%)**			
Stage 1 and 2	4,916 (9.9)	157,322 (55.7)	162,238 (48.9)
Stage 3 and 4	41,744 (84.6)	106,836 (37.9)	148,580 (44.8)
Unknown	2,707 (5.5)	18,053 (6.4)	20,816 (6.3)
Missing, n (%)	*0*	*56 (0.02)*	*56 (0.02)*
**Treatment program, n (%)**			
South Africa – Khayelitsha	7,357 (14.9)	25,021 (8.9)	32,378 (9.8)
South Africa – Themba Lethu	5,438 (11.0)	16,967 (6.0)	22,405 (6.8)
South Africa – Tygerberg	861 (1.7)	3,657 (1.3)	4,518 (1.4)
Zambia – CIDRZ	35,132 (71.2)	233,680 (82.8)	268,812 (81.1)
Zimbabwe – Newlands	579 (1.2)	2,942 (1.0)	3,521 (1.1)
Missing, n (%)	*0*	*0*	*0*
**Total, n (%)**	**49,367 (14.9)**	**282,267 (85.1)**	**331,634 (100)**

Abbreviations: ART, antiretroviral therapy; IQR, interquartile range; PTB, pulmonary tuberculosis; WHO, World Health Organization.

For peer review only

Table 3: Correlation coefficients between deviations of monthly counts of antiretroviral therapy enrolments from the yearly average and deviations of monthly counts of pulmonary tuberculosis, extrapulmonary tuberculosis, and Kaposi sarcoma diagnosis from the yearly averages. Median monthly event counts (2000-2014) and Pearson's pairwise correlation coefficients are shown.

**ART program**	**ART enrolments**	**PTB diagnoses**			**EPTB diagnoses**			**KS diagnoses**		
	Median (IQR)	Median (IQR)	Correlation (95% CI)	p-value	Median (IQR)	Correlation (95% CI)	p-value	Median (IQR)	Correlation (95% CI)	p-value
South Africa – Khayelitsha	252 (236-265)	58 (46-64)	0.27 (0.11-0.42)	0.002	26 (24-30)	0.20 (0.03-0.36)	0.019	2 (1-2)	0.08 (-0.11-0.25)	0.42
South Africa – Themba Lethu	171 (161-180)	41 (37-44)	0.22 (0.05-0.38)	0.010	16 (14-17)	0.15 (-0.03-0.31)	0.097	1 (1-1)	0.02 (-0.16-0.19)	0.86
South Africa – Tygerberg	34 (34-37)	7 (6-8)	0.19 (0.02-0.35)	0.028	3 (3-3)	0.09 (-0.09-0.25)	0.33	0.3 (0.2-0.5)	0.11 (-0.06-0.28)	0.22
Zambia – CIDRZ	2163 (2095-2233)	275 (259-301)	0.82 (0.75-0.87)	<0.001	9 (9-10)	0.54 (0.40-0.65)	<0.001	29 (28-31)	0.68 (0.57-0.77)	<0.001
Zimbabwe – Newlands	31 (29-34)	5 (4-6)	0.20 (0.02-0.37)	0.029	2 (2-2)	0.23 (0.06-0.40)	0.011	0.2 (0.1-0.4)	0.22 (0.05-0.39)	0.014
**All**	**170 (34-264)**	**40 (6-63)**	**0.28 (0.21-0.35)**	**<0.001**	**9 (3-17)**	**0.25 (0.18-0.32)**	**<0.001**	**1 (0-2)**	**0.14 (0.07-0.22)**	**<0.001**

Abbreviations: ART, antiretroviral therapy; 95% CI, 95% confidence interval; EPTB, extrapulmonary tuberculosis; IQR, interquartile range; KS, Kaposi sarcoma; PTB, pulmonary tuberculosis.

For peer review only - http://bmjopen.bmj.com/site/about/guidelines.xhtml

Table 4: Regression coefficients between deviations of monthly counts of pulmonary tuberculosis, extrapulmonary tuberculosis, or Kaposi sarcoma diagnoses and deviations of monthly counts of antiretroviral therapy enrolments from the yearly averages (unadjusted); adjusted for the deviations of monthly air temperatures to yearly average air temperatures at the ART program (“adjusted for temperature”). The overall regression model was also adjusted for the latitude of the ART program’s location (“adjusted for temperature and latitude”). Monthly air temperatures are based on 1961-1990 averages.

ART program	PTB diagnoses		EPTB diagnoses		KS diagnoses	
	Coefficient (95% CI)	p-value	Coefficient (95% CI)	p-value	Coefficient (95% CI)	p-value
**South Africa – Khayelitsha**						
Unadjusted	0.33 (0.13-0.53)	0.002	0.28 (0.05-0.51)	0.019	0.30 (-0.43-1.03)	0.416
Adjusted for temperature	0.30 (0.09-0.51)	0.005	0.27 (0.03-0.51)	0.027	0.44 (-0.31-1.12)	0.248
**South Africa – Themba Lethu**						
Unadjusted	0.16 (0.04-0.28)	0.010	0.21 (-0.04-0.45)	0.097	0.07 (-0.74-0.88)	0.860
Adjusted for temperature	0.17 (0.05-0.29)	0.005	0.21 (-0.03-0.46)	0.089	0.10 (-0.71-0.92)	0.805
**South Africa – Tygerberg**						
Unadjusted	0.41 (0.04-0.78)	0.028	0.20 (-0.20-0.60)	0.328	0.82 (-0.49-2.14)	0.218
Adjusted for temperature	0.41 (0.05-0.78)	0.028	0.21 (-0.20-0.62)	0.307	0.92 (-0.39-2.23)	0.169
**Zambia – CIDRZ**						
Unadjusted	0.84 (0.74-0.95)	<0.001	1.39 (1.00-1.78)	<0.001	0.91 (0.73-1.08)	<0.001
Adjusted for temperature	0.83 (0.72-0.94)	<0.001	1.40 (1.00-1.80)	<0.001	0.90 (0.72-1.08)	<0.001
**Zimbabwe – Newlands**						
Unadjusted	0.29 (0.03-0.54)	0.029	0.47 (0.11-0.83)	0.011	1.32 (0.27-2.38)	0.014
Adjusted for temperature	0.29 (0.037-0.55)	0.025	0.47 (0.12-0.84)	0.010	1.31 (0.26-2.36)	0.015
**All**						
Unadjusted	0.37 (0.27-0.47)	<0.001	0.50 (0.35-0.65)	<0.001	0.75 (0.35-1.16)	<0.001
Adjusted for temperature	0.38 (0.28-0.48)	<0.001	0.51 (0.36-0.66)	<0.001	0.77 (0.36-1.18)	<0.001
Adjusted for temperature and latitude	0.38 (0.28-0.48)	<0.001	0.51 (0.36-0.66)	<0.001	0.77 (0.36-1.18)	<0.001

For peer review only

Abbreviations: EPTB, extrapulmonary tuberculosis; 95% CI, 95% confidence interval; KS, Kaposi sarcoma; PTB, pulmonary tuberculosis.

Figure Legends

Figure 1: Selection of patients for the analysis.

Figure 2: Aggregated averages in monthly counts of patients newly started on antiretroviral therapy and monthly counts of pulmonary tuberculosis diagnosis (aggregate averages 2004-2014, logarithmic scale). Dashed horizontal lines indicate overall monthly means.

Figure 3: Relationship between pulmonary tuberculosis (PTB) diagnosis and geographical latitude: regression coefficients between deviations of monthly counts of PTB diagnoses and deviations of monthly counts of antiretroviral therapy enrolments from the yearly averages (adjusted for air temperature) according to the geographical latitude of the ART program's location.

For peer review only

For peer review only - http://bmjopen.bmj.com/site/about/guidelines.xhtml

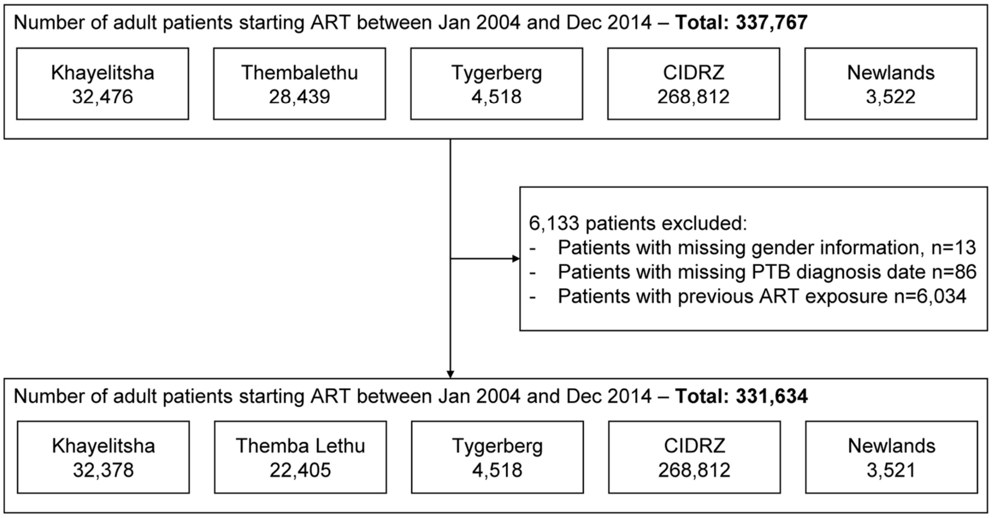

Figure 1: Selection of patients for the analysis.
Abbreviations: ART, antiretroviral therapy; PTB, pulmonary tuberculosis

146x76mm (300 x 300 DPI)

review only

For peer review only - http://bmjopen.bmj.com/site/about/guidelines.xhtml

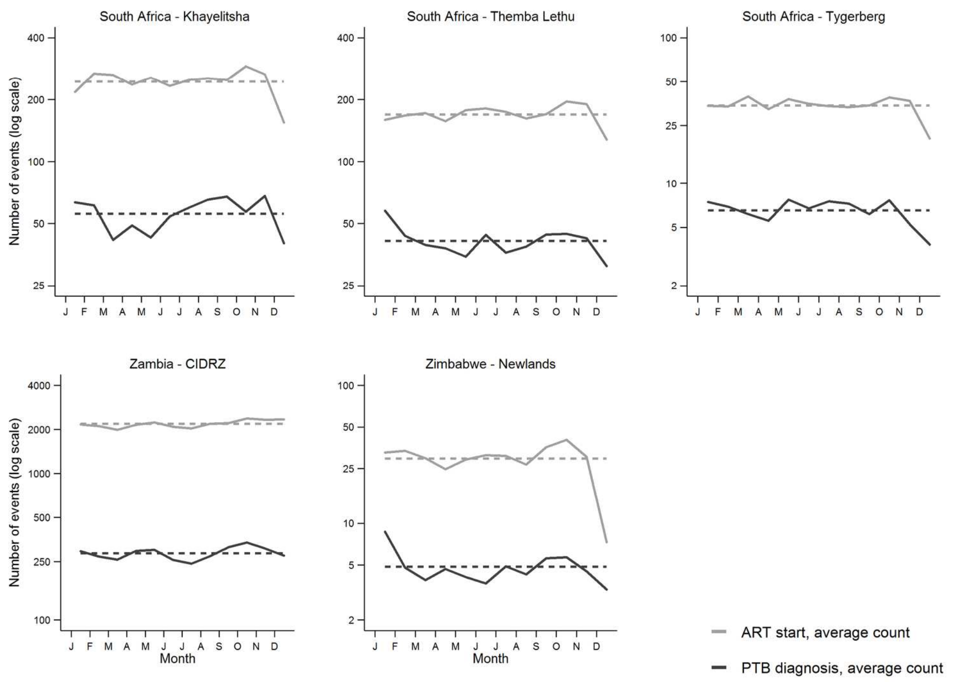

Figure 2: Aggregated averages in monthly counts of patients newly started on antiretroviral therapy and monthly counts of pulmonary tuberculosis diagnosis (aggregate averages 2004-2014, logarithmic scale). Dashed horizontal lines indicate overall monthly means.
Abbreviations: ART, antiretroviral therapy; PTB, pulmonary tuberculosis

182x132mm (300 x 300 DPI)

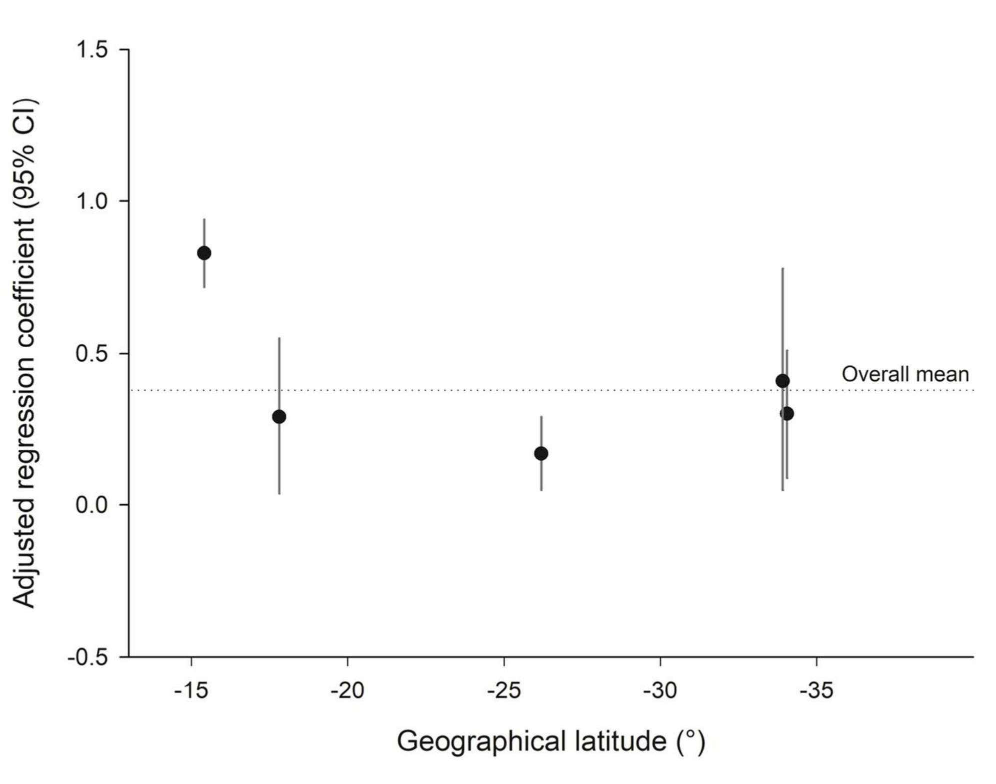

Figure 3: Relationship between pulmonary tuberculosis (PTB) diagnosis and geographical latitude: regression coefficients between deviations of monthly counts of PTB diagnoses and deviations of monthly counts of antiretroviral therapy enrolments from the yearly averages (adjusted for air temperature) according to the geographical latitude of the ART program's location.

115x89mm (300 x 300 DPI)

Supplementary File

Supplementary Figure 1: Quarterly counts of patients newly started on antiretroviral therapy, and quarterly counts of pulmonary tuberculosis diagnoses between 2004 and 2014.

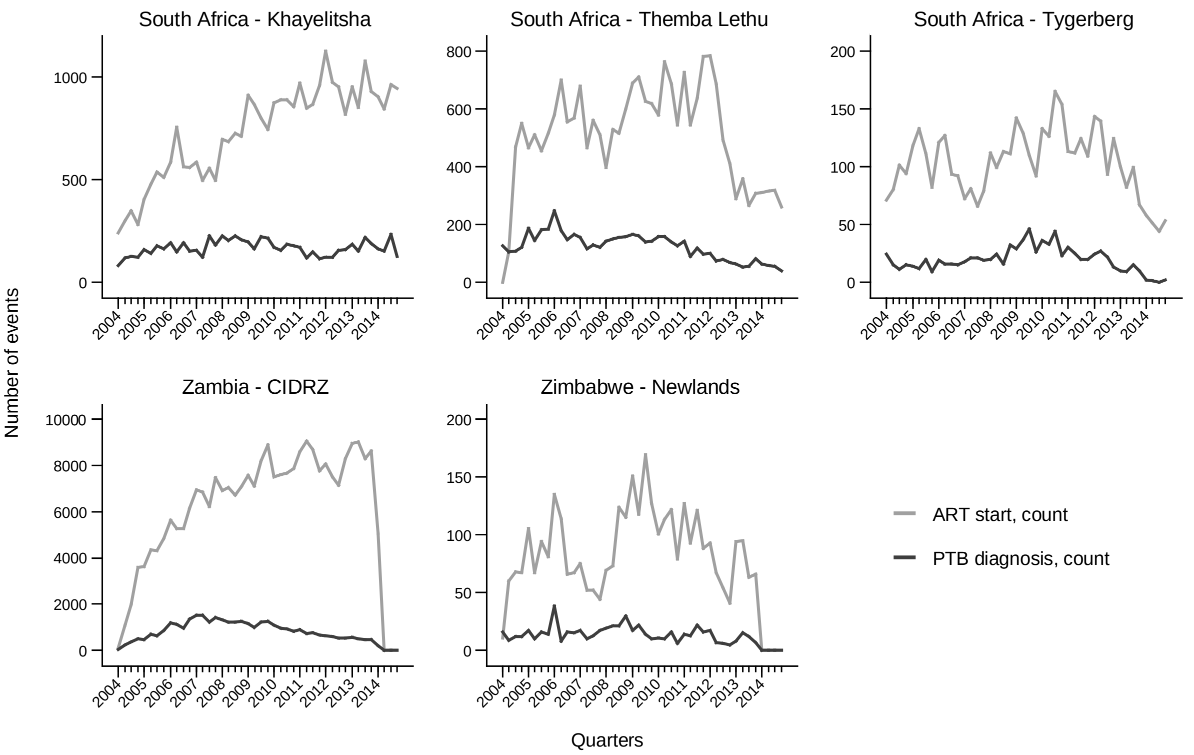

Abbreviations: ART, antiretroviral therapy; PTB, pulmonary tuberculosis

For peer review only - http://bmjopen.bmj.com/site/about/guidelines.xhtml

Supplementary Figure 2: Aggregated averages in monthly counts of patients newly started on antiretroviral therapy and monthly counts of pulmonary tuberculosis diagnosis (study period 2004-2014), by gender (**panel A**) and by age groups (**panel B**). Horizontal lines indicate the overall monthly mean of ART enrolment events or PTB diagnosis events.

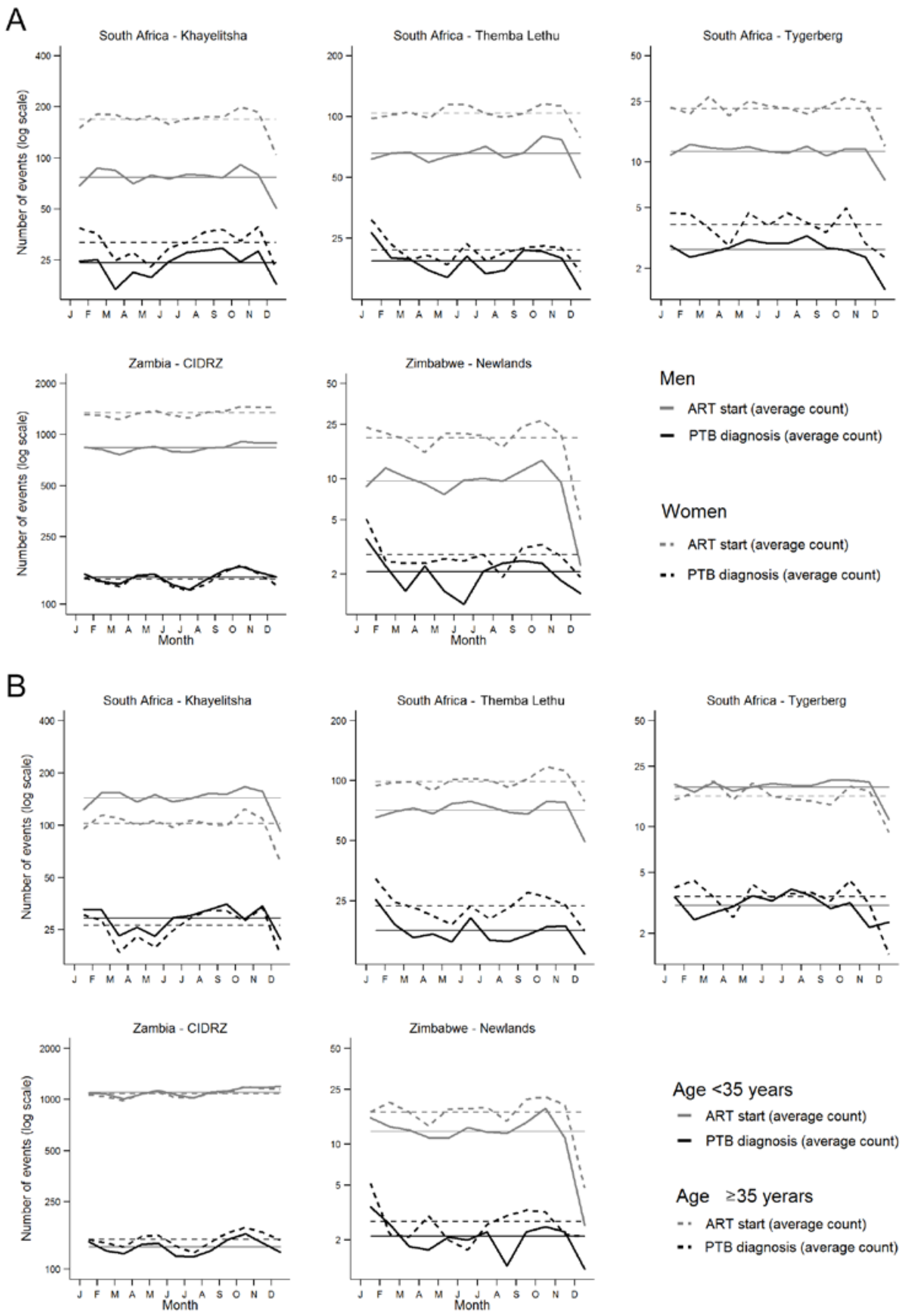

Abbreviations: ART, antiretroviral therapy; PTB, pulmonary tuberculosis.

Supplementary Figure 3: Mean deviations of monthly counts in antiretroviral therapy initiations and pulmonary tuberculosis diagnosis from the corresponding yearly averages.

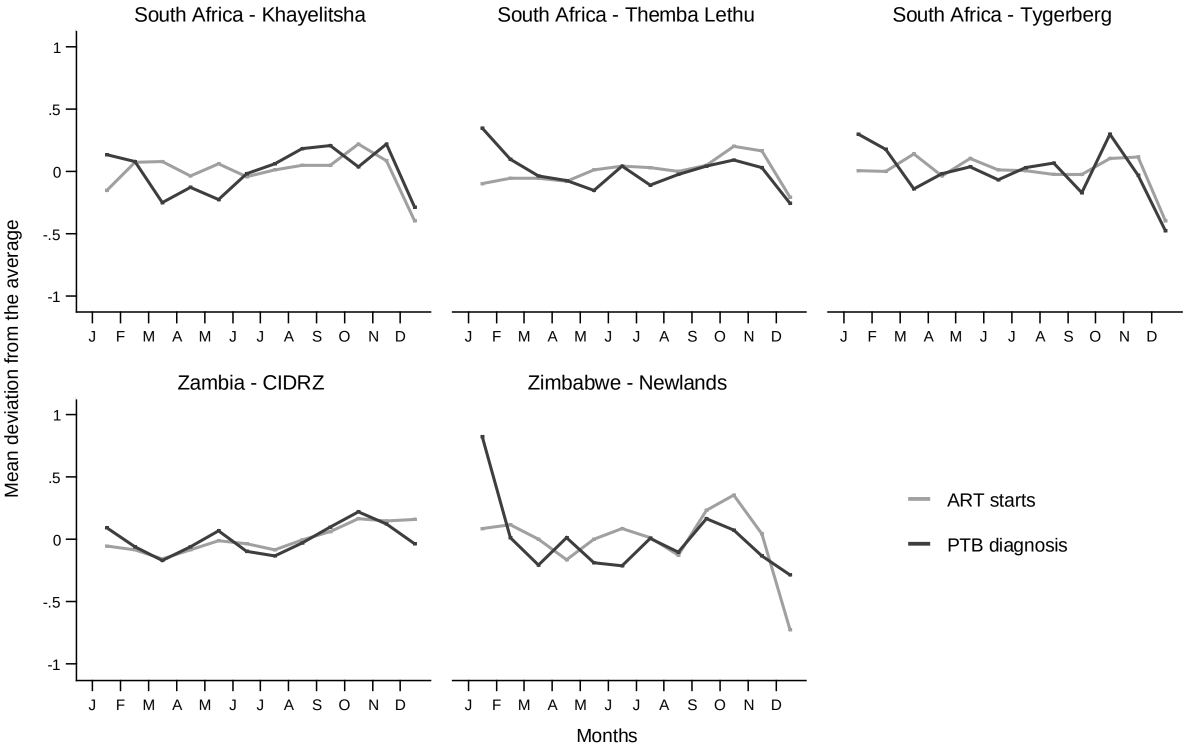

Abbreviations: ART, antiretroviral therapy; PTB, pulmonary tuberculosis

STROBE Statement—Checklist of items that should be included in reports of ***cohort studies***

Manuscript: Seasonal variations in tuberculosis diagnosis among HIV-coinfected individuals in Southern Africa: an observational cohort study at antiretroviral treatment programs

	Item No	Recommendation	Manuscript page number
**Title and abstract**	1	(*a*) Indicate the study's design with a commonly used term in the title or the abstract	**1, 3**
		(*b*) Provide in the abstract an informative and balanced summary of what was done and what was found	**3**
**Introduction**			
Background/rationale	2	Explain the scientific background and rationale for the investigation being reported	**5**
Objectives	3	State specific objectives, including any prespecified hypotheses	**5**
**Methods**			
Study design	4	Present key elements of study design early in the paper	**6-7**
Setting	5	Describe the setting, locations, and relevant dates, including periods of recruitment, exposure, follow-up, and data collection	**6**
Participants	6	(*a*) Give the eligibility criteria, and the sources and methods of selection of participants. Describe methods of follow-up	**6**
		(*b*) For matched studies, give matching criteria and number of exposed and unexposed	**NA**
Variables	7	Clearly define all outcomes, exposures, predictors, potential confounders, and effect modifiers. Give diagnostic criteria, if applicable	**6-7**
Data sources/ measurement	8*	For each variable of interest, give sources of data and details of methods of assessment (measurement). Describe comparability of assessment methods if there is more than one group	**6-7**
Bias	9	Describe any efforts to address potential sources of bias	**6-7**
Study size	10	Explain how the study size was arrived at	**NA**
Quantitative variables	11	Explain how quantitative variables were handled in the analyses. If applicable, describe which groupings were chosen and why	**6-7**
Statistical methods	12	(*a*) Describe all statistical methods, including those used to control for confounding	**6-7**
		(*b*) Describe any methods used to examine subgroups and interactions	**6-7**
		(*c*) Explain how missing data were addressed	**6-7**
		(*d*) If applicable, explain how loss to follow-up was addressed	**NA**
		(*e*) Describe any sensitivity analyses	**NA**
**Results**			
Participants	13*	(a) Report numbers of individuals at each stage of study—eg numbers potentially eligible, examined for eligibility, confirmed eligible, included in the study, completing follow-up, and analysed	**8 + Fig. 1**
		(b) Give reasons for non-participation at each stage	**Fig. 1**
		(c) Consider use of a flow diagram	**Fig. 1**
Descriptive data	14*	(a) Give characteristics of study participants (eg demographic, clinical, social) and information on exposures and potential	**8 + table 2**

For peer review only

		confounders	
		(b) Indicate number of participants with missing data for each variable of interest	**Fig. 1+ table 2**
		(c) Summarise follow-up time (eg, average and total amount)	**8**
Outcome data	15*	Report numbers of outcome events or summary measures over time	**8-9**
Main results	16	(*a*) Give unadjusted estimates and, if applicable, confounder-adjusted estimates and their precision (eg, 95% confidence interval). Make clear which confounders were adjusted for and why they were included	**8-9**
		(*b*) Report category boundaries when continuous variables were categorized	**NA**
		(*c*) If relevant, consider translating estimates of relative risk into absolute risk for a meaningful time period	**NA**
Other analyses	17	Report other analyses done—eg analyses of subgroups and interactions, and sensitivity analyses	**8-9**
**Discussion**			
Key results	18	Summarise key results with reference to study objectives	**10**
Limitations	19	Discuss limitations of the study, taking into account sources of potential bias or imprecision. Discuss both direction and magnitude of any potential bias	**12**
Interpretation	20	Give a cautious overall interpretation of results considering objectives, limitations, multiplicity of analyses, results from similar studies, and other relevant evidence	**10-12**
Generalisability	21	Discuss the generalisability (external validity) of the study results	**12**
**Other information**			
Funding	22	Give the source of funding and the role of the funders for the present study and, if applicable, for the original study on which the present article is based	**13**

*Give information separately for exposed and unexposed groups.

Note: An Explanation and Elaboration article discusses each checklist item and gives methodological background and published examples of transparent reporting. The STROBE checklist is best used in conjunction with this article (freely available on the Web sites of PLoS Medicine at http://www.plosmedicine.org/, Annals of Internal Medicine at http://www.annals.org/, and Epidemiology at http://www.epidem.com/). Information on the STROBE Initiative is available at http://www.strobe-statement.org.

For peer review only

For peer review only - http://bmjopen.bmj.com/site/about/guidelines.xhtml
